# Prediction performance and fairness heterogeneity in cardiovascular risk models

**DOI:** 10.1038/s41598-022-16615-3

**Published:** 2022-07-22

**Authors:** Uri Kartoun, Shaan Khurshid, Bum Chul Kwon, Aniruddh P. Patel, Puneet Batra, Anthony Philippakis, Amit V. Khera, Patrick T. Ellinor, Steven A. Lubitz, Kenney Ng

**Affiliations:** 1 Center for Computational Health, IBM Research, 314 Main St., Cambridge, MA 02142 USA; 2 Cardiovascular Disease Initiative, Broad Institute of the Massachusetts Institute of Technology and Harvard University, Cambridge, MA USA; 3 Demoulas Center for Cardiac Arrhythmias, Massachusetts General Hospital, Boston, MA USA; 4 Division of Cardiology, Massachusetts General Hospital, Boston, MA USA; 5 Data Sciences Platform, Broad Institute of the Massachusetts Institute of Technology and Harvard University, Cambridge, MA USA

**Keywords:** Outcomes research, Medical research, Predictive markers

## Abstract

Prediction models are commonly used to estimate risk for cardiovascular diseases, to inform diagnosis and management. However, performance may vary substantially across relevant subgroups of the population. Here we investigated heterogeneity of accuracy and fairness metrics across a variety of subgroups for risk prediction of two common diseases: atrial fibrillation (AF) and atherosclerotic cardiovascular disease (ASCVD). We calculated the Cohorts for Heart and Aging in Genomic Epidemiology Atrial Fibrillation (CHARGE-AF) score for AF and the Pooled Cohort Equations (PCE) score for ASCVD in three large datasets: Explorys Life Sciences Dataset (Explorys, n = 21,809,334), Mass General Brigham (MGB, n = 520,868), and the UK Biobank (UKBB, n = 502,521). Our results demonstrate important performance heterogeneity across subpopulations defined by age, sex, and presence of preexisting disease, with fairly consistent patterns across both scores. For example, using CHARGE-AF, discrimination declined with increasing age, with a concordance index of 0.72 [95% CI 0.72–0.73] for the youngest (45–54 years) subgroup to 0.57 [0.56–0.58] for the oldest (85–90 years) subgroup in Explorys. Even though sex is not included in CHARGE-AF, the statistical parity difference (i.e., likelihood of being classified as high risk) was considerable between males and females within the 65–74 years subgroup with a value of − 0.33 [95% CI − 0.33 to − 0.33]. We also observed weak discrimination (i.e., < 0.7) and suboptimal calibration (i.e., calibration slope outside of 0.7–1.3) in large subsets of the population; for example, all individuals aged 75 years or older in Explorys (17.4%). Our findings highlight the need to characterize and quantify the behavior of clinical risk models within specific subpopulations so they can be used appropriately to facilitate more accurate, consistent, and equitable assessment of disease risk.

## Introduction

Variability in the accuracy of models used to classify cardiovascular disease (CVD) risk has frequently been reported^1,2^, with findings highlighting that performance appears to vary on the basis of sex^3^, race (in the US^4–6^ and out of the US^7–9^), and the presence of specific clinical factors^10,11^. With the continued growth of large collections of electronic health records (EHRs) accessible for research purposes, it is now possible to more thoroughly explore and better understand the performance heterogeneity of risk estimators, including within more refined subgroups.

CVD risk models are commonly used to prioritize individuals for preventive counseling (e.g., weight loss, alcohol cessation) and therapies (e.g., cholesterol-lowering medication). For atherosclerotic CVD (ASCVD), risk estimation using the Pooled Cohort Equations (PCE) is recommended by U.S. guidelines for determining whether individuals without established ASCVD should be considered for cholesterol-lowering therapy^12^. For atrial fibrillation (AF), in which the presence of arrhythmia is associated with an increased risk of stroke and heart failure (HF), risk estimation may also prioritize individuals for screening to detect asymptomatic disease^13,14^. The Cohorts for Heart and Aging Research in Genomic Epidemiology AF (CHARGE-AF) score^15,16^ has consistently demonstrated good predictive performance for incident AF risk across multiple community cohorts^17,18^ and EHR-based repositories^19^.

Leveraging three large and distinct datasets, one from a prospective cohort and two from electronic health records, in total covering millions of individuals, we aimed to quantify the robustness of established models used to predict risk for AF and ASCVD. Specifically, we deployed the CHARGE-AF and PCE scores within subpopulations defined by clinically relevant strata (e.g., age, sex, and presence of relevant diseases at baseline), and quantified model performance, including discrimination, calibration, and fairness metrics, assessing for important and consistent patterns of heterogeneity^20^.

## Methods

### Data sources

A high-level summary of our methodology is illustrated in Supplementary Fig. 1. We analyzed 3 independent data sources: the Explorys Dataset, Mass General Brigham (MGB), and the UK Biobank (UKBB).

The Explorys Dataset is comprised of the healthcare data of over 21 million individuals, pooled from different healthcare systems with distinct EHRs that have been previously used for medical research^19,21,22^. Data were statistically de-identified^23^, standardized, normalized using common ontologies, and made searchable after being uploaded to a Health Insurance Portability and Accountability Act-enabled platform. The data included EHR entries for all patients who were seen between January 1, 1999, and December 31, 2020.

MGB is a large healthcare network serving the New England region of the US. We utilized the Community Care Cohort Project^24^, an EHR dataset comprising over 520,000 individuals who received longitudinal primary within the MGB system, which includes 7 academic and community hospitals with associated outpatient clinics.

The UKBB is a prospective cohort of over 500,000 participants enrolled during 2006–2010^25^. Briefly, approximately 9.2 million individuals aged 40–69 years living within 25 miles of 22 assessment centers in the UK were invited, and 5.4% participated in the baseline assessment. Questionnaires and physical measures were collected at recruitment, and all participants are followed for outcomes through linkage to national health-related datasets provided by the Health & Social Care Information Centre, the Patient Episode Database for Wales, and by Scottish Morbidity Records^26^. We confirm that all methods were performed in accordance with the relevant guidelines and regulations.

### Cohort construction

To ensure adequate data ascertainment and follow-up, we included individuals in Explorys with at least two outpatient encounters greater than or equal to 2 years apart^27^. Individuals in the MGB dataset had at least one pair of primary care office visits 1–3 years apart. We included all individuals who enrolled in the UKBB study, excluding those who subsequently withdrew consent.

In Explorys, the start of follow-up was defined as the first encounter following the second qualifying outpatient encounter. In MGB, the start of follow-up was defined as the second office visit of the earliest qualifying pair. In UKBB, the start of follow-up was the initial assessment visit. In each dataset, baseline variables were defined at or before the start of follow-up. Individuals with missing data for AF risk estimation at baseline were excluded. We refer to the AF analysis sets as the “AF Subsets”. We defined the ASCVD analysis set analogously, with the exclusion of individuals with missing data needed to calculate the PCE score (“ASCVD Subsets”). Full details of the cohort construction for the 3 datasets are shown in Supplementary Tables I–VI.

### Clinical factors

Age, sex, race, and smoking status were defined using EHR fields in Explorys and MGB and were self-reported at the initial assessment visit in UKBB. Height, weight, blood pressure, total cholesterol, and high-density lipoprotein cholesterol values were similarly extracted from the EHR in MGB and Explorys and measured at the baseline assessment in UKBB^19,28^. For patients with multiple eligible values in the baseline period, only the most recent was used. Smoking status was classified as present or absent, and race was classified as White or Black. Since dedicated PCE models are available only for White and Black individuals, as performed previously^29^ the models developed for Black individuals were utilized for individuals identifying as Black, while the models developed for White individuals were utilized for individuals of all other races. The presence of clinical comorbidities was ascertained using diagnostic (International Classification of Diseases-9th [ICD-9] and -10th [ICD-10] revisions) and procedural (Current Procedural Terminology, CPT) codes, either extracted from the EHR (Explorys and MGB), or from linked national health record data (UKBB). All covariates were used in accordance with the CHARGE-AF and PCE definitions^12,16,30^. Clinical factor definitions for all outcomes and covariates appear in Supplementary Table VII.

### Follow-up and outcome definitions

The primary outcomes were the 5-year incident AF (for the AF Subsets), and the 10-year incident ASCVD (for the ASCVD Subsets). In the EHR samples, incident AF was defined using a previously validated EHR-based AF ascertainment algorithm (positive predictive value 92%), with the exception that electrocardiographic criteria were not used in Explorys given absence of electrocardiogram reports^31^. In the UKBB, AF was defined using a previously published set of self-reported data and diagnostic and procedural codes, which had been previously validated in an external dataset with a positive predictive value of 92%^32^. Incident ASCVD was defined as a composite of myocardial infarction (MI) and stroke, each defined using diagnosis codes^33^. The codes used to define ASCVD in UKBB and Explorys have been previously published^19,32^, and those used in MGB have been previously validated with positive predictive value of ≥ 85%^27^. Outcome definitions are shown in Supplementary Table VII.

All models were censored at last follow-up or the end of the relevant prediction window (i.e., 5 years for CHARGE-AF and 10 years for the PCE). Last follow-up was defined as the last office visit or hospital encounter in Explorys, last EHR encounter in MGB (or administrative censoring date of August 31, 2019), and date of last available linked hospital data in UKBB. Since date of death is known in UKBB and MGB, follow-up was also censored at death in these analyses. However, since the precise date of death was not available in Explorys, we did not attempt to censor death (i.e., death was presumed to occur after the last office visit or hospital encounter).

### Subgroup types

Per the original design of the PCE, we assessed the 4 sex- and race-specific models within their respective populations (Black women, Black men, White women, White men). All populations were further stratified into 10-year age ranges. These age-based analyses included 6 age strata for CHARGE-AF (45–54, 55–64, 65–74, 75–84, 85–90, and all) and 5 age strata for PCE (40–49, 50–59, 60–69, 70–79, and all). In the AF analyses, we evaluated the following additional subgroups: females, males, Black race, White race, prevalent HF, and prevalent stroke. In the PCE analyses, we also evaluated prevalent HF.

### Quantification of model performance

We computed incidence rates for each outcome, reported per 1000 patient years (1 K PY). For each risk score and subgroup, we assessed the association between the risk score and its respective outcome using Cox proportional hazards regression, with 5-year AF as the outcome of interest for CHARGE-AF and 10-year ASCVD as the outcome of interest for PCE. Since the CHARGE-AF and PCE models did not account for death as a competing risk, date of death is not available in Explorys, and the proportion of individuals who died prior to the end of follow-up was low in both UKBB (AF 1.6%, PCE 3.1%) and MGB (AF 0.3%, PCE 0.4%), we did not model the competing risk of death. Hazard ratios were scaled by the within-sample standard deviation (SD) of the linear predictor of each score for comparability (Standardized Hazard Ratio [SHR]). Therefore, the SHR reflects the relative increase in event hazard observed with a 1-SD increase in the respective linear predictor. We also assessed the discrimination of each score by calculating Harrell’s concordance index. We compared calibration slopes, defined as the beta coefficient of a univariable Cox proportional hazards model with the prediction target as the outcome and the linear predictor of the respective risk score as the sole covariate, where an optimally calibrated slope has a value of one^34^. To calculate 95% confidence intervals, we applied bootstrap resampling with 100 replicates.

For the purposes of identifying subgroups in which performance was particularly suboptimal, we utilized a concordance index of < 0.7. For calibration, in the absence of a consensus definition of a poor calibration slope, we utilized arbitrary calibration slope thresholds of < 0.7 (general tendency to overestimate) or > 1.3 (general tendency to underestimate) to define suboptimal calibration.

To assess performance heterogeneity beyond traditional model metrics, we calculated fairness measures, including statistical parity difference, true positive rate difference, and true negative rate difference^35^. Such measures assess fairness within the context of a protected attribute (e.g., sex, race). Statistical parity difference represents differences in the predicted risk according to the score. True positive and negative rates represent differences in sensitivity and specificity. These analyses focused on subgroups most likely to be affected by potential unfairness, including age, sex (female and male) and race (Black and White). A score is considered potentially unfair if it exhibits unexplained performance variation across different subpopulations. Fairness measures may be independent of traditional model metrics for accuracy (e.g., a score may provide very good discrimination within a subpopulation but could still be unfair).

For these analyses, the CHARGE-AF and PCE scores were converted to event probabilities using their published equations^12,15^. Where fairness metrics required application of binary risk cutoffs (i.e., true positive rate difference and false positive rate difference), we defined high AF risk as estimated 5-year AF risk ≥ 5.0% using CHARGE-AF^19,36^ and high ASCVD risk as estimated 10-year ASCVD risk ≥ 7.5%^1,3,4,30^.

All analyses were performed using R version 3.6, including the “survival,” “rms,” “data.table,” and “prodlim” packages^37^.

## Results

A summary of baseline characteristics for the three datasets and their associated two outcomes is shown in Table 1, including mean (SD) for continuous measurements, percentage for binary attributes, and follow-up durations. For brevity, only the PCE model with the largest sample size (female-White; n = 1,763,103) is described in the sections below; results for all four PCE models are presented in Supplementary Table VIII and Supplementary Fig. 2.

**Table 1:** Baseline characteristics.

	Incident AF (5 years)			Incident ASCVD (10 years)		
	Explorys (N = 4,750,660)	UKBB (N = 445,329)	MGB (N = 174,644)	Explorys (N = 3,656,680)	UKBB (N = 408,154)	MGB (N = 198,184)
N events	196,252	7404	7877	346,159	10,906	10,201
Median follow-up, years (Q1, Q3)	3.6 (1.6, 5.0)	5.0 (5.0, 5.0)	5.0 (2.3, 5.0)	3.8 (1.8, 6.6)	8.9 (8.2, 9.7)	6.8 (2.6, 10.0)
**Characteristics**	**% or mean (SD)**					
Female (%)	56.7	55.0	60.9	55.9	54.8	58.8
Age (years)	62.6 (10.8)	58.4 (7.0)	60.9 (10.0)	59.0 (10.7)	56.9 (8.1)	57.0 (10.3)
White race (%)	84.2	94.7	79.6	87.4	98.4	78.1
Smoking (%)	17.3	10.7	8.0	18.7	10.4	7.4
SBP (mmHg)	131 (18)	139 (19)	128 (17)	129 (17)	139 (20)	126 (17)
DBP (mmHg**)**	77 (11)	83 (10)	76 (10)	DBP, Height, and Weight were not necessary to calculate PCE scores		
Height (cm)	168.5 (10.9)	168.2 (9.2)	166.6 (10.4)			
Weight (kg)	86.1 (22.1)	77.9 (15.8)	79.4 (19.5)			
HDL (US: mg/dL; UK: mmol/L)	HDL and TC were not necessary to calculate CHARGE-AF scores			51 (17)	1.46 (0.4)	57 (18)
TC (US: mg/dL; UK: mmol/L)				189 (42)	5.7 (1.1)	195 (39)
Hypertensive therapy (%)	50.1	30.5	44.8	52.8	27.9	39.3
Diabetes (%)	21.3	2.5	16.0	21.4	5.0	14.8
Heart failure (%)	3.7	0.4	1.9	3.5	0.3	1.6

### Association between age and incidence of AF and ASCVD

**Figure 1:**
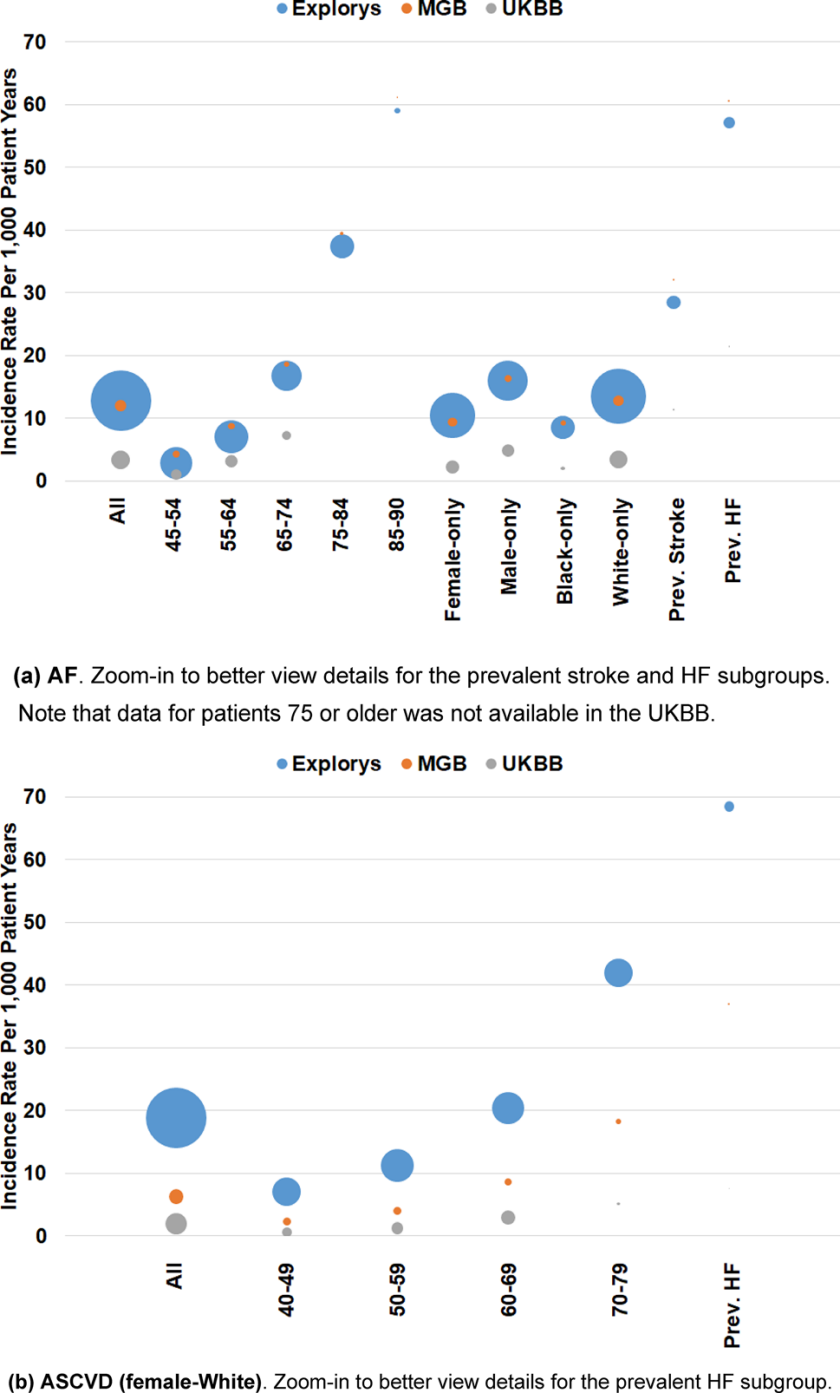
Incidence rates per 1 K PY and population sizes. All population and subpopulation sizes and exact incidence rates are provided in Supplementary Table IX.

As shown in Fig. 1A (AF) and B (ASCVD), incidence rate increased with age in each dataset. Explorys and MGB showed similar incidence rates in each age group, whereas UKBB participants had substantially lower AF incidence. Similarly, ASCVD incidence rate increased with age, but higher in Explorys compared to MGB and the UKBB. The effect of age on ASCVD within each of the four PCE groups is shown in Supplementary Table VIII.

### Performance heterogeneity of CHARGE-AF

We observed that a variety of subgroups were affected by limited discrimination, suboptimal calibration, or both (Supplementary Tables X and XI); for example, discrimination was lower than 0.7 and calibration slope was out of the 0.7–1.3 range among individuals aged 75 years or older (17.4% in Explorys, 10.6% in MGB). Discrimination and calibration also met criteria for poor performance among patients with prevalent HF (3.7% in Explorys, 1.9% in MGB).

**Figure 2:**
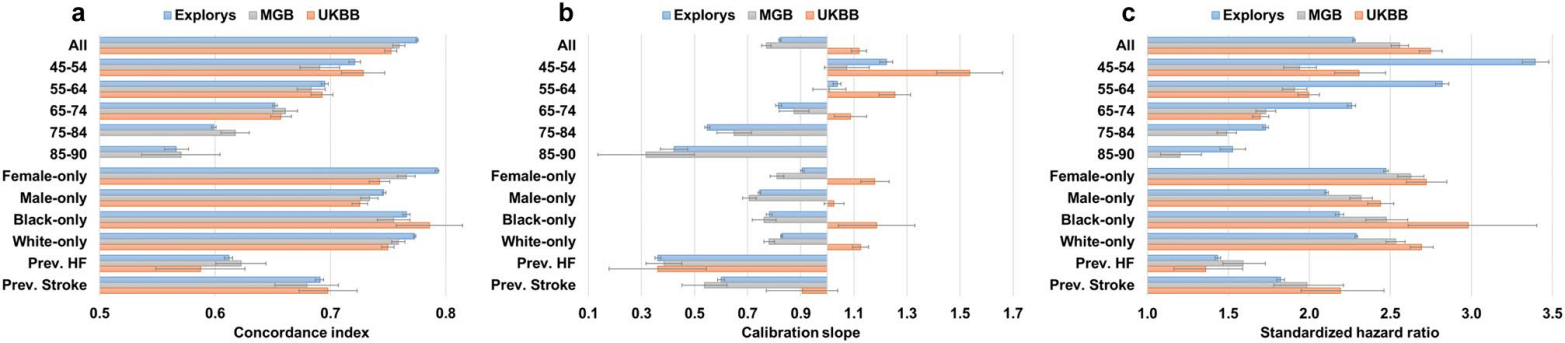
Performance measures for CHARGE-AF. Prev. = Prevalence; HF = Heart failure.

Figure 2 summarizes performance measures for the CHARGE-AF score. Discrimination consistently decreased with increased age (Fig. 2A); for example, discrimination declined with increasing age from concordance index of 0.721 [95% CI 0.716–0.726] for the youngest (45–54 years) subgroup to 0.566 [0.556–0.577], for the oldest (85–90 years) subgroup in Explorys. Discrimination was higher for females than for males, consistent with prior findings^1,16,19,36^, whereas differences across White versus Black race were minor. Discrimination was substantially lower among individuals with prevalent HF and stroke.

We also observed miscalibration within subgroups of age. For all 3 datasets calibration slopes decreased with increasing age, reflecting a general tendency toward underestimation at younger ages and overestimation at older ages (Fig. 2B); for example, in Explorys, values declined from 1.222 [95% CI 1.198–1.246] for the youngest (45–54 years) subgroup to 0.422 [0.371–0.474] for the oldest (85–90 years) subgroup.

The strength of association between the CHARGE-AF score and incident AF (as measured using SHRs) decreased with older age (Fig. 2C); for example, SHR declined from 3.395 [95% CI 3.315–3.477] for the youngest (45–54 years) subgroup to 1.526 [1.449–1.606] for the oldest (85–90 years) subgroup in Explorys. Within strata defined by sex and race, SHRs were highest in the UKBB, followed by MGB and Explorys. SHRs were substantially lower among individuals with prevalent HF and stroke.

### Unfair behaviors for CHARGE-AF

**Figure 3:**
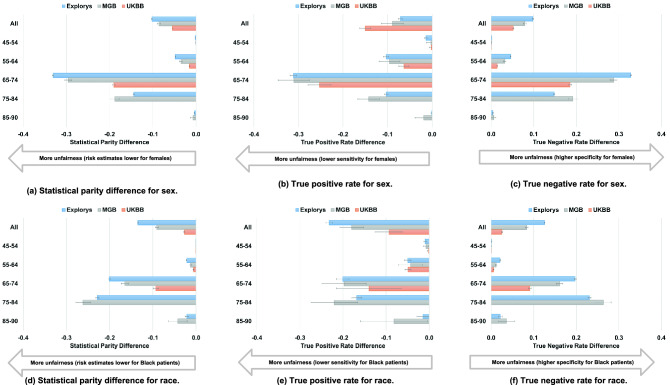
Fairness analysis for CHARGE-AF. Note that data was not available in the UKBB for the 75–84 and 85–90 age subpopulations.

As shown in Fig. 3A, even though sex is not included in CHARGE-AF, risk estimates using the CHARGE-AF model were much lower for females than for males, with regard to the population as a whole and particularly in the age groups 65–74 and 75–84; for example, the 65–74 years subgroup had a statistical parity difference of − 0.331 [95% CI − 0.333 to − 0.329] in Explorys. As shown in Fig. 3B, consistent across each dataset, sensitivity was lower for females, particularly in intermediate age groups (65–74 and 75–84); for example, the 65–74 years subgroup had a sensitivity difference of − 0.311 [95% CI − 0.319 to − 0.304] in Explorys. As shown in Fig. 3C, specificity was higher for females in intermediate age groups (65–74 and 75–84); for example, the 65–74 years subgroup had a specificity difference of 0.328 [95% CI 0.326–0.330] in Explorys.

Similar to the unfairness of pattens for sex, unfairness for race was notable in intermediate age groups (65–74 and 75–84). As shown in Fig. 3D, risk estimates using the CHARGE-AF model were much lower for Black individuals than for White individuals, as expected since White race is a risk enhancing factor in the CHARGE-AF model; for example, the 75–84 years subgroup had statistical parity difference of − 0.228 [95% CI − 0.232 to − 0.225] in Explorys. Likely as a result of systematically lower predicted risk estimates, CHARGE-AF exhibited lower sensitivity (Fig. 3E) and greater specificity (Fig. 3F) among Black individuals; as an example, sensitivity difference was − 0.168 [95% CI − 0.180 to − 0.157], and specificity difference was 0.231 [0.228–0.235] for the 75–84 years subgroup in Explorys. For both sex and race, behavior indicating unfairness was similar between Explorys and MGB but less prominent in the UKBB.

### Performance heterogeneity of PCE

As with CHARGE-AF, we observed that a variety of subgroups were affected by limited discrimination, limited calibration, or both (Supplementary Tables XII and XIII). Only a few of the subgroups across the 3 datasets were associated with both good discrimination and calibration (e.g., female-White 40–49 in the UKBB with a percentage of 21.9% of the total patients in this subgroup).

**Figure 4:**
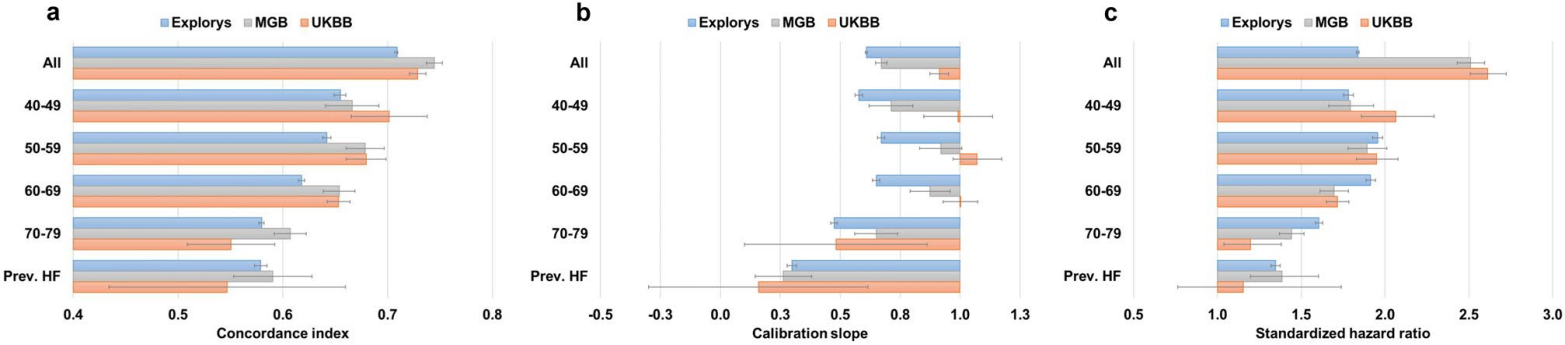
Performance measures for PCE (Female-White). Prev. = Prevalence; HF = Heart failure. Refer to Supplementary Table VIII for additional PCE models.

Consistent with CHARGE-AF, discrimination using the PCE decreased with older age from a concordance index of 0.655 [95% CI 0.649–0.660] for the 40–49 years subgroup to 0.580 [0.577–0.582] for the 70–79 years subgroup in Explorys (Fig. 4A). This behavior was consistent across all 3 datasets. Discrimination among individuals with prevalent HF was similar to the overall 70–79 years subgroup.

We also observed suboptimal calibration using the PCE within subgroups of age, with consistently lower calibration slopes in the youngest and oldest groups, indicating an overall tendency to overestimate risk at extremes of age (Fig. 4B); for example, in Explorys, values were the lowest for the 40–49 years subgroup with a slope of 0.577 [95% CI 0.561–0.594], and 0.474 [0.460–0.487] for the 70–79 years subgroup, in comparison to values above 0.7 for the intermediate age subgroups. Similar to CHARGE-AF, calibration performance was limited among individuals with prevalent HF, again with a general tendency to overestimate risk.

The strength of association between the PCE score on incident ASCVD (as measured using SHRs) was highest in intermediate age groups (50–59 and 60–69) compared to the younger (40–49) and older (70–79) age groups (Fig. 4C); for example, highest SHR was 1.956 [95% CI 1.927–1.985] for the 50–59 subgroup and 1.606 [1.585–1.628] for the 70–79 subgroup, in Explorys.

### Unfair behaviors for PCE

**Figure 5:**
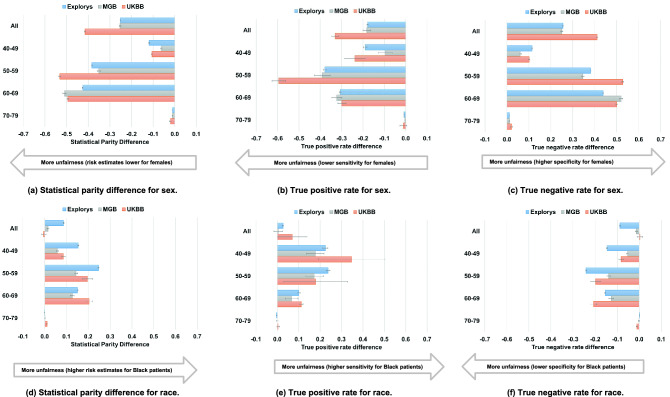
Fairness analysis for PCE.

As shown in Fig. 5A, risk estimates using the PCE were much lower for females than for males in the overall population as well as within the intermediate age groups (50–59 and 60–69); for example, in Explorys, the 60–69 years subgroup had a statistical parity difference of − 0.426 [95% CI − 0.427 to − 0.424]. As shown in Fig. 5B, across all datasets, sensitivity was lower for females, especially in intermediate age groups (50–59 and 60–69); for example, the 50–59 years subgroup had a sensitivity difference of − 0.379 [95% CI − 0.386 to − 0.373] in Explorys. Specificity was higher among females (Fig. 5C), especially in intermediate age groups (50–59 and 60–69); for example, the 60–69 years subgroup had a specificity difference of 0.438 [95% CI 0.436–0.439] in Explorys. Overall, patterns observed on the basis of sex using the PCE were similar to those observed using CHARGE-AF.

As shown in Fig. 5D, unlike CHARGE-AF, risk estimates using the PCE were higher in Black individuals in all datasets; this effect was especially noticeable in intermediate age groups (50–59 and 60–69); for example, statistical parity difference between the 50–59 years subgroup was the largest compared to the other subgroups in Explorys at 0.247 [95% CI 0.244–0.250]. In contrast to CHARGE-AF, greater risk estimates led to increased sensitivity among Black individuals versus White individuals (Fig. 5E); for example, sensitivity difference between the 40–49 years and 50–59 years subgroups were the largest compared to the other subgroups in Explorys at 0.224 [95% CI 0.211–0.237] and 0.237 [0.228–0.246], respectively. Differences in sensitivity on the basis of race decreased with increasing age in all 3 datasets, with very little difference observed in the oldest age group (70–79). As shown in Fig. 5F, across specific age ranges, specificity was lower for Black individuals than for White individuals; this effect was especially noticeable in intermediate age groups (50–59 and 60–69); for example, specificity difference between the 50–59 years subgroup was the greatest compared to the other subgroups in Explorys at − 0.241 [95% CI − 0.244 to − 0.239].

## Discussion

We analyzed three large independent datasets including millions of individuals and identified important patterns of performance heterogeneity across clinically relevant subgroups as indicated by standard performance measures including discrimination, calibration, SHRs, and fairness metrics. Our results build on previous efforts to understand estimation of AF and ASCVD risk in several key ways. First, we assessed the scores on very large databases, allowing us to quantify performance within granular subgroups. Second, we provide results applicable to 3 resources, allowing us to assess consistency in results across independent samples. Third, we perform analyses of two distinct outcomes, which allows for identification of potential patterns of heterogeneity that may be shared across risk estimators for different conditions. Fourth, our results highlight the magnitude of important limitations in performance affecting sizeable portions of the population, in particular patients at older ages and with prevalent conditions. Fifth, to our knowledge, our study is the first to report on fairness-related measures for the CHARGE-AF and PCE scores in relation to sex and race.

Patterns of variability were fairly consistent across the CHARGE-AF and PCE models. Importantly, we observed that discrimination and calibration were consistently worse at extremes of age, as well as for individuals with certain prevalent conditions (e.g., HF). Furthermore, we observed evidence of potentially unfair performance, with significant differences in fairness metrics for sex and race in both scores. For instance, the sensitivity difference of both scores was much lower for females than males in the intermediate-age subgroups, suggesting that current scores may miss more women at high risk for events, potentially worsening existing sex-related treatment gaps^38^. Overall, our findings underscore the importance of evaluating prognostic models across the many specific subpopulations in which risk prediction is intended, in order to better understand the accuracy and potential unfairness of the prognostic information used to drive clinical decisions at the point of care.

Our findings suggest that clinicians utilizing prognostic models should not assume that a given level of performance in the overall population will translate to similar accuracy within a subgroup of the population to which their patient belongs. Consistent with prior findings suggesting good overall performance of CHARGE-AF^17,18^ and the PCE^2,10^ across multiple populations, we observed moderate or greater discrimination using each score in our datasets. However, we observed that multiple standard metrics (e.g., discrimination and calibration) vary substantially within subpopulations. Specifically, we observed a consistent pattern of decreasing discrimination for higher age groups, a finding which may be attributable to less variability in event risk among older individuals. Furthermore, since assessing discrimination within a subgroup defined by a certain feature precludes classification of risk on the basis of that feature (i.e., discrimination is adjusted), stratification by variables with substantial effects on event risk will decrease discrimination. Similar to discrimination, we also observed increasing miscalibration in higher age groups, which may be related to greater average event risk. In addition to age, miscalibration related to baseline event risk may also be impacted by varying treatment patterns across different settings and over time. Ultimately, since the majority of incidents CVD occur among older individuals, more accurate models for an older population remains a critical unmet need. Future work is needed to assess whether models derived within specific subgroups of clinical importance may lead to better and more consistent model performance across important subsets of the population.

In addition to variation across standard model metrics, our findings also suggest that common prognostic models may have performance indicating unfairness across strata of sex and race. As discussed above, CHARGE-AF had lower sensitivity and greater specificity among women. A similar pattern was observed among Black individuals. Although use of the PCE also led to lower sensitivity and greater specificity among women, it demonstrated the opposite pattern (greater sensitivity and lower specificity) among Black individuals. It is notable that these differences exist despite the fact that the PCE has dedicated models specific to race and sex (i.e., there are 4 distinct equations). Since PCE model predictions were generally better calibrated among White individuals, our findings suggest that model derivation in populations having greater representation of women and Black individuals may lead to more accurate and generalizable models with less unfairness.

There are several potential strategies to mitigate the significant heterogeneity in performance we characterized and quantified in the current study. One strategy is to adjust models according to empirically observed patterns of unfairness, which has been previously proposed as a method to reduce unfairness and minimize overtreatment of healthy individuals^7,39^. Another approach is to reweight existing models^40–42^ within each subgroup of the population, resulting in distinct weights for each subgroup of interest. Yet another strategy is to create new higher capacity models that include additional (e.g., socioeconomic deprivation)^7,43^ or more precisely defined predictors (e.g., granular race definitions), which may offer more consistent prognostic value across subgroups. Any chosen strategy should consider both calibration and discrimination not only separately but also jointly; for example, even if a mitigation strategy could handle limited calibration performance in a certain subgroup, effects may not translate to other subgroups. Furthermore, certain strategies may result in a tradeoff in which one measure is improved (e.g., discrimination), while another is worsened (e.g., fairness-related).

Our study has several limitations. First, despite analysis of three large datasets, the majority of individuals included were White, limiting the precision of subgroup-based estimates in Black individuals. Second, since dedicated PCE models are available only for White and Black individuals, as performed previously^29^, the models for Black individuals were utilized for individuals identifying as Black, and the models for White individuals were utilized for individuals of all other races. Evidence suggests that cardiovascular risk and outcomes^5,29^ may differ importantly on account of more granular classification of race and ethnicity, and therefore we acknowledge that our race classification may have contributed to observed heterogeneity in PCE performance. We submit that future work is warranted to develop more accurate methods of risk ASCVD risk stratification in these populations. Third, we were unable to assess the effects of socioeconomic deprivation^44–46^ given the lack of available data in Explorys and MGB. Fourth, given that the CHARGE-AF and PCE scores did not model death as a competing risk, and death data are not available in the Explorys, we did not adjust for the competing risk of death (note that death rates within the windows of interest in the UKBB and MGB datasets were low). Fifth, as with any EHR-based study, misclassification of exposures and outcomes is possible. Additionally, cause of death data is available only in UKBB, and therefore fatal ASCVD events not resulting in hospitalization may have been missed in the EHR samples. To mitigate misclassification, we utilized previously published disease definitions and constructed our EHR samples to include individuals receiving longitudinal ambulatory care. Furthermore, predictive utility was similar to expectations for both scores in all 3 datasets compared to values observed from prior prospective cohort studies^12,15^. Sixth, we have not applied recently proposed fairness metrics that assess individual fairness (rather than assessment at the population level)^47,48^. Sixth, although our findings provide important evidence of performance heterogeneity and potential unfairness in commonly used risk estimators, we did not explore mitigation methods.

In summary, we evaluated the CHARGE-AF and the PCE scores in three independent datasets totaling over 5 million individuals, identifying important performance heterogeneity and unfairness. The patterns we observed were consistent, including worse discrimination of risk among older individuals and substantial miscalibration at extremes of age. We also observed that use of common score thresholds may lead to unfairness on the basis of sex and race, which may worsen existing treatment gaps. Overall, users of current clinical risk stratification methods should exercise caution when interpreting risk estimates obtained in certain subgroups (e.g., extremes of age), and there is a critical need to develop more robust risk estimators that display more consistent accuracy and fairness.

## Supplementary Information

Supplementary Information.

## Data Availability

The institutional review boards of Mass General Brigham (MGB) and IBM approved this study and its methods, including the EHR cohort assembly using the Explorys Dataset, data extraction, and analyses. MGB data contains potentially identifying information and may not be shared publicly. Explorys data can be made available through a commercial license (for details see: https://www.ibm.com/downloads/cas/4P0QB9JN). We are indebted to the UKBB and its participants who provided data for this analysis (UKBB Applications #7089 and #50658). All UKBB participants provided written informed consent. The UK Biobank was approved by the UK Biobank Research Ethics Committee (reference# 11/NW/0382). Source data are provided with this paper.

## Abbreviations

1 K PY: 1000 Patient years
AF: Atrial fibrillation
ASCVD: Atherosclerotic cardiovascular disease
CHARGE-AF: Cohorts for Heart and Aging Research in Genomic Epidemiology atrial fibrillation
CPT: Current Procedural Terminology
DBP: Diastolic blood pressure
EHR: Electronic health record
HDL: High-density lipoprotein
HF: Heart failure
ICD: International Classification of Diseases
Inc: Incidence
MGB: Mass General Brigham
MI: Myocardial infarction
PCE: Pooled Cohort Equations
SBP: Systolic blood pressure
SD: Standard deviation
SHR: Standardized hazard ratio
T2DM: Type 2 diabetes mellitus
TC: Total cholesterol
TIA: Transient ischemic attack
UKBB: United Kingdom Biobank

## References

[1] Damen JA, Pajouheshnia R, Heus P, Moons KGM, Reitsma JB, Scholten RJPM, Hooft L, Debray TPA (2019). Performance of the Framingham risk models and pooled cohort equations for predicting 10-year risk of cardiovascular disease: A systematic review and meta-analysis. BMC Med..

[2] Muntner P, Colantonio LD, Cushman M (2014). Validation of the atherosclerotic cardiovascular disease Pooled Cohort risk equations. JAMA.

[3] Kavousi M, Leening MJ, Nanchen D, Greenland P, Graham IM, Steyerberg EW, Ikram MA, Stricker BH, Hofman A, Franco OH (2014). Comparison of application of the ACC/AHA guidelines, Adult Treatment Panel III guidelines, and European Society of Cardiology guidelines for cardiovascular disease prevention in a European cohort. JAMA.

[4] DeFilippis AP, Young R, Carrubba CJ (2015). An analysis of calibration and discrimination among multiple cardiovascular risk scores in a modern multiethnic cohort. Ann. Intern. Med..

[5] Rana JS, Tabada GH, Solomon MD (2016). Accuracy of the atherosclerotic cardiovascular risk equation in a large contemporary, multiethnic population. J. Am. Coll. Cardiol..

[6] DeFilippis AP, Young R, McEvoy JW (2017). Risk score overestimation: The impact of individual cardiovascular risk factors and preventive therapies on the performance of the American Heart Association-American College of Cardiology-Atherosclerotic Cardiovascular Disease risk score in a modern multi-ethnic cohort. Eur. Heart J..

[7] Pylypchuk R, Wells S, Kerr A (2018). Cardiovascular disease risk prediction equations in 400,000 primary care patients in New Zealand: A derivation and validation study. Lancet.

[8] Lee CH, Woo YC, Lam JK, Fong CH, Cheung BM, Lam KS, Tan KC (2015). Validation of the Pooled Cohort equations in a long-term cohort study of Hong Kong Chinese. J. Clin. Lipidol..

[9] Jung KJ, Jang Y, Oh DJ, Oh BH, Lee SH, Park SW, Seung KB, Kim HK, Yun YD, Choi SH, Sung J, Lee TY, Kim SH, Koh SB, Kim MC, Chang Kim H, Kimm H, Nam C, Park S, Jee SH (2015). The ACC/AHA 2013 pooled cohort equations compared to a Korean Risk Prediction Model for atherosclerotic cardiovascular disease. Atherosclerosis.

[10] Khera R, Pandey A, Ayers CR, Carnethon MR, Greenland P, Ndumele CE, Nambi V, Seliger SL, Chaves PHM, Safford MM, Cushman M, Xanthakis V, Vasan RS, Mentz RJ, Correa A, Lloyd-Jones DM, Berry JD, de Lemos JA, Neeland IJ (2020). Performance of the Pooled Cohort Equations to estimate atherosclerotic cardiovascular disease risk by body mass index. JAMA Netw. Open..

[11] Nguyen QD, Odden MC, Peralta CA, Kim DH (2020). Predicting risk of atherosclerotic cardiovascular disease using Pooled Cohort Equations in older adults with frailty, multimorbidity, and competing risks. J. Am. Heart Assoc..

[12] Goff DC, Lloyd-Jones DM, Bennett G, Coady S, Dagostino RB, Gibbons R, Greenland P, Lackland DT, Levy D, Odonnell CJ, Robinson JG, Schwartz JS, Shero ST, Smith SC, Sorlie P, Stone NJ, Wilson PW, Jordan HS, Nevo L, Wnek J, Anderson JL, Halperin JL, Albert NM, Bozkurt B, Brindis RG, Curtis LH, DeMets D, Hochman JS, Kovacs RJ, Ohman EM, Pressler SJ, Sellke FW, Shen WK, Smith SC, Tomaselli GF, American College of Cardiology/American Heart Association Task Force on Practice Guidelines (2014). 2013 ACC/AHA guideline on the assessment of cardiovascular risk: A report of the American College of Cardiology/American Heart Association Task Force on Practice Guidelines. Circulation.

[13] Kemp Gudmundsdottir K, Fredriksson T, Svennberg E, Al-Khalili F, Friberg L, Frykman V, Hijazi Z, Rosenqvist M, Engdahl J (2020). Stepwise mass screening for atrial fibrillation using N-terminal B-type natriuretic peptide: The STROKESTOP II study. Europace.

[14] Khurshid S, Mars N, Haggerty CM, Huang Q, Weng LC, Hartzel DN, Lunetta KL, Ashburner JM, Anderson CD, Benjamin EJ, Salomaa V, Ellinor PT, Fornwalt BK, Ripatti S, Trinquart L, Lubitz SA, Regeneron Genetics Center (2021). Predictive accuracy of a clinical and genetic risk model for atrial fibrillation. Circ. Genom. Precis Med..

[15] Alonso A, Krijthe BP, Aspelund T, Stepas KA, Pencina MJ, Moser CB, Sinner MF, Sotoodehnia N, Fontes JD, Janssens AC, Kronmal RA, Magnani JW, Witteman JC, Chamberlain AM, Lubitz SA, Schnabel RB, Agarwal SK, McManus DD, Ellinor PT, Larson MG, Burke GL, Launer LJ, Hofman A, Levy D, Gottdiener JS, Kääb S, Couper D, Harris TB, Soliman EZ, Stricker BH, Gudnason V, Heckbert SR, Benjamin EJ (2013). Simple risk model predicts incidence of atrial fibrillation in a racially and geographically diverse population: The CHARGE-AF consortium. J. Am. Heart Assoc..

[16] Alonso, A., Roetker, N.S., Soliman, E.Z., Chen, L.Y., Greenland, P., Heckbert, S.R. Prediction of atrial fibrillation in a racially diverse cohort: The Multi-Ethnic Study of Atherosclerosis (MESA). *J. Am. Heart Assoc*. **5** (2016). 10.1161/JAHA.115.003077 PMC4802458 26908413

[17] Shulman E, Kargoli F, Aagaard P, Hoch E, Di Biase L, Fisher J, Gross J, Kim S, Krumerman A, Ferrick KJ (2016). Validation of the Framingham Heart Study and CHARGE-AF risk scores for atrial fibrillation in Hispanics, African–Americans, and Non-Hispanic Whites. Am. J. Cardiol..

[18] Christophersen IE, Yin X, Larson MG, Lubitz SA, Magnani JW, McManus DD, Ellinor PT, Benjamin EJ (2016). A comparison of the CHARGE-AF and the CHA_2_DS_2_-VASc risk scores for prediction of atrial fibrillation in the Framingham Heart Study. Am. Heart J..

[19] Khurshid S, Kartoun U, Ashburner JM, Trinquart L, Philippakis A, Khera AV, Ellinor PT, Ng K, Lubitz SA (2021). Performance of atrial fibrillation risk prediction models in over 4 million individuals. Circ. Arrhythm. Electrophysiol..

[20] FitzGerald C, Hurst S (2017). Implicit bias in healthcare professionals: A systematic review. BMC Med. Ethics..

[21] Kartoun U, Corey KE, Simon TG, Zheng H, Aggarwal R, Ng K, Shaw SY (2017). The MELD-Plus: A generalizable prediction risk score in cirrhosis. PLoS ONE.

[22] Dron JS, Wang M, Patel AP, Kartoun U, Ng K, Hegele RA, Khera AV (2021). Genetic predictor to identify individuals with high Lipoprotein(a) concentrations. Circ. Genom. Precis. Med..

[23] Committee on Strategies for Responsible Sharing of Clinical Trial Data; Board on Health Sciences Policy; Institute of Medicine. Sharing Clinical Trial Data: Maximizing Benefits, Minimizing Risk. Washington (DC): National Academies Press (US); 2015 Apr 20. Appendix B, Concepts and Methods for De-identifying Clinical Trial Data. https://www.ncbi.nlm.nih.gov/books/NBK285994/25590113

[24] Khurshid S, Reeder C, Harrington LX (2021). Cohort design and natural language processing to reduce bias in electronic health records research: The Community Care Cohort Project. medRxiv..

[25] Sudlow C, Gallacher J, Allen N (2015). UK Biobank: An open access resource for identifying the causes of a wide range of complex diseases of middle and old age. PLOS Med.

[26] UK Biobank. Integrating Electronic Health Records into the UK Biobank Resource. http://biobank.ctsu.ox.ac.uk/showcase/showcase/docs/DataLinkageProcess.pdf (2014).

[27] Hulme OL, Khurshid S, Weng L-C, Anderson CD, Wang EY, Ashburner JM, Ko D, McManus DD, Benjamin EJ, Ellinor PT, Trinquart L, Lubitz SA (2019). Development and validation of a prediction model for atrial fibrillation using electronic health records. JACC Clin. Electrophysiol..

[28] Patel AP, Wang M, Kartoun U, Ng K, Khera AV (2021). Quantifying and understanding the higher risk of atherosclerotic cardiovascular disease among South Asian individuals: Results from the UK Biobank prospective cohort study. Circulation.

[29] Rodriguez F, Chung S, Blum MR, Coulet A, Basu S, Palaniappan LP (2019). Atherosclerotic cardiovascular disease risk prediction in disaggregated Asian and Hispanic subgroups using electronic health records. J Am Heart Assoc..

[30] Stone NJ, Robinson JG, Lichtenstein AH, BaireyMerz CN, Blum CB, Eckel RH, Goldberg AC, Gordon D, Levy D, Lloyd-Jones DM, McBride P, Schwartz JS, Shero ST, Smith SC, Watson K, Wilson PW, American College of Cardiology/American Heart Association Task Force on Practice Guidelines (2014). 2013 ACC/AHA guideline on the treatment of blood cholesterol to reduce atherosclerotic cardiovascular risk in adults: A report of the American College of Cardiology/American Heart Association Task Force on Practice Guidelines. J. Am. Coll. Cardiol..

[31] Khurshid S, Keaney J, Ellinor PT, Lubitz SA (2016). A simple and portable algorithm for identifying atrial fibrillation in the electronic medical record. Am. J. Cardiol..

[32] Khurshid S, Choi SH, Weng LC, Wang EY, Trinquart L, Benjamin EJ, Ellinor PT, Lubitz SA (2018). Frequency of cardiac rhythm abnormalities in a half million adults. Circ. Arrhythm. Electrophysiol..

[33] Wang EY, Hulme OL, Khurshid S, Weng LC, Choi SH, Walkey AJ, Ashburner JM, McManus DD, Singer DE, Atlas SJ, Benjamin EJ, Ellinor PT, Trinquart L, Lubitz SA (2020). Initial precipitants and recurrence of atrial fibrillation. Circ. Arrhythm. Electrophysiol..

[34] Cox DR (1958). Two further applications of a model for binary regression. Biometrika.

[35] Bellamy, R., Dey, K., Hind, M., Hoffman, S.C., Houde, S., Kannan, K., Lohia, P., Martino, J., Mehta, S., Mojsilovic, A., Nagar, S., Ramamurthy, K., Richards, J.T., Saha, D., Sattigeri, P., Singh, M., Varshney, K., Zhang, Y. AI Fairness 360: An extensible toolkit for detecting, understanding, and mitigating unwanted algorithmic bias. ArXiv, abs/1810.01943. 2018.

[36] Himmelreich JCL, Lucassen WAM, Harskamp RE, Aussems C, van Weert HCPM, Nielen MMJ (2021). CHARGE-AF in a national routine primary care electronic health records database in the Netherlands: Validation for 5-year risk of atrial fibrillation and implications for patient selection in atrial fibrillation screening. Open Heart..

[37] R Core Team (2015). R: A language and environment for statistical computing. R Foundation for Statistical Computing Vienna, Austria. URL https://www.R-project.org/.

[38] Mehran R, Vogel B, Ortega R, Cooney R, Horton R (2019). The Lancet Commission on women and cardiovascular disease: Time for a shift in women's health. Lancet.

[39] Pennells L, Kaptoge S, Wood A, Sweeting M, Zhao X, White I, Burgess S, Willeit P, Bolton T, Moons KGM, van der Schouw YT, Selmer R, Khaw KT, Gudnason V, Assmann G, Amouyel P, Salomaa V, Kivimaki M, Nordestgaard BG, Blaha MJ, Kuller LH, Brenner H, Gillum RF, Meisinger C, Ford I, Knuiman MW, Rosengren A, Lawlor DA, Völzke H, Cooper C, Marín Ibañez A, Casiglia E, Kauhanen J, Cooper JA, Rodriguez B, Sundström J, Barrett-Connor E, Dankner R, Nietert PJ, Davidson KW, Wallace RB, Blazer DG, Björkelund C, Donfrancesco C, Krumholz HM, Nissinen A, Davis BR, Coady S, Whincup PH, Jørgensen T, Ducimetiere P, Trevisan M, Engström G, Crespo CJ, Meade TW, Visser M, Kromhout D, Kiechl S, Daimon M, Price JF, de la Cámara AG, Wouter Jukema J, Lamarche B, Onat A, Simons LA, Kavousi M, Ben-Shlomo Y, Gallacher J, Dekker JM, Arima H, Shara N, Tipping RW, Roussel R, Brunner EJ, Koenig W, Sakurai M, Pavlovic J, Gansevoort RT, Nagel D, Goldbourt U, Barr ELM, Palmieri L, Njølstad I, Sato S, Monique Verschuren WM, Varghese CV, Graham I, Onuma O, Greenland P, Woodward M, Ezzati M, Psaty BM, Sattar N, Jackson R, Ridker PM, Cook NR, D'Agostino RB, Thompson SG, Danesh J, Di Angelantonio E, Emerging Risk Factors Collaboration (2019). Equalization of four cardiovascular risk algorithms after systematic recalibration: Individual-participant meta-analysis of 86 prospective studies. Eur. Heart J..

[40] Park Y, Hu J, Singh M (2021). Comparison of methods to reduce bias from clinical prediction models of postpartum depression. JAMA Netw. Open..

[41] Calders, T., Kamiran, F., Pechenizkiy, M. Building classifiers with independency constraints. in *ICDM Workshops—IEEE International Conference on Data Mining*. 2009:13–8. August 6–9, 2009; Miami, Florida.

[42] Hainmueller J (2012). Entropy balancing for causal effects: A multivariate reweighting method to produce balanced samples in observational studies. Polit. Anal..

[43] Dalton JE, Perzynski AT, Zidar DA, Rothberg MB, Coulton CJ, Milinovich AT, Einstadter D, Karichu JK, Dawson NV (2017). Accuracy of cardiovascular risk prediction varies by neighborhood socioeconomic position: A retrospective cohort study. Ann. Intern. Med..

[44] Rajkomar A, Hardt M, Howell MD, Corrado G, Chin MH (2018). Ensuring fairness in machine learning to advance health equity. Ann. Intern. Med..

[45] Townsend P, Phillimore P, Beattie A (1988). Health and Deprivation: Inequality and The North.

[46] Foster HME, Celis-Morales CA, Nicholl BI, Petermann-Rocha F, Pell JP, Gill JMR, O’Donnell CA, Mair FS (2018). The effect of socioeconomic deprivation on the association between an extended measurement of unhealthy lifestyle factors and health outcomes: A prospective analysis of the UK Biobank cohort. Lancet Public Health..

[47] Yurochkin, M., Bowery, A., Sun, Y. Training individually fair ML models with sensitive subspace robustness. ICLR 2020.

[48] Maity, S., Xue, S., Yurochkin, M., Sun, Y. Statistical inference for individual fairness. ICLR 2021.

