# Supplementary material for: Prediction performance and fairness heterogeneity in cardiovascular risk models
Source: Sci Rep. 2022 Jul 22;12:12542. doi: 10.1038/s41598-022-16615-3 (PMC9307639; doi:10.1038/s41598-022-16615-3)
Supplement: Supplementary file 1 — Supplementary Information. [file 41598_2022_16615_MOESM1_ESM.docx]

**Supplementary Table I. Cohort selection in Explorys (incident AF)**

| **# patients** | **Selection criteria** |
| --- | --- |
| 21,809,334 | A predefined cohort with patients at a high likelihood for diseases such as T2DM and hypertension. |
| 11,515,265 | 2 office visits at least 2Y (consider the earliest office visit that occurs at least 2Y after a first office visit as baseline). |
| 8,110,115 | Earliest office visit that occurs at least 2Y after a first office visit and that also has all values of height, weight, SBP, and DBP (most recent values within the preceding 14 months to baseline). |
| 5,459,803 | Age ≥45 and ≤95 at baseline. |
| 5,147,261 | Exclude patients with prevalent AF. |
| 4,750,660 | Exclude patients with no follow-up  (i.e., baseline date is their last interaction). |

**Supplementary Table II. Cohort selection in Explorys (incident ASCVD)**

| **# patients** | **Selection criteria** |
| --- | --- |
| 21,853,866 | A predefined cohort of patients possibly with conditions such as T2DM and hypertension. |
| 11,536,447 | 2 office visits at least 2Y (consider the earliest office visit that occurs at least 2Y after a first office visit as baseline). |
| 5,604,894 | Earliest office visit that occurs at least 2Y after a first office visit and that also has all values of: SBP (most recent value within the preceding 14 months to baseline), TC and HDL (most recent value within the preceding 5 years to baseline). |
| 4,263,069 | Age ≥40 and ≤79 at baseline. |
| 4,259,932 | Exclude patients with a recent pregnancy (preceding 9M). |
| 3,866,202 | Exclude patients with prevalent ASCVD (ASCVD is defined as MI, Stroke/TIA, or both). |
| 3,656,680 | Exclude patients with no follow-up  (i.e., baseline date is their last interaction). |

**Supplementary Table III. Cohort selection in MGB (incident AF)**

| **# patients** | **Selection criteria** |
| --- | --- |
| 520,868 | Individuals in Community Care Cohort Project. |
| 358,487 | Exclude patients with a missing component of CHARGE-AF at baseline. |
| 343,632 | Exclude patients with prevalent AF. |
| 337,672 | Exclude patients with no follow-up (i.e., baseline date is their last interaction). |
| 174,644 | Age ≥46 and ≤95 at baseline. |

**Supplementary Table IV. Cohort selection in MGB (incident ASCVD)**

| **# patients** | **Selection criteria** |
| --- | --- |
| 520,868 | Individuals in Community Care Cohort Project. |
| 325,490 | Exclude patients with a missing component of PCE at baseline. |
| 307,483 | Exclude patients with prevalent ASCVD (ASCVD is defined as MI, Stroke/TIA, or both). |
| 302,072 | Exclude patients with no follow-up  (i.e., baseline date is their last interaction). |
| 198,184 | Age ≥40 and ≤79 at baseline. |

**Supplementary Table V. Cohort selection in the UK Biobank (incident AF)**

| **# patients** | **Selection criteria** |
| --- | --- |
| 502,521 | All registered patients. |
| 456,793 | Age ≥45 and ≤90 at baseline (“enroll date”). |
| 453,573 | Have all values of height, weight, SBP, and DBP at baseline. |
| 445,357 | No history of AF on or prior to baseline. |
| 445,329 | Exclude patients who asked to opt out from the study. |

**Supplementary Table VI. Cohort selection in the UK Biobank (incident ASCVD)**

| **# patients** | **Selection criteria** |
| --- | --- |
| 502,521 | All registered patients. |
| 424,043 | Exclude patients with no PCE score at baseline (“enroll date”). Note that the score was pre-calculated^45^. |
| 424,041 | Age ≥40 and ≤79 at baseline. |
| 408,215 | Exclude patients with history of ASCVD prior to baseline (ASCVD is defined as MI, Stroke/TIA, or both). |
| 408,171 | Exclude patients with an unknown race  (“Prefer not to answer”, “Do not know”). |
| 408,154 | Exclude patients who asked to opt out from the study. |

**Supplementary Table VII. Clinical factor definitions**

| **Phenotype** | **Code type** | **Data codes** | **Data code definitions** |
| --- | --- | --- | --- |
| Diabetes | ICD9 | 249, 249.0, 249.00, 249.01, 249.1, 249.10, 249.11, 249.2, 249.20, 249.21, 249.3, 249.30, 249.31, 249.4, 249.40, 249.41, 249.5, 249.50, 249.51, 249.6, 249.60, 249.61, 249.7, 249.70, 249.71, 249.8, 249.80, 249.81, 249.9, 249.90, 249.91, 250, 250.0, 250.00, 250.01, 250.02, 250.03, 250.1, 250.10, 250.11, 250.12, 250.13, 250.2, 250.20, 250.21, 250.22, 250.23, 250.3, 250.30, 250.31, 250.32, 250.33, 250.4, 250.40, 250.41, 250.42, 250.43, 250.5, 250.50, 250.51, 250.52, 250.53, 250.6, 250.60, 250.61, 250.62, 250.63, 250.7, 250.70, 250.71, 250.72, 250.73, 250.8, 250.80, 250.81, 250.82, 250.83, 250.9, 250.90, 250.91, 250.92, 250.93, 357.2, 362.01, 362.02, 362.03, 362.04, 362.05, 362.06, 362.07, 366.41, 791.6 | Secondary diabetes mellitus without mention of complication, not stated as uncontrolled, or unspecified, Secondary diabetes mellitus without mention of complication, uncontrolled, Secondary diabetes mellitus with ketoacidosis, not stated as uncontrolled, or unspecified, Secondary diabetes mellitus with ketoacidosis, uncontrolled, Secondary diabetes mellitus with hyperosmolarity, not stated as uncontrolled, or unspecified, Secondary diabetes mellitus with hyperosmolarity, uncontrolled, Secondary diabetes mellitus with other coma, not stated as uncontrolled, or unspecified, Secondary diabetes mellitus with other coma, uncontrolled, Secondary diabetes mellitus with renal manifestations, not stated as uncontrolled, or unspecified, Secondary diabetes mellitus with renal manifestations, uncontrolled, Secondary diabetes mellitus with ophthalmic manifestations, not stated as uncontrolled, or unspecified, Secondary diabetes mellitus with ophthalmic manifestations, uncontrolled, Secondary diabetes mellitus with neurological manifestations, not stated as uncontrolled, or unspecified, Secondary diabetes mellitus with neurological manifestations, uncontrolled, Secondary diabetes mellitus with peripheral circulatory disorders, not stated as uncontrolled, or unspecified, Secondary diabetes mellitus with peripheral circulatory disorders, uncontrolled, Secondary diabetes mellitus with other specified manifestations, not stated as uncontrolled, or unspecified, Secondary diabetes mellitus with other specified manifestations, uncontrolled, Secondary diabetes mellitus with unspecified complication, not stated as uncontrolled, or unspecified, Secondary diabetes mellitus with unspecified complication, uncontrolled, Diabetes mellitus without mention of complication, type II or unspecified type, not stated as uncontrolled, Diabetes mellitus without mention of complication, type I [juvenile type], not stated as uncontrolled, Diabetes mellitus without mention of complication, type II or unspecified type, uncontrolled, Diabetes mellitus without mention of complication, type I [juvenile type], uncontrolled, Diabetes with ketoacidosis, type II or unspecified type, not stated as uncontrolled, Diabetes with ketoacidosis, type I [juvenile type], not stated as uncontrolled, Diabetes with ketoacidosis, type II or unspecified type, uncontrolled, Diabetes with ketoacidosis, type I [juvenile type], uncontrolled, Diabetes with hyperosmolarity, type II or unspecified type, not stated as uncontrolled, Diabetes with hyperosmolarity, type I [juvenile type], not stated as uncontrolled, Diabetes with hyperosmolarity, type II or unspecified type, uncontrolled, Diabetes with hyperosmolarity, type I [juvenile type], uncontrolled, Diabetes with other coma, type II or unspecified type, not stated as uncontrolled, Diabetes with other coma, type I [juvenile type], not stated as uncontrolled, Diabetes with other coma, type II or unspecified type, uncontrolled, Diabetes with other coma, type I [juvenile type], uncontrolled, Diabetes with renal manifestations, type II or unspecified type, not stated as uncontrolled, Diabetes with renal manifestations, type I [juvenile type], not stated as uncontrolled, Diabetes with renal manifestations, type II or unspecified type, uncontrolled, Diabetes with renal manifestations, type I [juvenile type], uncontrolled, Diabetes with ophthalmic manifestations, type II or unspecified type, not stated as uncontrolled, Diabetes with ophthalmic manifestations, type I [juvenile type], not stated as uncontrolled, Diabetes with ophthalmic manifestations, type II or unspecified type, uncontrolled, Diabetes with ophthalmic manifestations, type I [juvenile type], uncontrolled, Diabetes with neurological manifestations, type II or unspecified type, not stated as uncontrolled, Diabetes with neurological manifestations, type I [juvenile type], not stated as uncontrolled, Diabetes with neurological manifestations, type II or unspecified type, uncontrolled, Diabetes with neurological manifestations, type I [juvenile type], uncontrolled, Diabetes with peripheral circulatory disorders, type II or unspecified type, not stated as uncontrolled, Diabetes with peripheral circulatory disorders, type I [juvenile type], not stated as uncontrolled, Diabetes with peripheral circulatory disorders, type II or unspecified type, uncontrolled, Diabetes with peripheral circulatory disorders, type I [juvenile type], uncontrolled, Diabetes with other specified manifestations, type II or unspecified type, not stated as uncontrolled, Diabetes with other specified manifestations, type I [juvenile type], not stated as uncontrolled, Diabetes with other specified manifestations, type II or unspecified type, uncontrolled, Diabetes with other specified manifestations, type I [juvenile type], uncontrolled, Diabetes with unspecified complication, type II or unspecified type, not stated as uncontrolled, Diabetes with unspecified complication, type I [juvenile type], not stated as uncontrolled, Diabetes with unspecified complication, type II or unspecified type, uncontrolled, Diabetes with unspecified complication, type I [juvenile type], uncontrolled, Polyneuropathy in diabetes, Background diabetic retinopathy, Proliferative diabetic retinopathy, Nonproliferative diabetic retinopathy NOS, Mild nonproliferative diabetic retinopathy, Moderate nonproliferative diabetic retinopathy, Severe nonproliferative diabetic retinopathy, Diabetic macular edema, Diabetic cataract, Acetonuria |
| Diabetes | ICD10 | E08.00, E08.01, E08.10, E08.11, E08.21, E08.22, E08.29, E08.311, E08.319, E08.321, E08.329, E08.331, E08.339, E08.341, E08.349, E08.351, E08.359, E08.36, E08.39, E08.40, E08.41, E08.42, E08.43, E08.44, E08.49, E08.51, E08.52, E08.610, E08.618, E08.620, E08.621, E08.622, E08.628, E08.630, E08.638, E08.641, E08.649, E08.65, E08.69, E08.8, E08.9, E08.90, E09.00, E09.01, E09.10, E09.11, E09.21, E09.22, E09.29, E09.311, E09.319, E09.321, E09.329, E09.331, E09.339, E09.341, E09.349, E09.351, E09.359, E09.36, E09.39, E09.40, E09.41, E09.42, E09.43, E09.44, E09.49, E09.51, E09.52, E09.59, E09.610, E09.618, E09.620, E09.621, E09.622, E09.628, E09.630, E09.638, E09.641, E09.649, E09.65, E09.69, E09.8, E09.9, E10.10, E10.11, E10.21, E10.22, E10.29, E10.311, E10.319, E10.321, E10.329, E10.331, E10.339, E10.341, E10.349, E10.351, E10.359, E10.36, E10.39, E10.40, E10.41, E10.42, E10.43, E10.44, E10.49, E10.51, E10.52, E10.59, E10.610, E10.618, E10.620, E10.621, E10.622, E10.628, E10.630, E10.638, E10.641, E10.649, E10.65, E10.69, E10.8, E10.9, E11.00, E11.01, E11.21, E11.22, E11.29, E11.311, E11.319, E11.321, E11.329, E11.331, E11.339, E11.341, E11.349, E11.351, E11.359, E11.36, E11.39, E11.40, E11.41, E11.42, E11.51, E11.52, E11.59, E11.610, E11.618, E11.620, E11.621, E11.622, E11.628, E11.630, E11.638, E11.641, E11.649, E11.65, E11.69, E11.8, E11.9, E13.00, E13.01, E13.10, E13.11, E13.21, E13.22, E13.29, E13.311, E13.319, E13.321, E13.329, E13.331, E13.339, E13.341, E13.349, E13.351, E13.359, E13.36, E13.39, E13.40, E13.41, E13.42, E13.43, E13.44, E13.49, E13.51, E13.52, E13.59, E13.610, E13.618, E13.620, E13.621, E13.622, E13.628, E13.630, E13.638, E13.641, E13.649, E13.65, E13.69, E13.8, E13.9, R82.4 | Diabetes Mellitus Due To Underlying Condition With Hyperosmolarity without nonketotic hyperglycemic-hyperosmolar coma (NKHHC), Diabetes Mellitus Due To Underlying Condition With Hyperosmolarity With Coma, Diabetes Mellitus Due To Underlying Condition With Ketoacidosis Without Coma, Diabetes Mellitus Due To Underlying Condition With Ketoacidosis With Coma, Diabetes Mellitus Due To Underlying Condition With Diabetic Nephropathy, Diabetes Mellitus Due To Underlying Condition With Diabetic chronic kidney disease, Diabetes Mellitus Due to underlying condition with other diabetic kidney complication, Diabetes Mellitus Due To Underlying Condition With Unspecified Diabetic Retinopathy With Macular Edema, Diabetes Mellitus Due To Underlying Condition With Unspecified Diabetic Retinopathy Without Macular Edema, Diabetes Mellitus due to underlying condition with mild nonproliferative diabetic retinopathy with macular edema, Diabetes mellitus due to underlying condition with mild nonproliferative diabetic retinopathy without macular edema, Diabetes mellitus due to underlying condition with moderate nonproliferative diabetic retinopathy with macular edema, Diabetes mellitus due to underlying condition with moderate nonproliferative diabetic retinopathy without macular edema, Diabetes mellitus due to underlying condition with severe nonproliferative diabetic retinopathy with macular edema, Diabetes mellitus due to underlying condition with severe nonproliferative diabetic retinopathy without macular edema, Diabetes mellitus due to underlying condition with proliferative diabetic retinopathy with macular edema, Diabetes mellitus due to underlying condition with proliferative diabetic retinopathy without macular edema, Diabetes Mellitus Due To Underlying Condition With Diabetic Cataract, Diabetes Mellitus Due To Underlying Condition With Other Diabetic Ophthalmic Complication, Diabetes Mellitus Due To Underlying Condition With Diabetic Neuropathy, Unspecified, Diabetes Mellitus Due To Underlying Condition With Diabetic Mononeuropathy, Diabetes Mellitus Due To Underlying Condition With Diabetic Polyneuropathy, Diabetes Mellitus Due To Underlying Condition With Diabetic Autonomic (Poly)Neuropathy, Diabetes Mellitus Due To Underlying Condition With Diabetic Amyotrophy, Diabetes Mellitus Due To Underlying Condition With Other Diabetic Neurological Complication, Diabetes Mellitus Due To Underlying Condition With Diabetic Peripheral Angiopathy Without Gangrene, Diabetes Mellitus Due To Underlying Condition With Diabetic Neuropathic Arthropathy, Diabetes Mellitus Due To Underlying Condition with diabetic neuropathic arthropathy, Diabetes Mellitus Due To Underlying Condition With Diabetic arthropathy, Diabetes Mellitus Due To Underlying Condition With diabetic dermatitis, Diabetes Mellitus Due To Underlying Condition With Foot Ulcer, Diabetes Mellitus Due To Underlying Condition With Other Skin ulcer, Diabetes Mellitus Due To Underlying Condition With other skin complications, Diabetes Mellitus Due To Underlying Condition With periodontal disease, Diabetes Mellitus Due To Underlying Condition With other oral complications, Diabetes Mellitus Due To Underlying Condition With hypoglycemia with coma, Diabetes mellitus due to underlying condition with hypoglycemia without coma, Diabetes Mellitus Due To Underlying Condition With Hyperglycemia, Diabetes Mellitus Due To Underlying Condition With Other Specified Complication, Diabetes Mellitus Due To Underlying Condition With Unspecified Complications, Diabetes mellitus due to underlying condition without Complications, Diabetes Mellitus Due To Underlying Condition Without Complications, Drug or Chemical induced diabetes mellitus with hyperosmolarity without nonketotic hyperglycemic-hyperosmolar coma (NKHHC), Drug Or Chemical Induced Diabetes Mellitus With Hyperosmolarity With Coma, Drug Or Chemical Induced Diabetes Mellitus With Ketoacidosis Without Coma, Drug Or Chemical Induced Diabetes Mellitus With Ketoacidosis With Coma, Drug Or Chemical Induced Diabetes Mellitus With Diabetic Nephropathy, Drug Or Chemical Induced Diabetes Mellitus With diabetic chronic kidney disease, Drug or chemical induced diabetes mellitus with other diabetic kidney complication, Drug Or Chemical Induced Diabetes Mellitus With Unspecified Diabetic Retinopathy With Macular Edema, Drug Or Chemical Induced Diabetes Mellitus With Unspecified Diabetic Retinopathy Without Macular Edema, Drug or chemical induced diabetes mellitus with mild nonproliferative diabetic retinopathy with macular edema, Drug or chemical induced diabetes mellitus with mild nonproliferative diabetic retinopathy without macular edema, Drug or chemical induced diabetes mellitus with moderate nonproliferative diabetic retinopathy with macular edema, Drug or chemical induced diabetes mellitus with moderate nonproliferative diabetic retinopathy without macular edema, Drug or chemical induced diabetes mellitus with severe nonproliferative diabetic retinopathy with macular edema, Drug or chemical induced diabetes mellitus with severe nonproliferative diabetic retinopathy without macular edema, Drug or chemical induced diabetes mellitus with proliferative diabetic retinopathy with macular edema, Drug or chemical induced diabetes mellitus with proliferative diabetic retinopathy without macular edema, Drug Or Chemical Induced Diabetes Mellitus With Diabetic Cataract, Drug Or Chemical Induced Diabetes Mellitus With Other Diabetic Ophthalmic Complication, Drug Or Chemical Induced Diabetes Mellitus With Neurological Complications With Diabetic Neuropathy, Unspecified, Drug Or Chemical Induced Diabetes Mellitus With Neurological Complications With Diabetic Mononeuropathy, Drug Or Chemical Induced Diabetes Mellitus With Neurological Complications With Diabetic Polyneuropathy, Drug Or Chemical Induced Diabetes Mellitus With Neurological Complications With Diabetic Autonomic (Poly)Neuropathy, Drug Or Chemical Induced Diabetes Mellitus With Neurological Complications With Diabetic Amyotrophy, Drug Or Chemical Induced Diabetes Mellitus With Neurological Complications With Other Diabetic Neurological Complication, Drug Or Chemical Induced Diabetes Mellitus With Diabetic Peripheral Angiopathy Without Gangrene, Drug Or Chemical Induced Diabetes Mellitus With Diabetic peripheral angiopathy with gangrene, Drug or chemical induced diabetes mellitus with other circulatory complications, Drug Or Chemical Induced Diabetes Mellitus With Diabetic neuropathic Arthropathy, Drug or chemical induced diabetes mellitus with other diabetic arthropathy, Drug Or Chemical Induced Diabetes Mellitus With diabetic dermatitis, Drug Or Chemical Induced Diabetes Mellitus With foot ulcer, Drug Or Chemical Induced Diabetes Mellitus With Other Skin ulcer, Drug Or Chemical Induced Diabetes Mellitus With other skin complications, Drug Or Chemical Induced Diabetes Mellitus With periodontal disease, Drug Or Chemical Induced Diabetes Mellitus With other oral complications, Drug Or Chemical Induced Diabetes Mellitus With Hypoglycemia with coma, Drug Or Chemical Induced Diabetes Mellitus With hypoglycemia without coma, Drug Or Chemical Induced Diabetes Mellitus With Hyperglycemia, Drug Or Chemical Induced Diabetes Mellitus With Other Specified Complication, Drug Or Chemical Induced Diabetes Mellitus With Unspecified Complications, Drug Or Chemical Induced Diabetes Mellitus without complications, Type 1 Diabetes Mellitus With Ketoacidosis Without Coma, Type 1 Diabetes Mellitus With Ketoacidosis With Coma, Type 1 Diabetes Mellitus With Diabetic Nephropathy, Type 1 Diabetes Mellitus With diabetic chronic kidney disease, Type 1 Diabetes Mellitus With other diabetic kidney complication, Type 1 Diabetes Mellitus With Unspecified Diabetic Retinopathy With Macular Edema, Type 1 Diabetes Mellitus With Unspecified Diabetic Retinopathy Without Macular Edema, Type 1 Diabetes mellitus with mild nonproliferative diabetic retinopathy with macular edema, Type 1 Diabetes mellitus with mild nonproliferative diabetic retinopathy without macular edema, Type 1 diabetes mellitus with moderate nonproliferative diabetic retinopathy with macular edema, Type 1 diabetes mellitus with moderate nonproliferative diabetic retinopathy without macular edema, Type 1 diabetes mellitus with severe nonproliferative diabetic retinopathy with macular edema, Type 1 diabetes mellitus with severe nonproliferative diabetic retinopathy without macular edema, Type 1 diabetes mellitus with proliferative diabetic retinopathy with macular edema, Type 1 diabetes mellitus with proliferative diabetic retinopathy without macular edema, Type 1 Diabetes Mellitus With Diabetic Cataract, Type 1 Diabetes Mellitus With Other Diabetic Ophthalmic Complication, Type 1 Diabetes Mellitus With Diabetic Neuropathy, Unspecified, Type 1 Diabetes Mellitus With diabetic mononeuropathy, Type 1 Diabetes Mellitus With Diabetic polyneuropathy, Type 1 Diabetes mellitus with diabetic autonomic (poly)neuropathy, Type 1 diabetes mellitus with diabetic amyotrophy, Type 1 diabetes mellitus with other diabetic neurological complication, Type 1 Diabetes Mellitus With Diabetic Peripheral Angiopathy Without Gangrene, Type 1 diabetes mellitus with diabetic peripheral angiopathy with gangrene, Type 1 diabetes mellitus with other circulatory complications, Type 1 diabetes mellitus with diabetic neuropathic arthropathy, Type 1 Diabetes Mellitus With Diabetic arthropathy, Type 1 Diabetes Mellitus With diabetic dermatitis, Type 1 Diabetes Mellitus With Other foot ulcer, Type 1 Diabetes Mellitus With Other Skin ulcer, Type 1 Diabetes Mellitus With other skin complications, Type 1 Diabetes Mellitus With periodontal disease, Type 1 Diabetes Mellitus With other oral complications, Type 1 Diabetes Mellitus With Hypoglycemia With Coma, Type 1 Diabetes Mellitus With Hypoglycemia without coma, Type 1 Diabetes Mellitus With Hyperglycemia, Type 1 Diabetes Mellitus With Other Specified Complication, Type 1 Diabetes Mellitus With Unspecified Complications, Type 1 Diabetes Mellitus Without Complications, Type 2 Diabetes Mellitus With Hyperosmolarity Without Nonketotic Hyperglycemic-Hyperosmolar Coma (Nkhhc), Type 2 Diabetes Mellitus With Hyperosmolarity With Coma, Type 2 diabetes mellitus with diabetic nephropathy, Type 2 diabetes mellitus with diabetic chronic kidney disease, Type 2 diabetes mellitus with other diabetic kidney complication, Type 2 Diabetes Mellitus With Unspecified Diabetic Retinopathy With Macular Edema, Type 2 Diabetes Mellitus With Unspecified Diabetic Retinopathy Without Macular Edema, Type 2 Diabetes Mellitus With Mild Nonproliferative Diabetic Retinopathy With Macular Edema, Type 2 Diabetes Mellitus With Mild Nonproliferative Diabetic Retinopathy Without Macular Edema, Type 2 Diabetes Mellitus With Moderate Nonproliferative Diabetic Retinopathy With Macular Edema, Type 2 Diabetes Mellitus with moderate nonproliferative diabetic retinopathy Without Macular Edema, Type 2 Diabetes Mellitus With Severe Nonproliferative Diabetic Retinopathy With Macular Edema, Type 2 Diabetes Mellitus With severe nonproliferative Diabetic Retinopathy Without Macular Edema, Type 2 Diabetes Mellitus with proliferative diabetic retinopathy with macular edema, Type 2 Diabetes Mellitus With proliferative diabetic retinopathy without macular edema, Type 2 Diabetes Mellitus With Diabetic Cataract, Type 2 Diabetes Mellitus With Other Diabetic Ophthalmic Complication, Type 2 Diabetes Mellitus With Diabetic Neuropathy, Unspecified, Type 2 Diabetes Mellitus With Diabetic mononeuropathy, Type 2 Diabetes Mellitus with diabetic polyneuropathy, Type 2 Diabetes Mellitus With Diabetic Peripheral Angiopathy Without Gangrene, Type 2 Diabetes Mellitus With diabetic peripheral Angiopathy With Gangrene, Type 2 Diabetes Mellitus with other circulatory complications, Type 2 diabetes mellitus with diabetic neuropathic arthropathy, Type 2 diabetes mellitus with other diabetic arthropathy, Type 2 Diabetes Mellitus with diabetic dermatitis, Type 2 Diabetes Mellitus With foot ulcer, Type 2 Diabetes Mellitus With Other Skin ulcer, Type 2 Diabetes Mellitus With other skin complications, Type 2 Diabetes Mellitus With periodontal disease, Type 2 Diabetes Mellitus With other oral complications, Type 2 Diabetes Mellitus With Hypoglycemia With Coma, Type 2 Diabetes Mellitus With Hypoglycemia without coma, Type 2 Diabetes Mellitus With Hyperglycemia, Type 2 Diabetes Mellitus With Other Specified Complication, Type 2 Diabetes Mellitus With Unspecified Complications, Type 2 Diabetes Mellitus Without Complications, Other specified diabetes mellitus with hyperosmolarity without nonketotic hyperglycemic-hyperosmolar coma (NKHHC), Other specified diabetes mellitus with hyperosmolarity with coma, Other specified diabetes mellitus with ketoacidosis without coma, Other specified diabetes mellitus with ketoacidosis with coma, Other specified diabetes mellitus with diabetic nephropathy, Other specified diabetes mellitus with diabetic chronic kidney disease, Other specified diabetes mellitus with other diabetic kidney complication, Other specified diabetes mellitus with unspecified diabetic retinopathy with macular edema, Other specified diabetes mellitus with unspecified diabetic retinopathy without macular edema, Other specified diabetes mellitus with mild nonproliferative diabetic retinopathy with macular edema, Other specified diabetes mellitus with mild nonproliferative diabetic retinopathy without macular edema, Other specified diabetes mellitus with moderate nonproliferative diabetic retinopathy with macular edema, Other specified diabetes mellitus with moderate nonproliferative diabetic retinopathy without macular edema, Other specified diabetes mellitus with severe nonproliferative diabetic retinopathy with macular edema, Other specified diabetes mellitus with severe nonproliferative diabetic retinopathy without macular edema, Other specified diabetes mellitus with proliferative diabetic retinopathy with macular edema, Other specified diabetes mellitus with proliferative diabetic retinopathy without macular edema, Other Specified Diabetes Mellitus With diabetic cataract, Other Specified Diabetes Mellitus With other diabetic ophthalmic complication, Other Specified Diabetes Mellitus With Diabetic neuropathy, unspecified, Other Specified Diabetes Mellitus With Diabetic mononeuropathy, Other Specified Diabetes Mellitus With Diabetic Polyneuropathy, Other Specified Diabetes Mellitus With Diabetic Autonomic (Poly)Neuropathy, Other Specified Diabetes Mellitus With Diabetic Amyotrophy, Other Specified Diabetes Mellitus With Other Diabetic Neurological Complication, Other specified diabetes mellitus with diabetic peripheral angiopathy without gangrene, Other specified diabetes mellitus with diabetic peripheral angiopathy with gangrene, Other Specified Diabetes Mellitus With Other Circulatory Complications, Other specified diabetes mellitus with diabetic neuropathic arthropathy, Other specified diabetes mellitus with other diabetic arthropathy, Other Specified Diabetes Mellitus With Diabetic Dermatitis, Other Specified Diabetes Mellitus With Foot Ulcer, Other Specified Diabetes Mellitus With Other Skin Ulcer, Other specified diabetes mellitus with other skin complications, Other specified diabetes mellitus with periodontal disease, Other specified diabetes mellitus with other oral complications, Other Specified Diabetes Mellitus With Hypoglycemia With Coma, Other Specified Diabetes Mellitus With Hypoglycemia Without Coma, Other Specified Diabetes Mellitus With Hyperglycemia, Other Specified Diabetes Mellitus With Other Specified Complication, Other Specified Diabetes Mellitus With Unspecified Complications, Other Specified Diabetes Mellitus Without Complications, Acetonuria |
| Diabetes | Med | metformin, glucophage, riomet, fortamet, glumetzia, chlorpropamide, glimepiride, glyburide, glipizide, tolazamide, tolbutamide, diabinese, amaryl, diabeta, micronase, glucotrol, glynase, tolinase, orinase, tolbutamide, repaglinide, nateglinide, prandin, starlix, pioglitazone, rosiglitazone, actos, avandia, sitagliptin, saxagliptin, linagliptin, alogliptin, januvia, onglyza, tradjenta, nesina, acarbose, miglitol, precose, glyset, pramlintide, symlin, liraglutide, exenatide, albiglutide, dulaglutide, victoza, bydureon, byetta, tanzeum, trulicity, canagliflozin, dapagliflozin, empagliflozin, invokana, farxiga, jardiance, actoplus, glucovance, metaglip, janumet, kombiglyze, prandimet, duetact, kazano, invokamet, xigduo, synjardy, jentadueto, avandamet, oseni, glyxambi, avandaryl, juvisync, glargine, basaglar, lantus, toujeo, detemir, degludec, levemir, tresiba | Biguanides, Sulfonylureas, Meglitinides, Thiazolidinediones, DPP-4 Inhibitors, Alpha-glucosidase inhibitors, Amylin analogue, GLP-1 Agonist, SGLT2 Inhibitor, Combination pills, long-actin insulin |
| Hyperlipidemia | ICD9 | 272, 272.0, 272.1, 272.2, 272.3, 272.4, 272.5, 272.6, 272.7, 272.8, 272.9, 759.9 | Pure hypercholesterolemia, Pure hyperglyceridemia, Mixed hyperlipidemia, Hyperchylomicronemia, Other hyperlipidemia, Hyperlipidemia, unspecified, Lipoprotein deficiency, Lipidoses, Other specified metabolic disorders, Disorder of lipoprotein metabolism, unspecified, Disorders of bile acid and cholesterol metabolism |
| Hyperlipidemia | ICD10 | E71.30, E75.21, E75.22, E75.5, E75.6, E77.0, E77.1, E78.0, E78.1, E78.2, E78.3, E78.4, E78.5, E78.6, E78.7, E78.70, E78.79, E78.81, E78.89, E78.9, E88.1, E88.89 | Disorder of fatty-acid metabolism, unspecified, Lipidoses, Lipidoses, Other lipid storage disorders, Lipid storage disorder, unspecified, Lipidoses, Lipidoses, Pure hypercholesterolemia, Pure hyperglyceridemia, Mixed hyperlipidemia, Hyperchylomicronemia, Other hyperlipidemia, Hyperlipidemia, unspecified, Lipoprotein deficiency, Disorders of bile acid and cholesterol metabolism, Disorder of bile acid and cholesterol metabolism, unspecified, Other disorders of bile acid and cholesterol metabolism, Lipid dermatoarthritis, Other lipoprotein metabolism disorders, Disorder of lipoprotein metabolism, unspecified, Lipodystrophy, Other specified metabolic disorders |
| Hyperlipidemia | Med | advicor, alirocumab, altoprev, antara, atorvastatin, cholestyramine, colesevelam, colestid, colestipol, crestor, evolocumab, ezetimibe, fenofibrate, fenofibric acid, fluvastatin, gemfibrozil, juxtapid, kynamro, lescol, lipitor, livalo, lomitapide, lopid, lovastatin, mipomersen, niacin, niacor, niaspan, nicotinic acid, pitavastatin, praluent, pravastatin, prevachol, prevalite, repatha, rosuvastatin, simvastatin, tricor, triglide, trilipix, vytorin, welchol, zetia, zocor |  |
| Heart Failure | ICD9 | 398.91, 402.01, 402.11, 402.91, 404.01, 404.03, 404.11, 404.13, 404.91, 404.93, 428, 428.1, 428.2, 428.21, 428.22, 428.23, 428.3, 428.31, 428.32, 428.33, 428.4, 428.41, 428.42, 428.43, 428.9 | Rheumatic heart failure (congestive), Malignant hypertensive heart disease with heart failure, Benign hypertensive heart disease with heart failure, Unspecified hypertensive heart disease with heart failure , Hypertensive heart and chronic kidney disease, malignant, with heart failure and with chronic kidney disease stage I through stage IV, or unspecified, Hypertensive heart and chronic kidney disease, malignant, with heart failure and with chronic kidney disease stage V or end stage renal disease, Hypertensive heart and chronic kidney disease, benign, with heart failure and with chronic kidney disease stage I through stage IV, or unspecified, Hypertensive heart and chronic kidney disease, benign, with heart failure and chronic kidney disease stage V or end stage renal disease, Hypertensive heart and chronic kidney disease, unspecified, with heart failure and with chronic kidney disease stage I through stage IV, or unspecified, Hypertensive heart and chronic kidney disease, unspecified, with heart failure and chronic kidney disease stage V or end stage renal disease, Congestive heart failure, unspecified, Left heart failure, Systolic heart failure, unspecified, Acute systolic heart failure, Chronic systolic heart failure, Acute on chronic systolic heart failure, Diastolic heart failure, unspecified, Acute diastolic heart failure, Chronic diastolic heart failure, Acute on chronic diastolic heart failure, Combined systolic and diastolic heart failure, unspecified, Acute combined systolic and diastolic heart failure, Chronic combined systolic and diastolic heart failure, Acute on chronic combined systolic and diastolic heart failure, Heart failure, unspecified |
| Heart Failure | ICD10 | I09.81, I11.0, I13.0, I13.2, I50.1, I50.20, I50.21, I50.22, I50.23, I50.30, I50.31, I50.32, I50.33, I50.40, I50.41, I50.42, I50.43, I50.9, I97.130, I97.131 | Rheumatic Heart Failure, Hypertensive Heart Disease With Heart Failure, Hypertensive Heart And Chronic Kidney Disease With Heart Failure And Stage 1 Through Stage 4 Chronic Kidney Disease, Or Unspecified Chronic Kidney Disease, Hypertensive Heart And Chronic Kidney Disease With Heart Failure And With Stage 5 Chronic Kidney Disease, Or End Stage Renal Disease, Left Ventricular Failure, Unspecified Systolic (Congestive) Heart Failure, Acute Systolic (Congestive) Heart Failure, Chronic Systolic (Congestive) Heart Failure, Acute On Chronic Systolic (Congestive) Heart Failure, Unspecified Diastolic (Congestive) Heart Failure, Acute Diastolic (Congestive) Heart Failure, Chronic Diastolic (Congestive) Heart Failure, Acute On Chronic Diastolic (Congestive) Heart Failure, Unspecified Combined Systolic (Congestive) And Diastolic (Congestive) Heart Failure, Acute Combined Systolic (Congestive) And Diastolic (Congestive) Heart Failure, Chronic Combined Systolic (Congestive) And Diastolic (Congestive) Heart Failure, Acute On Chronic Combined Systolic (Congestive) And Diastolic (Congestive) Heart Failure, Heart Failure, Unspecified, Postprocedural heart failure following cardiac surgery, Postprocedural heart failure following other surgery |
| Coronary Heart Disease | ICD9 | 411, 411.0, 411.1, 411.8, 411.81, 411.89, 413, 413.9, 414, 414.01, 414.02, 414.03, 414.04, 414.05, 414.06, 414.07, 414.1, 414.11, 414.19, 414.2, 414.3, 414.4, 414.8, 414.9, 429.2, 996.03, V45.81, V45.82 | Postmyocardial infarction syndrome, Intermediate coronary syndrome, Acute coronary occlusion without myocardial infarction, Other acute and subacute forms of ischemic heart disease, Other acute and subacute forms of ischemic heart disease, other, Angina decubitus, Other and unspecified angina pectoris, Coronary atherosclerosis of unspecified type of vessel, native or graft, Coronary atherosclerosis of native coronary artery, Coronary atherosclerosis of autologous vein bypass graft, Coronary atherosclerosis of nonautologous biological bypass graft, Coronary atherosclerosis of artery bypass graft, Coronary atherosclerosis of unspecified bypass graft, Coronary atherosclerosis of native coronary artery of transplanted heart, Coronary atherosclerosis of bypass graft (artery) (vein) of transplanted heart, Aneurysm of heart (wall), Aneurysm of coronary vessels, Chronic ischemic heart disease, unspecified, Chronic total occlusion of coronary artery, Coronary atherosclerosis due to lipid rich plaque, Coronary atherosclerosis due to calcified coronary lesion, Other specified forms of chronic ischemic heart disease, Chronic ischemic heart disease, unspecified, Cardiovascular disease, unspecified, Mechanical complication due to coronary bypass graft, Aortocoronary bypass status, Percutaneous transluminal coronary angioplasty status |
| Coronary Heart Disease | ICD10 | I20.0, I20.1, I20.8, I20.9, I21.A1, I21.A9, I21.01, I21.02, I21.09, I21.11, I21.19, I21.21, I21.29, I21.3, I21.4, I21.9, I22.0, I22.1, I22.2, I22.8, I22.9, I23.0, I23.1, I23.2, I23.3, I23.4, I23.5, I23.6, I23.7, I23.8, I24.0, I24.8, I24.9, I25.10, I25.110, I25.111, I25.118, I25.119, I25.3, I25.41, I25.42, I25.6, I25.700, I25.701, I25.708, I25.709, I25.710, I25.711, I25.718, I25.719, I25.720, I25.721, I25.728, I25.729, I25.730, I25.731, I25.738, I25.739, I25.790, I25.791, I25.798, I25.799, I25.810, I25.811, I25.812, I25.82, I25.83, I25.84, I25.89, I25.9, Z95.1, Z98.61 | Unstable angina, Angina pectoris with documented spasm, Other forms of angina pectoris, Angina pectoris, unspecified, Myocardial infarction type 2, Other myocardial infarction type, ST elevation (STEMI) myocardial infarction involving left main coronary artery, ST elevation (STEMI) myocardial infarction involving left anterior descending coronary artery, ST elevation (STEMI) myocardial infarction involving other coronary artery of anterior wall, ST elevation (STEMI) myocardial infarction involving right coronary artery, ST elevation (STEMI) myocardial infarction involving other coronary artery of inferior wall, ST elevation (STEMI) myocardial infarction involving left circumflex coronary artery, ST elevation (STEMI) myocardial infarction involving other sites, ST elevation (STEMI) myocardial infarction of unspecified site, Acute myocardial infarction, unspecified, Non-ST elevation (NSTEMI) myocardial infarction , Subsequent ST elevation (STEMI) myocardial infarction of anterior wall, Subsequent ST elevation (STEMI) myocardial infarction of inferior wall, Subsequent non-ST elevation (NSTEMI) myocardial infarction , Subsequent ST elevation (STEMI) myocardial infarction of other sites, Subsequent ST elevation (STEMI) myocardial infarction of unspecified site, Hemopericardium as current complication following acute myocardial infarction, Atrial septal defect as current complication following acute myocardial infarction, Ventricular septal defect as current complication following acute myocardial infarction, Rupture of cardiac wall without hemopericardium as current complication following acute myocardial infarction, Rupture of chordae tendineae as current complication following acute myocardial infarction, Rupture of papillary muscle as current complication following acute myocardial infarction, Thrombosis of atrium, auricular appendage, and ventricle as current complications following acute myocardial infarction, Postinfarction angina, Other current complications following acute myocardial infarction, Acute coronary thrombosis not resulting in myocardial infarction, Other forms of acute ischemic heart disease, Atherosclerotic heart disease of native coronary artery without angina pectoris, Atherosclerotic heart disease of native coronary artery with unstable angina pectoris, Atherosclerotic heart disease of native coronary artery with angina pectoris with documented spasm, Atherosclerotic heart disease of native coronary artery with other forms of angina pectoris, Atherosclerotic heart disease of native coronary artery with unspecified angina pectoris, Aneurysm of heart, Coronary artery aneurysm, Coronary artery dissection, Silent myocardial ischemia, Atherosclerosis of coronary artery bypass graft(s), unspecified, with unstable angina pectoris, Atherosclerosis of coronary artery bypass graft(s), unspecified, with angina pectoris with documented spasm, Atherosclerosis of coronary artery bypass graft(s), unspecified, with other forms of angina pectoris, Atherosclerosis of coronary artery bypass graft(s), unspecified, with unspecified angina pectoris, Atherosclerosis of autologous vein coronary artery bypass graft(s) with unstable angina pectoris, Atherosclerosis of autologous vein coronary artery bypass graft(s) with angina pectoris with documented spasm, Atherosclerosis of autologous vein coronary artery bypass graft(s) with other forms of angina pectoris, Atherosclerosis of autologous vein coronary artery bypass graft(s) with unspecified angina pectoris, Atherosclerosis of autologous artery coronary artery bypass graft(s) with unstable angina pectoris, Atherosclerosis of autologous artery coronary artery bypass graft(s) with angina pectoris with documented spasm, Atherosclerosis of autologous artery coronary artery bypass graft(s) with other forms of angina pectoris, Atherosclerosis of autologous artery coronary artery bypass graft(s) with unspecified angina pectoris, Atherosclerosis of nonautologous biological coronary artery bypass graft(s) with unstable angina pectoris, Atherosclerosis of nonautologous biological coronary artery bypass graft(s) with angina pectoris with documented spasm, Atherosclerosis of nonautologous biological coronary artery bypass graft(s) with other forms of angina pectoris, Atherosclerosis of nonautologous biological coronary artery bypass graft(s) with unspecified angina pectoris, Atherosclerosis of other coronary artery bypass graft(s) with unstable angina pectoris, Atherosclerosis of other coronary artery bypass graft(s) with angina pectoris with documented spasm, Atherosclerosis of other coronary artery bypass graft(s) with other forms of angina pectoris, Atherosclerosis of other coronary artery bypass graft(s) with unspecified angina pectoris, Atherosclerosis of coronary artery bypass graft(s) without angina pectoris , Atherosclerosis of native coronary artery of transplanted heart without angina pectoris, Atherosclerosis of bypass graft of coronary artery of transplanted heart without angina pectoris, Chronic total occlusion of coronary artery, Coronary atherosclerosis due to lipid rich plaque , Coronary atherosclerosis due to calcified coronary lesion, Other forms of chronic ischemic heart disease, Chronic ischemic heart disease, unspecified, Presence of aortocoronary bypass graft, Coronary angioplasty status |
| Hypertension | ICD9 | 401, 401.0, 401.1, 401.9, 402, 402.0, 402.00, 402.01, 402.1, 402.10, 402.11, 402.9, 402.90, 402.91, 403, 403.0, 403.00, 403.01, 403.1, 403.10, 403.11, 403.9, 403.90, 403.91, 404, 404.0, 404.00, 404.01, 404.02, 404.03, 404.1, 404.10, 404.11, 404.12, 404.13, 404.9, 404.90, 404.91, 404.92, 404.93, 405.01, 405.09, 405.11, 405.19, 405.91, 405.99, 437.2, 796.2 | Malignant essential hypertension, Benign essential hypertension, Unspecified essential hypertension, Malignant hypertensive heart disease without heart failure, Malignant hypertensive heart disease with heart failure, Benign hypertensive heart disease without heart failure, Benign hypertensive heart disease with heart failure, Unspecified hypertensive heart disease without heart failure, Unspecified hypertensive heart disease with heart failure, Hypertensive chronic kidney disease, malignant, with chronic kidney disease stage I through stage IV, or unspecified, Hypertensive chronic kidney disease, malignant, with chronic kidney disease stage V or end stage renal disease, Hypertensive chronic kidney disease, benign, with chronic kidney disease stage I through stage IV, or unspecified, Hypertensive chronic kidney disease, benign, with chronic kidney disease stage V or end stage renal disease, Hypertensive chronic kidney disease, unspecified, with chronic kidney disease stage I through stage IV, or unspecified, Hypertensive chronic kidney disease, unspecified, with chronic kidney disease stage V or end stage renal disease, Hypertensive heart and chronic kidney disease, malignant, without heart failure and with chronic kidney disease stage I through stage IV, or unspecified, Hypertensive heart and chronic kidney disease, malignant, with heart failure and with chronic kidney disease stage I through stage IV, or unspecified, Hypertensive heart and chronic kidney disease, malignant, without heart failure and with chronic kidney disease stage V or end stage renal disease, Hypertensive heart and chronic kidney disease, malignant, with heart failure and with chronic kidney disease stage V or end stage renal disease, Hypertensive heart and chronic kidney disease, benign, without heart failure and with chronic kidney disease stage I through stage IV, or unspecified, Hypertensive heart and chronic kidney disease, benign, with heart failure and with chronic kidney disease stage I through stage IV, or unspecified, Hypertensive heart and chronic kidney disease, benign, without heart failure and with chronic kidney disease stage V or end stage renal disease, Hypertensive heart and chronic kidney disease, benign, with heart failure and chronic kidney disease stage V or end stage renal disease, Hypertensive heart and chronic kidney disease, unspecified, without heart failure and with chronic kidney disease stage I through stage IV, or unspecified, Hypertensive heart and chronic kidney disease, unspecified, with heart failure and with chronic kidney disease stage I through stage IV, or unspecified, Hypertensive heart and chronic kidney disease, unspecified, without heart failure and with chronic kidney disease stage V or end stage renal disease, Hypertensive heart and chronic kidney disease, unspecified, with heart failure and chronic kidney disease stage V or end stage renal disease, Malignant renovascular hypertension, Other malignant secondary hypertension, Benign renovascular hypertension, Other benign secondary hypertension, Unspecified renovascular hypertension, Other unspecified secondary hypertension, Hypertensive encephalopathy, Elevated blood pressure reading without diagnosis of hypertension |
| Hypertension | ICD10 | I10, I11.0, I11.9, I12.0, I12.9, I13.0, I13.10, I13.11, I13.2, I15.0, I15.1, I15.2, I15.8, I15.9 | Essential (Primary) Hypertension, Hypertensive Heart Disease with Heart Failure, Hypertensive Heart Disease without Heart Failure, Hypertensive Chronic Kidney Disease with Stage 5 Chronic Kidney Disease or end stage renal disease, Hypertensive Chronic Kidney Disease with Stage 1 through Stage 4 Chronic Kidney Disease, or unspecified chronic kidney disease, Hypertensive heart and chronic kidney disease with heart failure and stage 1 through stage 4 chronic kidney disease, or unspecified chronic kidney disease , Hypertensive heart and chronic kidney disease without heart failure, with stage 1 through stage 4 chronic kidney disease, or unspecified chronic kidney disease , Hypertensive heart and chronic kidney disease without heart failure, with stage 5 chronic kidney disease, or end stage renal disease, Hypertensive heart and chronic kidney disease with heart failure and with stage 5 chronic kidney disease, or end stage renal disease, Renovascular Hypertension, Hypertension secondary to other renal disorders, Hypertension secondary to endocrine disorders, Other Secondary Hypertension, Secondary hypertension, unspecified |
| Valvular Disease | ICD9 | 35.05, 35.06, 35.1, 35.11, 35.12, 35.13, 35.14, 35.2, 35.21, 35.22, 35.23, 35.24, 35.25, 35.26, 35.27, 35.28, 35.96, 394, 394.0, 394.1, 394.2, 394.9, 396, 396.0, 396.1, 396.2, 396.3, 396.8, 396.9, V42.2, V43.3 | Endovascular replacement of aortic valve, Transapical replacement of aortic valve, Open heart valvuloplasty without replacement, unspecified valve, Open heart valvuloplasty of aortic valve without replacement, Open heart valvuloplasty of mitral valve without replacement, Open heart valvuloplasty of pulmonary valve without replacement, Open heart valvuloplasty of tricuspid valve without replacement, Open and other replacement of unspecified heart valve, Open and other replacement of aortic valve with tissue graft, Open and other replacement of aortic valve, Open and other replacement of mitral valve with tissue graft, Open and other replacement of mitral valve, Open and other replacement of pulmonary valve with tissue graft, Open and other replacement of pulmonary valve, Open and other replacement of tricuspid valve with tissue graft, Open and other replacement of tricuspid valve, Percutaneous balloon valvuloplasty, Mitral stenosis, Rheumatic Mitral Insufficiency, Mitral stenosis with insufficiency, Other unspecified mitral valve disease, Mitral valve stenosis and aortic valve stenosis, Mitral valve stenosis and aortic valve insufficiency, Mitral valve insufficiency and aortic valve stenosis, Mitral valve insufficiency and aortic valve insufficiency, Multiple involvement of mitral and aortic valves, Mitral and aortic valve diseases, unspecified, Heart valve replaced by transplant, Heart valve replaced by other means |
| Valvular Disease | ICD10 | I05.0, I05.1, I05.2, I05.8, I05.9, I06.8 , I06.9, I07.8, I07.9, I08.0, I08.1, I08.3, I08.8, I08.9, I09.1, I34.0, I34.1, I34.2, I34.8, I34.9, I35.0, I35.1, I35.2, I35.8, I35.9, I36.0, I36.1, I36.2, I36.8, I36.9, I37.0, I37.1, I37.2, I37.8, I37.9, I38 | Rheumatic Mitral Stenosis, Rheumatic Mitral Insufficiency, Rheumatic Mitral Stenosis With Insufficiency, Other Rheumatic Mitral Valve Diseases, Rheumatic mitral valve disease, unspecified, Other rheumatic aortic valve diseases, Rheumatic aortic valve disease, unspecified, Other rheumatic tricuspid valve diseases, Rheumatic tricuspid valve disease, unspecified, Rheumatic Disorders Of Both Mitral And Aortic Valves, Rheumatic Disorders Of Both Mitral And tricuspid Valves, Combined rheumatic disorders of mitral, aortic, and tricuspid valves, Other Rheumatic Multiple Valve Diseases, Rheumatic Multiple Valve Disease, Unspecified, Rheumatic diseases of endocardium, valve unspecified, Nonrheumatic mitral (valve) insufficiency, Nonrheumatic mitral (valve) prolapse, Nonrheumatic mitral (valve) stenosis, Other nonrheumatic mitral valve disorders, Nonrheumatic mitral valve disorder, unspecified, Nonrheumatic aortic (valve) stenosis, Nonrheumatic aortic (valve) insufficiency, Nonrheumatic aortic (valve) stenosis with insufficiency, Other nonrheumatic aortic valve disorders, Nonrheumatic aortic valve disorder, unspecified, Nonrheumatic tricuspid (valve) stenosis, Nonrheumatic tricuspid (valve) insufficiency, Nonrheumatic tricuspid (valve) stenosis with insufficiency, Other nonrheumatic tricuspid valve disorders, Nonrheumatic tricuspid valve disorder, unspecified, Nonrheumatic pulmonary valve stenosis, Nonrheumatic pulmonary valve insufficiency, Nonrheumatic pulmonary valve stenosis with insufficiency, Other nonrheumatic pulmonary valve disorders, Nonrheumatic pulmonary valve disorder, unspecified, Endocarditis, valve unspecified |
| Valvular Disease | CPT | 33400, 33401, 33403, 33405, 33406, 33411, 33412, 33413, 33420, 33422, 33425, 33426, 33427, 33430, 33606, 33611, 33612, 33645, 33665, 33670, 33681, 33684, 33688, 33690 | Repair Of Aortic Valve, Valvuloplasty, Open, Valvuloplasty, W/Cp Bypass, Replacement Of Aortic Valve, Replacement Of Aortic Valve, Replacement Of Aortic Valve, Replacement Of Aortic Valve, Replacement Of Aortic Valve, Revision Of Mitral Valve, Revision Of Mitral Valve, Repair Of Mitral Valve, Repair Of Mitral Valve, Repair Of Mitral Valve, Replacement Of Mitral Valve, Anastomosis/artery-aorta, Repair double ventricle , Repair double ventricle , Revision of heart veins , Repair of heart defects, Repair of heart chambers , Repair heart septum defect , Repair heart septum defect , Repair heart septum defect , Reinforce pulmonary artery |
| Stroke/TIA | ICD9 | 362.31, 362.32, 362.33, 362.34, 388.02, 430, 431, 432.9, 433.01, 433.11, 433.21, 433.31, 433.81, 433.91, 434, 434.0, 434.00, 434.01, 434.1, 434.10, 434.11, 434.9, 434.90, 434.91, 435, 435.0, 435.1, 435.2, 435.3, 435.8, 435.9, 437.1, 437.7, 437.9, 438.1, 438.10, 438.11, 438.12, 438.13, 438.14, 438.2, 438.20, 438.21, 438.22, 438.81, 438.82, 438.83, 438.89, 438.9, 997.02, V12.54 | Central retinal artery occlusion, Retinal arterial branch occlusion, Partial retinal arterial occlusion, Transient retinal arterial occlusion, Transient ischemic deafness, Subarachnoid hemorrhage, Intracerebral hemorrhage, Unspecified intracranial hemorrhage, Occlusion and stenosis of basilar artery with cerebral infarction, Occlusion and stenosis of carotid artery with cerebral infarction, Occlusion and stenosis of vertebral artery with cerebral infarction, Occlusion and stenosis of multiple and bilateral precerebral arteries with cerebral infarction, Occlusion and stenosis of other specified precerebral artery with cerebral infarction, Occlusion and stenosis of unspecified precerebral artery with cerebral infarction, Cerebral thrombosis without mention of cerebral infarction, Cerebral thrombosis with cerebral infarction, Cerebral embolism without mention of cerebral infarction, Cerebral embolism with cerebral infarction, Cerebral artery occlusion, unspecified without mention of cerebral infarction, Cerebral artery occlusion, unspecified with cerebral infarction, Basilar artery syndrome, Vertebral artery syndrome , Subclavian steal syndrome, Vertebrobasilar artery syndrome, Other specified transient cerebral ischemias, Unspecified transient cerebral ischemia, Other generalized ischemic cerebrovascular disease, Transient global amnesia, Unspecified cerebrovascular disease, Late effects of cerebrovascular disease, speech and language deficit, unspecified, Late effects of cerebrovascular disease, aphasia, Late effects of cerebrovascular disease, dysphasia, Late effects of cerebrovascular disease, dysarthria, Late effects of cerebrovascular disease, fluency disorder, Late effects of cerebrovascular disease, hemiplegia affecting unspecified side, Late effects of cerebrovascular disease, hemiplegia affecting dominant side, Late effects of cerebrovascular disease, hemiplegia affecting nondominant side, Other late effects of cerebrovascular disease, apraxia, Other late effects of cerebrovascular disease, dysphagia, Other late effects of cerebrovascular disease, facial weakness, Other late effects of cerebrovascular disease, Unspecified late effects of cerebrovascular disease, Iatrogenic cerebrovascular infarction or hemorrhage, Personal history of transient ischemic attack (TIA), and cerebral infarction without residual deficits |
| Stroke/TIA | IDC10 | G45.0, G45.1, G45.2, G45.3, G45.4, G45.8, G46.3, G46.4, H34.00, H34.01, H34.02, H34.03, H34.10, H34.11, H34.12, H34.13, H34.211, H34.212, H34.213, H34.219, H34.231, H34.232, H34.233, H34.239, H93.099, I60.9, I61.9, I62.9, I63.00, I63.011, I63.012, I63.019, I63.111, I63.112, I63.119, I63.12, I63.131, I63.132, I63.139, I63.19, I63.20, I63.211, I63.212, I63.219, I63.22, I63.231, I63.232, I63.239, I63.29, I63.30, I63.311, I63.312, I63.319, I63.321, I63.322, I63.329, I63.331, I63.332, I63.339, I63.341, I63.342, I63.349, I63.40, I63.411, I63.412, I63.419, I63.421, I63.422, I63.429, I63.431, I63.432, I63.439, I63.49, I63.50, I63.511, I63.512, I63.519, I63.521, I63.522, I63.529, I63.531, I63.532, I63.539, I63.541, I63.542, I63.549, I63.59, I63.6, I63.8, I63.9, I66.01, I66.02, I66.03, I66.09, I66.11, I66.12, I66.13, I66.19, I66.21, I66.22, I66.23, I66.29, I66.3, I66.8, I66.9, I67.81, I67.82, I67.841, I67.848, I67.89, I67.9, I69.80, I69.81, I69.820, I69.821, I69.822, I69.823, I69.828, I69.831, I69.832, I69.833, I69.834, I69.839, I69.841, I69.842, I69.843, I69.844, I69.849, I69.851, I69.852, I69.853, I69.854, I69.859, I69.861, I69.862, I69.863, I69.864, I69.865, I69.869, I69.890, I69.891, I69.892, I69.893, I69.898, I69.90, I69.91, I69.920, I69.921, I69.922, I69.923, I69.928, I69.931, I69.932, I69.933, I69.934, I69.939, I69.941, I69.942, I69.943, I69.944, I69.949, I69.951, I69.952, I69.953, I69.954, I69.959, I69.961, I69.962, I69.963, I69.964, I69.965, I69.969, I69.990, I69.991, I69.992, I69.993, I69.998, I97.810, I97.811, I97.820, I97.821,  Z86.73 | Vertebro-Basilar Artery Syndrome, Carotid Artery Syndrome, Multiple and bilateral precerebral artery syndromes, Amaurosis fugax, Transient Global Amnesia, Other transient cerebral ischemic attacks and related syndromes, Brain stem stroke syndrome, Cerebellar stroke syndrome, Transient Retinal Artery Occlusion, Unspecified Eye, Transient retinal artery occlusion, right eye, Transient retinal artery occlusion, left eye, Transient retinal artery occlusion, bilateral, Central retinal artery occlusion, unspecified eye, Central retinal artery occlusion, right eye, Central retinal artery occlusion, left eye, Central retinal artery occlusion, bilateral, Partial retinal artery occlusion, right eye, Partial retinal artery occlusion, left eye, Partial retinal artery occlusion, bilateral, Partial Retinal Artery Occlusion, Unspecified Eye, Retinal artery branch occlusion, right eye, Retinal artery branch occlusion, left eye, Retinal artery branch occlusion, bilateral, Retinal Artery Branch Occlusion, Unspecified Eye, Unspecified Degenerative and Vascular Disorders of Unspecified Ear, Nontraumatic Subarachnoid Hemorrhage, Unspecified, Nontraumatic intracerebral Hemorrhage, unspecified, Nontraumatic Intracranial Hemorrhage, Unspecified, Cerebral infarction due to thrombosis of unspecified precerebral artery, Cerebral infarction due to thrombosis of right vertebral artery, Cerebral infarction due to thrombosis of left vertebral artery, Cerebral infarction due to thrombosis of unspecified vertebral artery, Cerebral infarction due to embolism of right vertebral artery, Cerebral infarction due to embolism of left vertebral artery, Cerebral infarction due to embolism of unspecified vertebral artery , Cerebral infarction due to embolism of basilar artery, Cerebral infarction due to embolism of right carotid artery, Cerebral infarction due to embolism of left carotid artery, Cerebral infarction due to embolism of unspecified carotid artery, Cerebral infarction due to embolism of other precerebral artery, Cerebral infarction due to unspecified occlusion or stenosis of unspecified precerebral arteries , Cerebral infarction due to unspecified occlusion or stenosis of right vertebral arteries, Cerebral infarction due to unspecified occlusion or stenosis of left vertebral arteries, Cerebral infarction due to unspecified occlusion or stenosis of unspecified vertebral arteries, Cerebral Infraction Due to Unspecified Occlusion or Stenosis of Basilar Arteries, Cerebral infarction due to unspecified occlusion or stenosis of right carotid arteries, Cerebral infarction due to unspecified occlusion or stenosis of left carotid arteries, Cerebral infarction due to unspecified occlusion or stenosis of unspecified carotid arteries, Cerebral infarction due to unspecified occlusion or stenosis of other precerebral arteries, Cerebral infarction due to thrombosis of unspecified cerebral artery , Cerebral infarction due to thrombosis of right middle cerebral artery, Cerebral infarction due to thrombosis of left middle cerebral artery, Cerebral infarction due to thrombosis of unspecified middle cerebral artery, Cerebral infarction due to thrombosis of right anterior cerebral artery, Cerebral infarction due to thrombosis of left anterior cerebral artery, Cerebral infarction due to thrombosis of unspecified anterior cerebral artery, Cerebral infarction due to thrombosis of right posterior cerebral artery, Cerebral infarction due to thrombosis of left posterior cerebral artery, Cerebral infarction due to thrombosis of unspecified posterior cerebral artery, Cerebral infarction due to thrombosis of right cerebellar artery, Cerebral infarction due to thrombosis of left cerebellar artery, Cerebral infarction due to thrombosis of unspecified cerebellar artery, Cerebral infarction due to embolism of unspecified cerebral artery , Cerebral infarction due to embolism of right middle cerebral artery, Cerebral infarction due to embolism of left middle cerebral artery, Cerebral infarction due to embolism of unspecified middle cerebral artery, Cerebral infarction due to embolism of right anterior cerebral artery, Cerebral infarction due to embolism of left anterior cerebral artery, Cerebral infarction due to embolism of unspecified anterior cerebral artery, Cerebral infarction due to embolism of right posterior cerebral artery, Cerebral infarction due to embolism of left posterior cerebral artery, Cerebral infarction due to embolism of unspecified posterior cerebral artery , Cerebral infarction due to embolism of other cerebral artery, Cerebral infarction due to unspecified occlusion or stenosis of unspecified cerebral artery , Cerebral infarction due to unspecified occlusion or stenosis of right middle cerebral artery, Cerebral infarction due to unspecified occlusion or stenosis of left middle cerebral artery, Cerebral infarction due to unspecified occlusion or stenosis of unspecified middle cerebral artery, Cerebral infarction due to unspecified occlusion or stenosis of right anterior cerebral artery, Cerebral infarction due to unspecified occlusion or stenosis of left anterior cerebral artery, Cerebral infarction due to unspecified occlusion or stenosis of unspecified anterior cerebral artery, Cerebral infarction due to unspecified occlusion or stenosis of right posterior cerebral artery, Cerebral infarction due to unspecified occlusion or stenosis of left posterior cerebral artery, Cerebral infarction due to unspecified occlusion or stenosis of unspecified posterior cerebral artery, Cerebral infarction due to unspecified occlusion or stenosis of right cerebellar artery, Cerebral infarction due to unspecified occlusion or stenosis of left cerebellar artery, Cerebral infarction due to unspecified occlusion or stenosis of unspecified cerebellar artery, Cerebral infarction due to unspecified occlusion or stenosis of other cerebral artery , Cerebral infarction due to cerebral venous thrombosis, nonpyogenic, Other cerebral infarction, Cerebral infarction, unspecified, Occlusion and stenosis of right middle cerebral artery, Occlusion and stenosis of left middle cerebral artery, Occlusion and stenosis of bilateral middle cerebral arteries, Occlusion and stenosis of unspecified middle cerebral artery, Occlusion and stenosis of right anterior cerebral artery, Occlusion and stenosis of left anterior cerebral artery, Occlusion and stenosis of bilateral anterior cerebral arteries, Occlusion and stenosis of unspecified anterior cerebral artery, Occlusion and stenosis of right posterior cerebral artery, Occlusion and stenosis of left posterior cerebral artery, Occlusion and stenosis of bilateral posterior cerebral arteries, Occlusion and stenosis of unspecified posterior cerebral artery , Occlusion and stenosis of cerebellar arteries, Occlusion and stenosis of other cerebral arteries, Occlusion and stenosis of unspecified cerebral artery, Acute cerebrovascular insufficiency, Cerebral ischemia, Reversible cerebrovascular vasoconstriction syndrome, Other cerebrovascular vasospasm and vasoconstriction , Other cerebrovascular disease, Cerebrovascular Disease, unspecified, Unspecified sequelae of other cerebrovascular disease, Cognitive deficits following other cerebrovascular disease, Aphasia following other cerebrovascular disease, Dysphasia following other cerebrovascular disease, Dysarthria following other cerebrovascular disease, Fluency disorder following other cerebrovascular disease, Other speech and language deficits following other cerebrovascular disease, Monoplegia of upper limb following other cerebrovascular disease affecting right dominant side, Monoplegia of upper limb following other cerebrovascular disease affecting left dominant side, Monoplegia of upper limb following other cerebrovascular disease affecting right non-dominant side, Monoplegia of upper limb following other cerebrovascular disease affecting left non-dominant side, Monoplegia of upper limb following other cerebrovascular disease affecting unspecified side, Monoplegia of lower limb following other cerebrovascular disease affecting right dominant side, Monoplegia of lower limb following other cerebrovascular disease affecting left dominant side, Monoplegia of lower limb following other cerebrovascular disease affecting right non-dominant side, Monoplegia of lower limb following other cerebrovascular disease affecting left non-dominant side, Monoplegia of lower limb following other cerebrovascular disease affecting unspecified side, Hemiplegia and hemiparesis following other cerebrovascular disease affecting right dominant side, Hemiplegia and hemiparesis following other cerebrovascular disease affecting left dominant side, Hemiplegia and hemiparesis following other cerebrovascular disease affecting right non-dominant side, Hemiplegia and hemiparesis following other cerebrovascular disease affecting left non-dominant side, Hemiplegia and hemiparesis following other cerebrovascular disease affecting unspecified side, Other paralytic syndrome following other cerebrovascular disease affecting right dominant side, Other paralytic syndrome following other cerebrovascular disease affecting left dominant side, Other paralytic syndrome following other cerebrovascular disease affecting right non-dominant side, Other paralytic syndrome following other cerebrovascular disease affecting left non-dominant side, Other paralytic syndrome following other cerebrovascular disease, bilateral, Other paralytic syndrome following other cerebrovascular disease affecting unspecified side, Apraxia following other cerebrovascular disease, Dysphagia following other cerebrovascular disease, Facial weakness following other cerebrovascular disease, Ataxia following other cerebrovascular disease, Other sequelae of other cerebrovascular disease , Unspecified Sequelae of unspecified cerebrovascular disease, Cognitive deficits following unspecified cerebrovascular disease, Aphasia following unspecified cerebrovascular disease, Dysphasia following unspecified cerebrovascular disease , Dysarthria following unspecified Cerebrovascular disease, Fluency disorder following unspecified cerebrovascular disease , Other speech and language deficits following unspecified cerebrovascular disease, Monoplegia of upper limb following unspecified cerebrovascular disease affecting right dominant side, Monoplegia of upper limb following unspecified cerebrovascular disease affecting left dominant side, Monoplegia of upper limb following unspecified cerebrovascular disease affecting right non-dominant side, Monoplegia of upper limb following unspecified cerebrovascular disease affecting left non-dominant side, Monoplegia of upper limb following unspecified cerebrovascular disease affecting unspecified side, Monoplegia of lower limb following unspecified cerebrovascular disease affecting right dominant side, Monoplegia of lower limb following unspecified cerebrovascular disease affecting left dominant side, Monoplegia of lower limb following unspecified cerebrovascular disease affecting right non-dominant side, Monoplegia of lower limb following unspecified cerebrovascular disease affecting left non-dominant side, Monoplegia of lower limb following unspecified cerebrovascular disease affecting unspecified side, Hemiplegia and hemiparesis following unspecified cerebrovascular disease affecting right dominant side , Hemiplegia and hemiparesis following unspecified cerebrovascular disease affecting left dominant side , Hemiplegia and hemiparesis following unspecified cerebrovascular disease affecting right non-dominant side, Hemiplegia and hemiparesis following unspecified cerebrovascular disease affecting left non-dominant side , Hemiplegia and hemiparesis following unspecified cerebrovascular disease affecting unspecified side , Other paralytic syndrome following unspecified cerebrovascular disease affecting right dominant side, Other paralytic syndrome following unspecified cerebrovascular disease affecting left dominant side, Other paralytic syndrome following unspecified cerebrovascular disease affecting right non-dominant side, Other paralytic syndrome following unspecified cerebrovascular disease affecting left non-dominant side, Other paralytic syndrome following unspecified cerebrovascular disease, bilateral, Other paralytic syndrome following unspecified cerebrovascular disease affecting unspecified side, Apraxia following unspecified cerebrovascular disease, Dysphagia following unspecified cerebrovascular disease , Facial weakness following unspecified cerebrovascular disease , Ataxia following unspecified cerebrovascular disease, Other sequelae following unspecified cerebrovascular disease , Intraoperative Cerebrovascular Infarction During cardiac surgery, Intraoperative cerebrovascular infarction during other surgery , Postprocedural cerebrovascular infarction during cardiac surgery, Postprocedural cerebrovascular infarction during other surgery, Personal history of transient ischemic attack (TIA), and cerebral infarction without residual deficits |
| Myocardial Infarction | ICD9 | 410, 410.0, 410.00, 410.01, 410.02, 410.1, 410.10, 410.11, 410.12, 410.2, 410.20, 410.21, 410.22, 410.3, 410.30, 410.31, 410.32, 410.4, 410.40, 410.41, 410.42, 410.5, 410.50, 410.51, 410.52, 410.6, 410.60, 410.61, 410.62, 410.7, 410.70, 410.71, 410.72, 410.8, 410.80, 410.81, 410.82, 410.9, 410.90, 410.91, 410.92, 412, 429.79 | Acute myocardial infarction of anterolateral wall, episode of care unspecified, Acute myocardial infarction of anterolateral wall, initial episode of care, Acute myocardial infarction of anterolateral wall, subsequent episode of care , Acute myocardial infarction of other anterior wall, episode of care unspecified, Acute myocardial infarction of other anterior wall, initial episode of care, Acute myocardial infarction of other anterior wall, subsequent episode of care, Acute myocardial infarction of inferolateral wall, episode of care unspecified, Acute myocardial infarction of inferolateral wall, initial episode of care, Acute myocardial infarction of inferolateral wall, subsequent episode of care, Acute myocardial infarction of inferoposterior wall, episode of care unspecified, Acute myocardial infarction of inferoposterior wall, initial episode of care, Acute myocardial infarction of inferoposterior wall, subsequent episode of care, Acute myocardial infarction of other inferior wall, episode of care unspecified, Acute myocardial infarction of other inferior wall, initial episode of care, Acute myocardial infarction of other inferior wall, subsequent episode of care, Acute myocardial infarction of other lateral wall, episode of care unspecified, Acute myocardial infarction of other lateral wall, initial episode of care, Acute myocardial infarction of other lateral wall, subsequent episode of care, True posterior wall infarction, episode of care unspecified, True posterior wall infarction, initial episode of care, True posterior wall infarction, subsequent episode of care, Subendocardial infarction, episode of care unspecified, Subendocardial infarction, initial episode of care, Subendocardial infarction, subsequent episode of care, Acute myocardial infarction of other specified sites, episode of care unspecified, Acute myocardial infarction of other specified sites, initial episode of care, Acute myocardial infarction of other specified sites, subsequent episode of care, Acute myocardial infarction of unspecified site, episode of care unspecified, Acute myocardial infarction of unspecified site, initial episode of care, Acute myocardial infarction of unspecified site, subsequent episode of care, Old myocardial infarction , Certain sequelae of myocardial infarction, not elsewhere classified, other |
| Myocardial Infarction | ICD10 | I21.01, I21.02, I21.09, I21.11, I21.19, I21.21, I21.29, I21.3, I21.4, I22.0, I22.1, I22.2, I22.8, I22.9, I23.0, I23.1, I23.2, I23.3, I23.4, I23.5, I23.6, I23.7, I23.8, I24.1, I25.2 | ST elevation (STEMI) myocardial infarction involving left main coronary artery, ST elevation (STEMI) myocardial infarction involving left anterior descending coronary artery, ST elevation (STEMI) myocardial infarction involving other coronary artery of anterior wall, ST elevation (STEMI) myocardial infarction involving right coronary artery, ST elevation (STEMI) myocardial infarction involving other coronary artery of inferior wall, ST elevation (STEMI) myocardial infarction involving left circumflex coronary artery, ST elevation (STEMI) myocardial infarction involving other sites, ST elevation (STEMI) myocardial infarction of unspecified site , Non-ST elevation (NSTEMI) myocardial infarction , Subsequent ST elevation (STEMI) myocardial infarction of anterior wall, Subsequent ST elevation (STEMI) myocardial infarction of inferior wall, Subsequent non-ST elevation (NSTEMI) myocardial infarction , Subsequent ST elevation (STEMI) myocardial infarction of other sites, Subsequent ST elevation (STEMI) myocardial infarction of unspecified site, Hemopericardium as current complication following acute myocardial infarction, Atrial septal defect as current complication following acute myocardial infarction, Ventricular septal defect as current complication following acute myocardial infarction, Rupture of cardiac wall without hemopericardium as current complication following acute myocardial infarction, Rupture of chordae tendineae as current complication following acute myocardial infarction, Rupture of papillary muscle as current complication following acute myocardial infarction, Thrombosis of atrium, auricular appendage, and ventricle as current complications following acute myocardial infarction, Postinfarction angina, Other current complications following acute myocardial infarction, Dressler's syndrome, Old myocardial infarction |
| Peripheral Artery Disease | ICD9 | 440, 440.0, 440.1, 440.10, 440.2, 440.20, 440.21, 440.22, 440.23, 440.24, 440.29, 440.3, 440.30, 440.31, 440.32, 440.4, 440.40, 440.8, 440.80, 440.9, 440.90, 442, 442.1, 442.2, 442.3, 442.81, 442.82, 442.83, 442.84, 442.89, 442.9, 443.21, 443.22, 443.23, 443.24, 443.29, 443.81, 443.89, 443.89, 443.9, 443.9, V43.4 | Aortic atherosclerosis, Atherosclerosis of renal artery, Atherosclerosis of native arteries of the extremities, unspecified, Atherosclerosis of native arteries of the extremities with intermittent claudication, Atherosclerosis of native arteries of the extremities with rest pain, Atherosclerosis of native arteries with ulceration, Atherosclerosis of native arteries of the extremities with gangrene, Other atherosclerosis of native arteries of the extremities, Atherosclerosis of unspecified bypass graft of the extremities, Atherosclerosis of autologous vein bypass graft of the extremities, Atherosclerosis of nonautologous biological bypass graft of the extremities, Chronic total occlusion of artery of the extremities, Atherosclerosis of other specified arteries, Generalized and unspecified atherosclerosis, Upper extremity aneurysm, Aneurysm of renal artery,  Aneurysm of renal artery,  Aneurysm of artery of lower extremity, Aneurysm of artery of neck, Aneurysm of subclavian artery, Aneurysm of splenic artery, Aneurysm of other visceral artery, Aneurysm of other specified artery, Aneurysm of unspecified site, Dissection of carotid artery, Dissection of iliac artery, Dissection of renal artery, Dissection of vertebral artery, Dissection of other artery, Peripheral angiopathy in diseases classified elsewhere, Other specified peripheral vascular diseases, Other specified peripheral vascular diseases, Peripheral vascular disease, unspecified, Peripheral vascular disease, unspecified, Blood vessel replaced by other means |
| Peripheral Artery Disease | ICD10 | I170.203, I70.1, I70.201, I70.202, I70.208, I70.209, I70.211, I70.212, I70.213, I70.218, I70.219, I70.221, I70.222, I70.223, I70.228, I70.229, I70.231, I70.232, I70.233, I70.234, I70.235, I70.238, I70.239, I70.241, I70.242, I70.243, I70.244, I70.245, I70.248, I70.249, I70.25, I70.261, I70.262, I70.263, I70.268, I70.269, I70.291, I70.292, I70.293, I70.298, I70.299, I70.301, I70.302, I70.303, I70.308, I70.309, I70.311, I70.312, I70.313, I70.318, I70.319, I70.321, I70.322, I70.323, I70.328, I70.329, I70.331, I70.332, I70.333, I70.334, I70.335, I70.338, I70.339, I70.341, I70.342, I70.343, I70.344, I70.345, I70.348, I70.349, I70.35, I70.361, I70.362, I70.363, I70.368, I70.369, I70.391, I70.392, I70.393, I70.398, I70.399, I70.501, I70.502, I70.503, I70.508, I70.509, I70.511, I70.512, I70.513, I70.518, I70.519, I70.521, I70.522, I70.523, I70.528, I70.529, I70.531, I70.532, I70.533, I70.534, I70.535, I70.538, I70.539, I70.541, I70.542, I70.543, I70.544, I70.545, I70.548, I70.549, I70.55, I70.561, I70.562, I70.563, I70.568, I70.569, I70.591, I70.592, I70.593, I70.598, I70.599, I70.601, I70.602, I70.603, I70.608, I70.609, I70.611, I70.612, I70.613, I70.618, I70.619, I70.621, I70.622, I70.623, I70.628, I70.629, I70.631, I70.632, I70.633, I70.634, I70.635, I70.638, I70.639, I70.641, I70.642, I70.643, I70.644, I70.645, I70.648, I70.649, I70.65, I70.661, I70.662, I70.663, I70.668, I70.669, I70.691, I70.692, I70.693, I70.698, I70.699, I70.701, I70.702, I70.703, I70.708, I70.709, I70.711, I70.712, I70.713, I70.718, I70.719, I70.721, I70.722, I70.723, I70.728, I70.729, I70.731, I70.732, I70.733, I70.734, I70.735, I70.738, I70.739, I70.741, I70.742, I70.743, I70.744, I70.745, I70.748, I70.749, I70.75, I70.761, I70.762, I70.763, I70.768, I70.769, I70.791, I70.792, I70.793, I70.798, I70.799, I70.8, I70.90, I70.91, I70.92, I72.0, I72.1, I72.2, I72.3, I72.4, I72.8, I72.9, I73.89, I73.9, I77.71, I77.72, I77.73, I77.74, I77.79, I79.8, Z95.820, Z95.828 | Unspecified atherosclerosis of native arteries of extremities, bilateral legs, Atherosclerosis of renal artery, Unspecified atherosclerosis of native arteries of extremities, right leg, Unspecified atherosclerosis of native arteries of extremities, left leg, Unspecified atherosclerosis of native arteries of extremities, other extremity, Unspecified atherosclerosis of native arteries of extremities, unspecified extremity , Atherosclerosis of native arteries of extremities with intermittent claudication, right leg, Atherosclerosis of native arteries of extremities with intermittent claudication, left leg, Atherosclerosis of native arteries of extremities with intermittent claudication, bilateral legs, Atherosclerosis of native arteries of extremities with intermittent claudication, other extremity , Atherosclerosis of native arteries of extremities with intermittent claudication, unspecified extremity , Atherosclerosis of native extremities with rest pain, right leg, Atherosclerosis of native extremities with rest pain, left leg, Atherosclerosis of native extremities with rest pain, bilateral legs, Atherosclerosis of native arteries of extremities with rest pain, other extremity, Atherosclerosis of native arteries of extremities with rest pain, unspecified extremity, Atherosclerosis of native arteries of right leg with ulceration of thigh, Atherosclerosis of native arteries of right leg with ulceration of calf, Atherosclerosis of native arteries of right leg with ulceration of ankle, Atherosclerosis of native arteries of right leg with ulceration of heel and midfoot, Atherosclerosis of native arteries of right leg with ulceration of other part of foot, Atherosclerosis of native arteries of right leg with ulceration of other part of lower right leg, Atherosclerosis of native arteries of right leg with ulceration of unspecified site, Atherosclerosis of native arteries of left leg with ulceration of thigh, Atherosclerosis of native arteries of left leg with ulceration of calf, Atherosclerosis of native arteries of left leg with ulceration of ankle, Atherosclerosis of native arteries of left leg with ulceration of heel and midfoot, Atherosclerosis of native arteries of left leg with ulceration of other part of foot, Atherosclerosis of native arteries of left leg with ulceration of other part of lower right leg, Atherosclerosis of native arteries of left leg with ulceration of unspecified site, Atherosclerosis of native arteries of other extremities with ulceration, Atherosclerosis of native arteries of extremities with gangrene, right leg, Atherosclerosis of native arteries of extremities with gangrene, left leg, Atherosclerosis of native arteries of extremities with gangrene, bilateral legs, Atherosclerosis of native arteries of extremities with gangrene, other extremity, Atherosclerosis of native arteries of extremities with gangrene, unspecified extremity, Other atherosclerosis of native arteries of extremities, right leg, Other atherosclerosis of native arteries of extremities, left leg, Other atherosclerosis of native arteries of extremities, bilateral legs, Other atherosclerosis of native arteries of extremities, other extremity, Other atherosclerosis of native arteries of extremities, unspecified extremity, Unspecified atherosclerosis of unspecified type of bypass graft(s) of the extremities, right leg, Unspecified atherosclerosis of unspecified type of bypass graft(s) of the extremities, left leg, Unspecified atherosclerosis of unspecified type of bypass graft(s) of the extremities, bilateral legs, Unspecified atherosclerosis of unspecified type of bypass graft(s) of the extremities, other extremity, Unspecified atherosclerosis of unspecified type of bypass graft(s) of the extremities, unspecified extremity, Atherosclerosis of unspecified type of bypass graft(s) of the extremities with intermittent claudication, right leg, Atherosclerosis of unspecified type of bypass graft(s) of the extremities with intermittent claudication, left leg, Atherosclerosis of unspecified type of bypass graft(s) of the extremities with intermittent claudication, bilateral legs, Atherosclerosis of unspecified type of bypass graft(s) of the extremities with intermittent claudication, other extremity, Atherosclerosis of unspecified type of bypass graft(s) of the extremities with intermittent claudication, unspecified extremity, Atherosclerosis of unspecified type of bypass graft(s) of the extremities with rest pain, right leg, Atherosclerosis of unspecified type of bypass graft(s) of the extremities with rest pain, left leg, Atherosclerosis of unspecified type of bypass graft(s) of the extremities with rest pain, bilateral legs, Atherosclerosis of unspecified type of bypass graft(s) of the extremities with rest pain, other extremity, Atherosclerosis of unspecified type of bypass graft(s) of the extremities with rest pain, unspecified extremity, Atherosclerosis of unspecified type of bypass graft(s) of the right leg with ulceration of the thigh, Atherosclerosis of unspecified type of bypass graft(s) of the right leg with ulceration of the calf, Atherosclerosis of unspecified type of bypass graft(s) of the right leg with ulceration of the ankle, Atherosclerosis of unspecified type of bypass graft(s) of the right leg with ulceration of the heel and midfoot, Atherosclerosis of unspecified type of bypass graft(s) of the right leg with ulceration of other part of foot, Atherosclerosis of unspecified type of bypass graft(s) of the right leg with ulceration of other part of lower leg, Atherosclerosis of unspecified type of bypass graft(s) of the right leg with ulceration of unspecified site, Atherosclerosis of unspecified type of bypass graft(s) of the left leg with ulceration of the thigh, Atherosclerosis of unspecified type of bypass graft(s) of the left leg with ulceration of the calf, Atherosclerosis of unspecified type of bypass graft(s) of the left leg with ulceration of the ankle, Atherosclerosis of unspecified type of bypass graft(s) of the left leg with ulceration of the heel and midfoot, Atherosclerosis of unspecified type of bypass graft(s) of the left leg with ulceration of other part of foot, Atherosclerosis of unspecified type of bypass graft(s) of the left leg with ulceration of other part of lower leg, Atherosclerosis of unspecified type of bypass graft(s) of the left leg with ulceration of unspecified site, Atherosclerosis of unspecified type of bypass graft(s) of other extremity with ulceration, Atherosclerosis of unspecified type of bypass graft(s) of the extremities with gangrene, right leg, Atherosclerosis of unspecified type of bypass graft(s) of the extremities with gangrene, left leg, Atherosclerosis of unspecified type of bypass graft(s) of the extremities with gangrene, bilateral legs, Atherosclerosis of unspecified type of bypass graft(s) of the extremities with gangrene, other extremity, Atherosclerosis of unspecified type of bypass graft(s) of the extremities with gangrene, unspecified extremity, Other atherosclerosis of unspecified type of bypass graft(s) of the extremities, right leg, Other atherosclerosis of unspecified type of bypass graft(s) of the extremities, left leg, Other atherosclerosis of unspecified type of bypass graft(s) of the extremities, bilateral legs, Other atherosclerosis of unspecified type of bypass graft(s) of the extremities, other extremity, Other atherosclerosis of unspecified type of bypass graft(s) of the extremities, unspecified extremity, Unspecified atherosclerosis of nonautologous biological bypass graft(s) of the extremities, right leg, Unspecified atherosclerosis of nonautologous biological bypass graft(s) of the extremities, left leg, Unspecified atherosclerosis of nonautologous biological bypass graft(s) of the extremities, bilateral legs, Unspecified atherosclerosis of nonautologous biological bypass graft(s) of the extremities, other extremity, Unspecified atherosclerosis of nonautologous biological bypass graft(s) of the extremities, unspecified extremity, Atherosclerosis of nonautologous biological bypass graft(s) of the extremities with intermittent claudication, right leg, Atherosclerosis of nonautologous biological bypass graft(s) of the extremities with intermittent claudication, left leg, Atherosclerosis of nonautologous biological bypass graft(s) of the extremities with intermittent claudication, bilateral legs, Atherosclerosis of nonautologous biological bypass graft(s) of the extremities with intermittent claudication, other extremity, Atherosclerosis of nonautologous biological bypass graft(s) of the extremities with intermittent claudication, unspecified extremity, Atherosclerosis of nonautologous biological bypass graft(s) of the extremities with rest pain, right leg, Atherosclerosis of nonautologous biological bypass graft(s) of the extremities with rest pain, left leg, Atherosclerosis of nonautologous biological bypass graft(s) of the extremities with rest pain, bilateral legs, Atherosclerosis of nonautologous biological bypass graft(s) of the extremities with rest pain, other extremity, Atherosclerosis of nonautologous biological bypass graft(s) of the extremities with rest pain, unspecified extremity, Atherosclerosis of nonautologous biological bypass graft(s) of the right leg with ulceration of thigh, Atherosclerosis of nonautologous biological bypass graft(s) of the right leg with ulceration of calf, Atherosclerosis of nonautologous biological bypass graft(s) of the right leg with ulceration of ankle, Atherosclerosis of nonautologous biological bypass graft(s) of the right leg with ulceration of heel and midfoot, Atherosclerosis of nonautologous biological bypass graft(s) of the right leg with ulceration of other part of foot, Atherosclerosis of nonautologous biological bypass graft(s) of the right leg with ulceration of other part of lower leg, Atherosclerosis of nonautologous biological bypass graft(s) of the right leg with ulceration of unspecified site, Atherosclerosis of nonautologous biological bypass graft(s) of the left leg with ulceration of thigh , Atherosclerosis of nonautologous biological bypass graft(s) of the left leg with ulceration of calf, Atherosclerosis of nonautologous biological bypass graft(s) of the left leg with ulceration of ankle, Atherosclerosis of nonautologous biological bypass graft(s) of the left leg with ulceration of heel and midfoot, Atherosclerosis of nonautologous biological bypass graft(s) of the left leg with ulceration of other part of foot, Atherosclerosis of nonautologous biological bypass graft(s) of the left leg with ulceration of other part of lower leg, Atherosclerosis of nonautologous biological bypass graft(s) of the left leg with ulceration of unspecified site, Atherosclerosis of nonautologous biological bypass graft(s) of other extremity with ulceration, Atherosclerosis of nonautologous biological bypass graft(s) of the extremities with gangrene, right leg, Atherosclerosis of nonautologous biological bypass graft(s) of the extremities with gangrene, left leg, Atherosclerosis of nonautologous biological bypass graft(s) of the extremities with gangrene, bilateral legs, Atherosclerosis of nonautologous biological bypass graft(s) of the extremities with gangrene, other extremity, Atherosclerosis of nonautologous biological bypass graft(s) of the extremities with gangrene, unspecified extremity, Other atherosclerosis of nonautologous biological bypass graft(s) of the extremities, right leg, Other atherosclerosis of nonautologous biological bypass graft(s) of the extremities, left leg, Other atherosclerosis of nonautologous biological bypass graft(s) of the extremities, bilateral legs, Other atherosclerosis of nonautologous biological bypass graft(s) of the extremities, other extremity, Other atherosclerosis of nonautologous biological bypass graft(s) of the extremities, unspecified extremity, Unspecified atherosclerosis of nonbiological bypass graft(s) of the extremities, right leg, Unspecified atherosclerosis of nonbiological bypass graft(s) of the extremities, left leg, Unspecified atherosclerosis of nonbiological bypass graft(s) of the extremities, bilateral legs, Unspecified atherosclerosis of nonbiological bypass graft(s) of the extremities, other extremity, Unspecified atherosclerosis of nonbiological bypass graft(s) of the extremities, unspecified extremity, Atherosclerosis of nonbiological bypass graft(s) of the extremities with intermittent claudication, right leg, Atherosclerosis of nonbiological bypass graft(s) of the extremities with intermittent claudication, left leg, Atherosclerosis of nonbiological bypass graft(s) of the extremities with intermittent claudication, bilateral legs, Atherosclerosis of nonbiological bypass graft(s) of the extremities with intermittent claudication, other extremity, Atherosclerosis of nonbiological bypass graft(s) of the extremities with intermittent claudication, unspecified extremity, Atherosclerosis of nonbiological bypass graft(s) of the extremities with rest pain, right leg, Atherosclerosis of nonbiological bypass graft(s) of the extremities with rest pain, left leg, Atherosclerosis of nonbiological bypass graft(s) of the extremities with rest pain, bilateral legs, Atherosclerosis of nonbiological bypass graft(s) of the extremities with rest pain, other extremity, Atherosclerosis of nonbiological bypass graft(s) of the extremities with rest pain, unspecified extremity, Atherosclerosis of nonbiological bypass graft(s) of the right leg with ulceration of thigh, Atherosclerosis of nonbiological bypass graft(s) of the right leg with ulceration of calf, Atherosclerosis of nonbiological bypass graft(s) of the right leg with ulceration of ankle, Atherosclerosis of nonbiological bypass graft(s) of the right leg with ulceration of heel and midfoot, Atherosclerosis of nonbiological bypass graft(s) of the right leg with ulceration of other part of foot, Atherosclerosis of nonbiological bypass graft(s) of the right leg with ulceration of other part of lower leg, Atherosclerosis of nonbiological bypass graft(s) of the right leg with ulceration of unspecified site, Atherosclerosis of nonbiological bypass graft(s) of the left leg with ulceration of thigh, Atherosclerosis of nonbiological bypass graft(s) of the left leg with ulceration of calf, Atherosclerosis of nonbiological bypass graft(s) of the left leg with ulceration of ankle, Atherosclerosis of nonbiological bypass graft(s) of the left leg with ulceration of heel and midfoot, Atherosclerosis of nonbiological bypass graft(s) of the left leg with ulceration of other part of foot, Atherosclerosis of nonbiological bypass graft(s) of the left leg with ulceration of other part of lower leg, Atherosclerosis of nonbiological bypass graft(s) of the left leg with ulceration of unspecified site, Atherosclerosis of nonbiological bypass graft(s) of other extremity with ulceration, Atherosclerosis of nonbiological bypass graft(s) of the extremities with gangrene, right leg, Atherosclerosis of nonbiological bypass graft(s) of the extremities with gangrene, left leg, Atherosclerosis of nonbiological bypass graft(s) of the extremities with gangrene, bilateral legs, Atherosclerosis of nonbiological bypass graft(s) of the extremities with gangrene, other extremity, Atherosclerosis of nonbiological bypass graft(s) of the extremities with gangrene, unspecified extremity, Other atherosclerosis of nonbiological bypass graft(s) of the extremities, right leg, Other atherosclerosis of nonbiological bypass graft(s) of the extremities, left leg, Other atherosclerosis of nonbiological bypass graft(s) of the extremities, bilateral legs, Other atherosclerosis of nonbiological bypass graft(s) of the extremities, other extremity, Other atherosclerosis of nonbiological bypass graft(s) of the extremities, unspecified extremity, Unspecified atherosclerosis of other type of bypass graft(s) of the extremities, right leg, Unspecified atherosclerosis of other type of bypass graft(s) of the extremities, left leg, Unspecified atherosclerosis of other type of bypass graft(s) of the extremities, bilateral legs, Unspecified atherosclerosis of other type of bypass graft(s) of the extremities, other extremity, Unspecified atherosclerosis of other type of bypass graft(s) of the extremities, unspecified extremity, Atherosclerosis of other type of bypass graft(s) of the extremities with intermittent claudication, right leg, Atherosclerosis of other type of bypass graft(s) of the extremities with intermittent claudication, left leg, Atherosclerosis of other type of bypass graft(s) of the extremities with intermittent claudication, bilateral legs, Atherosclerosis of other type of bypass graft(s) of the extremities with intermittent claudication, other extremity, Atherosclerosis of other type of bypass graft(s) of the extremities with intermittent claudication, unspecified extremity, Atherosclerosis of other type of bypass graft(s) of the extremities with rest pain, right leg, Atherosclerosis of other type of bypass graft(s) of the extremities with rest pain, left leg, Atherosclerosis of other type of bypass graft(s) of the extremities with rest pain, bilateral legs, Atherosclerosis of other type of bypass graft(s) of the extremities with rest pain, other extremity, Atherosclerosis of other type of bypass graft(s) of the extremities with rest pain, unspecified extremity, Atherosclerosis of other type of bypass graft(s) of the right leg with ulceration of thigh, Atherosclerosis of other type of bypass graft(s) of the right leg with ulceration of calf, Atherosclerosis of other type of bypass graft(s) of the right leg with ulceration of ankle, Atherosclerosis of other type of bypass graft(s) of the right leg with ulceration of heel and midfoot, Atherosclerosis of other type of bypass graft(s) of the right leg with ulceration of other part of foot, Atherosclerosis of other type of bypass graft(s) of the right leg with ulceration of other part of lower leg, Atherosclerosis of other type of bypass graft(s) of the right leg with ulceration of unspecified site, Atherosclerosis of other type of bypass graft(s) of the left leg with ulceration of thigh, Atherosclerosis of other type of bypass graft(s) of the left leg with ulceration of calf, Atherosclerosis of other type of bypass graft(s) of the left leg with ulceration of ankle, Atherosclerosis of other type of bypass graft(s) of the left leg with ulceration of heel and midfoot, Atherosclerosis of other type of bypass graft(s) of the left leg with ulceration of other part of foot, Atherosclerosis of other type of bypass graft(s) of the left leg with ulceration of other part of lower leg, Atherosclerosis of other type of bypass graft(s) of the left leg with ulceration of unspecified site, Atherosclerosis of other type of bypass graft(s) of other extremity with ulceration, Atherosclerosis of other type of bypass graft(s) of the extremities with gangrene, right leg, Atherosclerosis of other type of bypass graft(s) of the extremities with gangrene, left leg, Atherosclerosis of other type of bypass graft(s) of the extremities with gangrene, bilateral legs, Atherosclerosis of other type of bypass graft(s) of the extremities with gangrene, other extremity, Atherosclerosis of other type of bypass graft(s) of the extremities with gangrene, unspecified extremity, Other atherosclerosis of other type of bypass graft(s) of the extremities, right leg, Other atherosclerosis of other type of bypass graft(s) of the extremities, left leg, Other atherosclerosis of other type of bypass graft(s) of the extremities, bilateral legs, Other atherosclerosis of other type of bypass graft(s) of the extremities, other extremity, Other atherosclerosis of other type of bypass graft(s) of the extremities, unspecified extremity, Atherosclerosis of other arteries , Unspecified atherosclerosis , Generalized Atherosclerosis, Chronic total occlusion of artery of the extremities, Aneurysm of carotid artery, Aneurysm of artery of upper extremity, Aneurysm of renal artery, Aneurysm of iliac artery, Aneurysm of artery of lower extremity, Aneurysm of other specified arteries , Aneurysm of unspecified site, Other specified peripheral vascular diseases, Peripheral vascular disease, unspecified, Dissection of carotid artery, Dissection of iliac artery, Dissection of renal artery, Dissection of vertebral artery, Dissection of other artery, Other disorders of arteries, arterioles and capillaries in diseases classified elsewhere , Peripheral vascular angioplasty status with implants and grafts, Presence of other vascular implants and grafts |
| Peripheral Artery Disease | CPT | 33877, 34151, 34151, 34151, 34201, 34808, 34820, 34825, 34826, 35081, 35102, 35131, 35132, 35141, 35142, 35450, 35450, 35452, 35454, 35471, 35472, 35473, 35480, 35490, 35537, 35538, 35540, 35548, 35549, 35563, 35566, 35621, 35637, 35638, 35646, 35647, 35651, 35654, 35656, 35661, 35663, 35665, 35666 | Repair of thoracoabdominal aortic aneurysm with graft, with or without cardiopulmonary bypass, Embolectomy or thrombectomy, with or without catheter; renal, celiac, mesentery, aortoiliac artery, by abdominal incision, Embolectomy or thrombectomy, with or without catheter; renal, celiac, mesentery, aortoiliac artery, by abdominal incision, Embolectomy or thrombectomy, with or without catheter; renal, celiac, mesentery, aortoiliac artery, by abdominal incision, Embolectomy or thrombectomy, with or without catheter; femoropopliteal, aortoiliac artery, by leg incision, Endovascular placement of iliac artery occlusion device (list separately in addition to code for primary procedure), Open iliac artery exposure for delivery of endovascular prosthesis or iliac occlusion during endovascular therapy, by abdominal or retroperitoneal incision, unilateral, Placement of proximal or distal extension prosthesis for endovascular repair of infrarenal abdominal aortic or iliac aneurysm, false aneurysm, or dissection; initial vessel, Placement of proximal or distal extension prosthesis for endovascular repair of infrarenal abdominal aortic or iliac aneurysm, false aneurysm, or dissection; each additional vessel (list separately in addition to code for primary procedure), Direct repair of aneurysm, pseudoaneurysm, or excision (partial or total) and graft insertion, with or without patch graft; for aneurysm, pseudoaneurysm, and associated occlusive disease, abdominal aorta, Direct repair of aneurysm, pseudoaneurysm, or excision (partial or total) and graft insertion, with or without patch graft; for aneurysm, pseudoaneurysm, and associated occlusive disease, abdominal aorta involving iliac vessels (common, hypogastric, external), Direct repair of aneurysm, pseudoaneurysm, or excision (partial or total) and graft insertion, with or without patch graft; for aneurysm, pseudoaneurysm, and associated occlusive disease, iliac artery (common, hypogastric, external), Direct repair of aneurysm, pseudoaneurysm, or excision (partial or total) and graft insertion, with or without patch graft; for ruptured aneurysm, iliac artery (common, hypogastric, external), Direct repair of aneurysm, pseudoaneurysm, or excision (partial or total) and graft insertion, with or without patch graft; for aneurysm, pseudoaneurysm, and associated occlusive disease, common femoral artery (profunda femoris, superficial femoral), Direct repair of aneurysm, pseudoaneurysm, or excision (partial or total) and graft insertion, with or without patch graft; for ruptured aneurysm, common femoral artery (profunda femoris, superficial femoral), Transluminal balloon angioplasty, open; renal or other visceral artery, Transluminal balloon angioplasty, open; renal or other visceral artery, Transluminal balloon angioplasty, open; aortic, Transluminal balloon angioplasty, open; iliac, Transluminal balloon angioplasty, percutaneous; renal or visceral artery, Transluminal balloon angioplasty, percutaneous; aortic, Transluminal balloon angioplasty, percutaneous; iliac, Transluminal peripheral atherectomy, open; renal or other visceral artery, Transluminal peripheral atherectomy, percutaneous; renal or other visceral artery, Bypass graft, with vein; aortoiliac, Bypass graft, with vein; aortobi-iliac, Bypass graft, with vein; aortobifemoral, Bypass graft, with vein; aortoiliofemoral, unilateral, Bypass graft, with vein; aortoiliofemoral, bilateral, Bypass graft, with vein; ilioiliac, Bypass graft, with vein; femoral-anterior tibial, posterior tibial, peroneal artery or other distal vessels, Bypass graft, with other than vein; axillary-femoral, Bypass graft, with other than vein; aortoiliac, Bypass graft, with other than vein; aortobi-iliac, Bypass graft, with other than vein; aortobifemoral, Bypass graft, with other than vein; aortofemoral, Bypass graft, with other than vein; aortofemoral-popliteal, Bypass graft, with other than vein; axillary-femoral-femoral, Bypass graft, with other than vein; femoral-popliteal, Bypass graft, with other than vein; femoral-femoral, Bypass graft, with other than vein; ilioiliac, Bypass graft, with other than vein; iliofemoral, Bypass graft, with other than vein; femoral-anterior tibial, posterior tibial, or peroneal artery |
| Systemic Atherosclerosis | ICD9 | 441, 441.0, 441.00, 441.01, 441.02, 441.03, 441.1, 441.2, 441.3, 441.4, 441.5, 441.6, 441.7, 441.9 | Aortic aneurysm and dissection,  Dissection of aorta, thoracic, Dissection of aorta, abdominal, Dissection of aorta, thoracoabdominal, Thoracic aneurysm, ruptured, Thoracic aneurysm without mention of rupture, Abdominal aneurysm, ruptured, Abdominal aneurysm without mention of rupture, Aortic aneurysm of unspecified site, ruptured, Thoracoabdominal aneurysm, ruptured, Thoracoabdominal aneurysm, without mention of rupture, Aortic aneurysm of unspecified site without mention of rupture |
| Systemic Atherosclerosis | ICD10 | I70.0, I71.00, I71.01, I71.02, I71.03, I71.1, I71.2, I71.3, I71.4, I71.5, I71.6, I71.8, I71.9 | Atherosclerosis of aorta, Dissection of unspecified site of aorta, Dissection of thoracic aorta, Dissection of abdominal aorta, Dissection of thoracoabdominal aorta, Thoracic aortic aneurysm, ruptured , Thoracic aortic aneurysm, without rupture, Abdominal aortic aneurysm, ruptured , Abdominal aortic aneurysm, without rupture, Thoracoabdominal aortic aneurysm, ruptured , Thoracoabdominal aortic aneurysm, without rupture, Aortic aneurysm of unspecified site, ruptured, Aortic aneurysm of unspecified site, without rupture |
| Cerebral Atherosclerosis | ICD9 | 433, 433.0, 433.00, 433.01, 433.1, 433.11, 433.2, 433.20, 433.21, 433.3, 433.30, 433.31, 433.8, 433.80, 433.81, 433.91, 434.9, 434.90, 434.91, 435, 435.0, 435.1, 435.2, 435.3, 437, 437.1, 438.13, 438.14 | Occlusion and stenosis of basilar artery without mention of cerebral infarction, Occlusion and stenosis of basilar artery with cerebral infarction, Occlusion and stenosis of carotid artery without mention of cerebral infarction, Occlusion and stenosis of carotid artery with cerebral infarction, Occlusion and stenosis of vertebral artery without mention of cerebral infarction, Occlusion and stenosis of vertebral artery with cerebral infarction, Occlusion and stenosis of multiple and bilateral precerebral arteries without mention of cerebral infarction, Occlusion and stenosis of multiple and bilateral precerebral arteries with cerebral infarction, Occlusion and stenosis of other specified precerebral artery without mention of cerebral infarction,  Occlusion and stenosis of other specified precerebral artery with cerebral infarction, Occlusion and stenosis of unspecified precerebral artery with cerebral infarction, Cerebral artery occlusion, unspecified without mention of cerebral infarction, Cerebral artery occlusion, unspecified with cerebral infarction, Basilar artery syndrome, Vertebral artery syndrome, Subclavian steal syndrome, Vertebrobasilar artery syndrome, Cerebral atherosclerosis, Other generalized ischemic cerebrovascular disease, Late effects of cerebrovascular disease, dysarthria, Late effects of cerebrovascular disease, fluency disorder |
| Cerebral Atherosclerosis | ICD10 | G45.0, G45.8, I63.00, I63.011, I63.012, I63.019, I63.111, I63.112, I63.119, I63.12, I63.131, I63.132, I63.139, I63.19, I63.20, I63.211, I63.212, I63.219, I63.22, I63.231, I63.232, I63.239, I63.29, I63.30, I63.311, I63.312, I63.319, I63.321, I63.322, I63.329, I63.331, I63.332, I63.339, I63.341, I63.342, I63.349, I63.40, I63.411, I63.412, I63.419, I63.421, I63.422, I63.429, I63.431, I63.432, I63.439, I63.49, I63.50, I63.511, I63.512, I63.519, I63.521, I63.522, I63.529, I63.531, I63.532, I63.539, I63.541, I63.542, I63.549, I63.59, I65.01, I65.02, I65.03, I65.09, I65.1, I65.21, I65.22, I65.23, I65.29, I65.8, I65.9, I66.9, I67.2, I67.81, I67.82, I67.89 | Vertebro-basilar artery syndrome, Other Transient Cerebral Ischemic Attacks and related syndromes, Cerebral infarction due to thrombosis of unspecified precerebral artery, Cerebral infarction due to thrombosis of right vertebral artery, Cerebral infarction due to thrombosis of left vertebral artery, Cerebral infarction due to thrombosis of unspecified vertebral artery, Cerebral infarction due to embolism of right vertebral artery, Cerebral infarction due to embolism of left vertebral artery, Cerebral infarction due to embolism of unspecified vertebral artery , Cerebral infarction due to embolism of basilar artery, Cerebral infarction due to embolism of right carotid artery, Cerebral infarction due to embolism of left carotid artery, Cerebral infarction due to embolism of unspecified carotid artery, Cerebral infarction due to embolism of other precerebral artery, Cerebral infarction due to unspecified occlusion or stenosis of unspecified precerebral arteries , Cerebral infarction due to unspecified occlusion or stenosis of right vertebral arteries, Cerebral infarction due to unspecified occlusion or stenosis of left vertebral arteries, Cerebral infarction due to unspecified occlusion or stenosis of unspecified vertebral arteries, Cerebral Infraction Due to Unspecified Occlusion or Stenosis of Basilar Arteries, Cerebral infarction due to unspecified occlusion or stenosis of right carotid arteries, Cerebral infarction due to unspecified occlusion or stenosis of left carotid arteries, Cerebral infarction due to unspecified occlusion or stenosis of unspecified carotid arteries, Cerebral infarction due to unspecified occlusion or stenosis of other precerebral arteries, Cerebral infarction due to thrombosis of unspecified cerebral artery , Cerebral infarction due to thrombosis of right middle cerebral artery, Cerebral infarction due to thrombosis of left middle cerebral artery, Cerebral infarction due to thrombosis of unspecified middle cerebral artery, Cerebral infarction due to thrombosis of right anterior cerebral artery, Cerebral infarction due to thrombosis of left anterior cerebral artery, Cerebral infarction due to thrombosis of unspecified anterior cerebral artery, Cerebral infarction due to thrombosis of right posterior cerebral artery, Cerebral infarction due to thrombosis of left posterior cerebral artery, Cerebral infarction due to thrombosis of unspecified posterior cerebral artery, Cerebral infarction due to thrombosis of right cerebellar artery, Cerebral infarction due to thrombosis of left cerebellar artery, Cerebral infarction due to thrombosis of unspecified cerebellar artery, Cerebral infarction due to embolism of unspecified cerebral artery , Cerebral infarction due to embolism of right middle cerebral artery, Cerebral infarction due to embolism of left middle cerebral artery, Cerebral infarction due to embolism of unspecified middle cerebral artery, Cerebral infarction due to embolism of right anterior cerebral artery, Cerebral infarction due to embolism of left anterior cerebral artery, Cerebral infarction due to embolism of unspecified anterior cerebral artery, Cerebral infarction due to embolism of right posterior cerebral artery, Cerebral infarction due to embolism of left posterior cerebral artery, Cerebral infarction due to embolism of unspecified posterior cerebral artery , Cerebral infarction due to embolism of other cerebral artery, Cerebral infarction due to unspecified occlusion or stenosis of unspecified cerebral artery , Cerebral infarction due to unspecified occlusion or stenosis of right middle cerebral artery, Cerebral infarction due to unspecified occlusion or stenosis of left middle cerebral artery, Cerebral infarction due to unspecified occlusion or stenosis of unspecified middle cerebral artery, Cerebral infarction due to unspecified occlusion or stenosis of right anterior cerebral artery, Cerebral infarction due to unspecified occlusion or stenosis of left anterior cerebral artery, Cerebral infarction due to unspecified occlusion or stenosis of unspecified anterior cerebral artery, Cerebral infarction due to unspecified occlusion or stenosis of right posterior cerebral artery, Cerebral infarction due to unspecified occlusion or stenosis of left posterior cerebral artery, Cerebral infarction due to unspecified occlusion or stenosis of unspecified posterior cerebral artery, Cerebral infarction due to unspecified occlusion or stenosis of right cerebellar artery, Cerebral infarction due to unspecified occlusion or stenosis of left cerebellar artery, Cerebral infarction due to unspecified occlusion or stenosis of unspecified cerebellar artery, Cerebral infarction due to unspecified occlusion or stenosis of other cerebral artery , Occlusion and stenosis of right vertebral artery, Occlusion and stenosis of left vertebral artery , Occlusion and stenosis of bilateral vertebral arteries, Occlusion and stenosis of unspecified vertebral artery , Occlusion and stenosis of basilar artery , Occlusion and stenosis of right carotid artery, Occlusion and stenosis of left carotid artery, Occlusion and stenosis of bilateral carotid arteries, Occlusion and stenosis of unspecified carotid artery , Occlusion and stenosis of other precerebral arteries , Occlusion and stenosis of unspecified precerebral artery, Occlusion and stenosis of unspecified cerebral artery, Cerebral atherosclerosis, Acute cerebrovascular insufficiency , Cerebral ischemia , Other cerebrovascular disease |
| Smoking | ICD9 | 305.1, 989.84, V15.82 | Tobacco use disorder, Toxic effect of tobacco, Personal history of tobacco use |
| Smoking | ICD10 | F17.200, F17.220, F17.221, F17.223, F17.228, F17.229, F17.290, F17.291, F17.293, F17.298, F17.299, T65.211A, T65.211D, T65.211S, T65.212A, T65.212D, T65.212S, T65.213A, T65.213D, T65.213S, T65.214A, T65.214D, T65.214S, T65.221A, T65.221D, T65.221S, T65.222A, T65.222D, T65.222S, T65.223A, T65.223D, T65.223S, T65.224A, T65.224D, T65.224S, T65.291A, T65.291D, T65.291S, T65.292A, T65.292D, T65.292S, T65.293A, T65.293D, T65.293S, T65.294A, T65.294D, T65.294S, Z71.6, Z72.0, Z87.891 | Nicotine dependence, unspecified, uncomplicated, Nicotine dependence, chewing tobacco, uncomplicated, Nicotine dependence, chewing tobacco, in remission, Nicotine dependence, chewing tobacco, with withdrawal, Nicotine dependence, chewing tobacco, with other nicotine-induced disorders, Nicotine dependence, chewing tobacco, with unspecified nicotine-induced disorders, Nicotine dependence, other tobacco produce, uncomplicated, Nicotine dependence, other tobacco produce, in remission, Nicotine dependence, other tobacco produce, with withdrawal, Nicotine dependence, other tobacco produce, with other nicotine-induced disorders, Nicotine dependence, other tobacco produce, with unspecified nicotine-induced disorder, Toxic effect of chewing tobacco, accidental (unintentional), initial encounter, Toxic effect of chewing tobacco, accidental (unintentional), subsequent encounter, Toxic effect of chewing tobacco, accidental (unintentional), sequela, Toxic effect of chewing tobacco, intentional self-harm, initial encounter, Toxic effect of chewing tobacco, intentional self-harm, subsequent encounter, Toxic effect of chewing tobacco, intentional self-harm, sequela, Toxic effect of chewing tobacco, assault, initial encounter, Toxic effect of chewing tobacco, assault, subsequent encounter, Toxic effect of chewing tobacco, assault, sequela, Toxic effect of chewing tobacco, undetermined, initial encounter, Toxic effect of chewing tobacco, undetermined, subsequent encounter, Toxic effect of chewing tobacco, undetermined, sequela, Toxic effect of tobacco cigarettes, accidental (unintentional), initial encounter, Toxic effect of tobacco cigarettes, accidental (unintentional), subsequent encounter, Toxic effect of tobacco cigarettes, accidental (unintentional), sequela, Toxic effect of tobacco cigarettes, intentional self-harm, initial encounter , Toxic effect of tobacco cigarettes, intentional self-harm, subsequent encounter , Toxic effect of tobacco cigarettes, intentional self-harm, sequela, Toxic effect of tobacco cigarettes, assault, initial encounter , Toxic effect of tobacco cigarettes, assault, subsequent encounter , Toxic effect of tobacco cigarettes, assault, sequela, Toxic effect of tobacco cigarettes, undetermined, initial encounter , Toxic effect of tobacco cigarettes, undetermined, subsequent encounter , Toxic effect of tobacco cigarettes, undetermined, sequela, Toxic effect of other tobacco and nicotine, accidental (unintentional), initial encounter, Toxic effect of other tobacco and nicotine, accidental (unintentional), subsequent encounter, Toxic effect of other tobacco and nicotine, accidental (unintentional), sequela, Toxic effect of other tobacco and nicotine, intentional self-harm, initial encounter , Toxic effect of other tobacco and nicotine, intentional self-harm, subsequent encounter , Toxic effect of other tobacco and nicotine, intentional self-harm, sequela, Toxic effect of other tobacco and nicotine, assault, initial encounter , Toxic effect of other tobacco and nicotine, assault, subsequent encounter , Toxic effect of other tobacco and nicotine, assault, sequela, Toxic effect of other tobacco and nicotine, undetermined, initial encounter, Toxic effect of other tobacco and nicotine, undetermined, subsequent encounter, Toxic effect of other tobacco and nicotine, undetermined, sequela, Tobacco abuse counseling, Tobacco abuse , Personal history of nicotine dependence |
| Smoking | Med | buproban, bupropion, chantix, commit, habitrol, nicoderm, nicorelief, nicorette, nicotine polacrilex, nicotrol, varenicline, zyban | Pulmonary embolism, Phlebitis and thrombophlebitis of deep vessels of lower extremity |
| Chronic Kidney Disease | ICD9 | 250.4, 250.40, 250.41, 250.42, 250.43, 403, 403.01, 403.1, 403.10, 403.11, 403.9, 403.90, 403.91, 404, 404.0, 404.00, 404.01, 404.02, 404.03, 404.1, 404.10, 404.11, 404.12, 404.13, 404.9, 404.90, 404.91, 404.92, 404.93, 582, 582.0, 582.1, 582.2, 582.4, 582.81, 582.89, 582.9, 583, 583.0, 583.1, 583.2, 583.4, 583.6, 583.7, 583.81, 583.89, 583.9, 584, 584.5, 584.6, 584.7, 584.8, 584.9, 585, 585.1, 585.2, 585.3, 586, 587, 588, 588.0, 588.81, 588.89, 588.9, 753, 753.0, 753.12, 753.13, 753.14, 753.15, 753.16, 753.17, 753.19, 788.5, 792.5, V42.0, V45.11, V45.12, V56.0, V56.1, V56.2, V56.31, V56.32, V56.8 | Diabetes with renal manifestations, Diabetes with renal manifestations, type I [juvenile type], not stated as uncontrolled, Diabetes with renal manifestations, type II or unspecified type, uncontrolled, Diabetes with renal manifestations, type I [juvenile type], uncontrolled, Hypertensive chronic disease, Hypertensive chronic kidney disease, malignant, with chronic kidney disease stage V or end stage renal disease, Benign hypertensive kidney disease, Hypertensive chronic kidney disease, benign, with chronic kidney disease stage V or end stage renal disease, Unspecified hypertensive kidney disease, Hypertensive chronic kidney disease, unspecified, with chronic kidney disease stage V or end stage renal disease, Hypertensive heart and chronic disease, Hypertensive heart and chronic kidney disease, malignant, with heart failure and with chronic kidney disease stage I through stage IV, or unspecified, Hypertensive heart and chronic kidney disease, malignant, without heart failure and with chronic kidney disease stage V or end stage renal disease, Hypertensive heart and chronic kidney disease, malignant, with heart failure and with chronic kidney disease stage V or end stage renal disease, Benign hypertensive heart and kidney disease, Hypertensive heart and chronic kidney disease, benign, with heart failure and with chronic kidney disease stage I through stage IV, or unspecified,  Hypertensive heart and chronic kidney disease, benign, without heart failure and with chronic kidney disease stage V or end stage renal disease, Hypertensive heart and chronic kidney disease, benign, with heart failure and chronic kidney disease stage V or end stage renal disease, Unspecified hypertensive heart and kidney disease,  Hypertensive heart and chronic kidney disease, unspecified, with heart failure and with chronic kidney disease stage I through stage IV, or unspecified, Hypertensive heart and chronic kidney disease, unspecified, without heart failure and with chronic kidney disease stage V or end stage renal disease, Hypertensive heart and chronic kidney disease, unspecified, with heart failure and chronic kidney disease stage V or end stage renal disease, Chronic glomerulonephritis, Chronic glomerulonephritis with lesion of membranous glomerulonephritis, Chronic glomerulonephritis with lesion of membranoproliferative glomerulonephritis, Chronic glomerulonephritis with lesion of rapidly progressive glomerulonephritis, Chronic glomerulonephritis in diseases classified elsewhere, Chronic glomerulonephritis with other specified pathological lesion in kidney, Chronic glomerulonephritis with unspecified pathological lesion in kidney, Nephritis and nephropathy, not specified as acute or chronic, Nephritis and nephropathy, not specified as acute or chronic, with lesion of membranous glomerulonephritis, Nephritis and nephropathy, not specified as acute or chronic, with lesion of membranoproliferative glomerulonephritis, Nephritis and nephropathy, not specified as acute or chronic, with lesion of rapidly progressive glomerulonephritis,  Nephritis and nephropathy, not specified as acute or chronic, with lesion of renal cortical necrosis, Nephritis and nephropathy, not specified as acute or chronic, with lesion of renal medullary necrosis, Nephritis and nephropathy, not specified as acute or chronic, in diseases classified elsewhere, Nephritis and nephropathy, not specified as acute or chronic, with other specified pathological lesion in kidney, Nephritis and nephropathy, not specified as acute or chronic, with unspecified pathological lesion in kidney, Acute kidney failure, Acute kidney failure with lesion of tubular necrosis, Acute kidney failure with lesion of renal cortical necrosis,  Acute kidney failure with lesion of renal medullary [papillary] necrosis, Acute kidney failure with other specified pathological lesion in kidney, Acute kidney failure, unspecified, Chronic kidney disease, Stage I, Chronic kidney disease, Stage II (mild), Chronic kidney disease, Stage III (moderate), Renal failure, unspecified, Renal sclerosis, unspecified, Disorders resulting from impaired renal function, Secondary hyperparathyroidism (of renal origin), Other specified disorders resulting from impaired renal function, Unspecified disorder resulting from impaired renal function, Renal agenesis, Polycystic kidney, unspecified type, Polycystic kidney, autosomal dominant,  Polycystic kidney, autosomal recessive, Renal dysplasia, Medullary cystic kidney, Medullary sponge kidney, Other specified cystic kidney disease, Oliguria and anuria, Cloudy (hemodialysis) (peritoneal) dialysis effluent, Kidney replaced by transplant,  Renal dialysis status, Noncompliance with renal dialysis, Encounter for extracorporeal dialysis, Fitting and adjustment of extracorporeal dialysis catheter, Fitting and adjustment of peritoneal dialysis catheter, Encounter for adequacy testing for hemodialysis, encounter for adequacy testing for peritoneal dialysis, Encounter for other dialysis |
| Chronic Kidney Disease | ICD10 | E08.22, E09.22, E10.22, E11.22, E13.22, I12.0, I12.9, I13.0, I13.1, I13.10, I13.11, I13.2, N18.1, N18.2, N18.3, N18.4, N18.5, N18.9, N19, N99.0, R34, Z49.01, Z49.02, Z49.31, Z49.32, Z99.2 | Diabetes mellitus due to underlying condition with diabetic chronic kidney disease, Drug or chemical induced diabetes mellitus with diabetic chronic kidney disease, Type 1 diabetes mellitus with diabetic chronic kidney disease, Type 2 diabetes mellitus with diabetic chronic kidney disease, Other specified diabetes mellitus with diabetic chronic kidney disease, Hypertensive Chronic Kidney Disease With Stage 5 Chronic Kidney Disease Or End Stage Renal Disease, Hypertensive Chronic Kidney Disease With Stage 1 Through Stage 4 Chronic Kidney Disease, Or Unspecified Chronic Kidney Disease, Hypertensive Heart And Chronic Kidney Disease With Heart Failure And Stage 1 Through Stage 4 Chronic Kidney Disease, Or Unspecified Chronic Kidney Disease, Hypertensive heart and chronic kidney disease without heart failure, Hypertensive Heart And Chronic Kidney Disease Without Heart Failure, With Stage 1 Through Stage 4 Chronic Kidney Disease, Or Unspecified Chronic Kidney Disease, Hypertensive Heart And Chronic Kidney Disease Without Heart Failure, With Stage 5 Chronic Kidney Disease, Or End Stage Renal Disease, Hypertensive Heart And Chronic Kidney Disease With Heart Failure And With Stage 5 Chronic Kidney Disease, Or End Stage Renal Disease, Chronic Kidney Disease, Stage 1, Chronic Kidney Disease, Stage 2 (Mild), Chronic Kidney Disease, Stage 3 (Moderate), Chronic kidney disease, stage 4 (severe), Chronic kidney disease, stage 5, Chronic kidney disease, unspecified, Unspecified Kidney Failure, Postprocedural (acute)(chronic) kidney failure, Anuria And Oliguria, Encounter For Fitting And Adjustment Of Extracorporeal Dialysis Catheter, Encounter For Fitting And Adjustment Of Peritoneal Dialysis Catheter, Encounter For Adequacy Testing For Hemodialysis, Encounter For Adequacy Testing For Peritoneal Dialysis, Dependence On Renal Dialysis |
| Chronic Kidney Disease | CPT | 36147, 36148, 36818, 36819, 36820, 36821, 36825, 36830, 36831, 36832, 36833, 36870, 50360, 50365, 75791, 90935, 90937, 90945, 90947, 90951, 90952, 90953, 90954, 90955, 90956, 90957, 90958, 90959, 90960, 90961, 90962, 90963, 90964, 90965, 90966, 90967, 90968, 90969, 90970 | Access Av Dial Grft For Eval, Access Av Dial Grft For Proc, Av Fuse Uppr Arm Cephalic, Av Fuse Uppr Arm Basilic, Av Fusion/Forearm Vein, Av Fusion Direct Any Site, Artery-Vein Autograft, Artery-Vein Nonautograft, Open Thrombect Av Fistula, Av Fistula Revision Open, Av Fistula Revision, Percut Thrombect Av Fistula, Transplantation Of Kidney, Transplantation Of Kidney, Av Dialysis Shunt Imaging, Hemodialysis One Evaluation, Hemodialysis Repeated Eval, Dialysis One Evaluation, Dialysis Repeated Eval, Esrd Serv 4 Visits P Mo <2Yr, Esrd Serv 2-3 Vsts P Mo <2Yr, Esrd Serv 1 Visit P Mo <2Yrs, Esrd Serv 4 Vsts P Mo 2-11, Esrd Srv 2-3 Vsts P Mo 2-11, Esrd Srv 1 Visit P Mo 2-11, Esrd Srv 4 Vsts P Mo 12-19, Esrd Srv 2-3 Vsts P Mo 12-19, Esrd Serv 1 Vst P Mo 12-19, Esrd Srv 4 Visits P Mo 20+, Esrd Srv 2-3 Vsts P Mo 20+, Esrd Serv 1 Visit P Mo 20+, Esrd Home Pt Serv P Mo <2Yrs, Esrd Home Pt Serv P Mo 2-11, Esrd Home Pt Serv P Mo 12-19, Esrd Home Pt Serv P Mo 20+, Esrd Home Pt Serv P Day <2, Esrd Home Pt Srv P Day 2-11, Esrd Home Pt Srv P Day 12-19, Esrd Home Pt Serv P Day 20+ |
| Thyrotoxicosis | ICD9 | 242, 242.0, 242.00, 242.01, 242.1, 242.10, 242.11, 242.2, 242.20, 242.21, 242.3, 242.30, 242.31, 242.4, 242.40, 242.41, 242.8, 242.80, 242.81, 242.9, 242.90, 242.91 | Thyrotoxicosis with or without goiter, Toxic diffuse goiter with mention of thyrotoxic crisis or storm, Toxic uninodular goiter, Toxic uninodular goiter with mention of thyrotoxic crisis or storm, Toxic diffuse goiter without mention of thyrotoxic crisis or storm, Toxic multinodular goiter with mention of thyrotoxic crisis or storm, Toxic nodular goiter, unspecified,  Toxic nodular goiter, unspecified type, with mention of thyrotoxic crisis or storm, Thyrotoxicosis from ectopic thyroid nodule, Thyrotoxicosis from ectopic thyroid nodule with mention of thyrotoxic crisis or storm, Thyrotoxicosis of other specified origin, Thyrotoxicosis of other specified origin with mention of thyrotoxic crisis or storm, Thyrotoxicosis without mention of goiter or other cause, Thyrotoxicosis without mention of goiter or other cause, with mention of thyrotoxic crisis or storm |
| Thyrotoxicosis | ICD10 | E05.00, E05.01, E05.10, E05.11, E05.20, E05.21, E05.30, E05.31, E05.40, E05.41, E05.80, E05.81, E05.90, E05.91 | Thyrotoxicosis with diffuse goiter without thyrotoxic crisis or storm , Thyrotoxicosis with diffuse goiter with thyrotoxic crisis or storm , Thyrotoxicosis with toxic single thyroid nodule without thyrotoxic crisis or storm , Thyrotoxicosis with toxic single thyroid nodule with thyrotoxic crisis or storm , Thyrotoxicosis with toxic multinodular goiter without thyrotoxic crisis or storm , Thyrotoxicosis with toxic multinodular goiter with thyrotoxic crisis or storm , Thyrotoxicosis from ectopic thyroid tissue without thyrotoxic crisis or storm , Thyrotoxicosis from ectopic thyroid tissue with thyrotoxic crisis or storm , Thyrotoxicosis factitia without thyrotoxic crisis or storm , Thyrotoxicosis factitia with thyrotoxic crisis or storm , Other thyrotoxicosis without thyrotoxic crisis or storm , Other thyrotoxicosis with thyrotoxic crisis or storm , Thyrotoxicosis, unspecified without thyrotoxic crisis or storm , Thyrotoxicosis, unspecified with thyrotoxic crisis or storm |
| Thyrotoxicosis | Med | iopanoic acid, methimazole, propylthiouracil, tapazole |  |
| Hypothyroidism | ICD9 | 243, 244, 244.0, 244.1, 244.8, 244.9, 245, 245.0, 245.1, 245.2, 245.9 | Congenital hypothyroidism, Postsurgical hypothyroidism, Hypothyroidism, post-ablative, Hypothyroidism, secondary to pituitary disease, Primary hypothyroidism, Thyroiditis, acute, Thyroiditis, subacute, Thyroiditis, chronic, Hashimoto's, Thyroiditis |
| Hypothyroidism | ICD10 | E03.1, E03.8, E03.9, E06.1, E06.3, E06.5, E06.9, E89.0 | Congenital hypothyroidism, Hypothyroidism, secondary to pituitary disease, Primary hypothyroidism, Subacute thyroiditis, Autoimmune thyroid disorder, Chronic lymphocytic thyroiditis, Thyroiditis, Postsurgical hypothyroidism |
| Hypothyroidism | Med | L-thyroxin, levo-T, levolet, levothroid, levothyroxine sodium, levoxyl, novothyrox, synthroid, tirosint, unithroid |  |
| Pulmonary Disease | ICD9 | 490, 491, 491.0, 491.1, 491.20, 491.21, 491.22, 491.8, 491.9, 492.0, 492.8, 493.00, 493.01, 493.02, 493.10, 493.11, 493.12, 493.20, 493.21, 493.22, 493.81, 493.82, 493.90, 493.91, 493.92, 494, 494.0, 494.1, 495, 495.0, 495.1, 495.2, 495.3, 495.4, 495.5, 495.6, 495.7, 495.8, 495.9, 496, 500, 501, 502, 503, 504, 505, 506, 506.0, 506.1, 506.2, 506.3, 506.4, 506.9 | Bronchitis NOS, Simple chronic bronchitis, Mucopurulent chronic bronchitis, Obstructive chronic bronchitis without exacerbation, obstructive chronic bronchitis with acute exacerbation, obstructive chronic bronchitis with acute bronchitis, chronic bronchitis NEC, chronic bronchitis NOS, Emphysematous bleb, Emphysema NEC, Extrinsic asthma NOS, Extrinsic asthma with status asthmaticus, Extrinsic asthma with acute exacerbation, Intrinsic asthma NOS, Intrinsic asthma with status asthmaticus, intrinsic asthma with acute exacerbation, Chronic obstructive asthma NOS, Chronic obstructive asthma with status asthmaticus, Chronic obstructive asthma with acute exacerbation, Exercise induced bronchospasm, Cough variant asthma, Asthma NOS, Asthma with status asthmaticus, Asthma NOS with acute exacerbation, Bronchiectasis without acute exacerbation, Bronchiectasis with acute exacerbation, Farmer’s Lung, Bagassosis, Bird-Fanciers’ Lung, Suberosis, Malt workers’ Lung, Mushroom workers’ Lung, Maple bark-strippers’ lung, “ventilation” pneumonitis, Allergic alveol/pneum NEC, Allergic alveol/pneum NOS, Chronic airway obstruction, not elsewhere classified, Coal workers' pneumoconiosis, Asbestosis, Pneumoconiosis due to other silica or silicates, Pneumoconiosis due to other inorganic dust, Pneumonopathy due to inhalation of other dust, Pneumoconiosis, unspecified, Fum/vapor bronch/pneumon, Fum/vapor ac pulm edema, Fum/vapor up resp inflame, Fum/vapor ac resp cond NEC, Fum/vapor chr resp cond, Fum/vapor resp cond NOS |
| Pulmonary Disease | ICD10 | A15.0, A52.72, B38.1, B39.1, B40.1, D86.0, D86.2, E84.0, J40, J41.0, J41.1, J41.8, J42, J43.0, J43.1, J43.2, J43.8, J43.9, J44, J44.0, J44.1, J44.9, J45.20, J45.21, J45.22, J45.30, J45.31, J45.32, J45.40, J45.41, J45.42, J45.50, J45.51, J45.52, J45.901, J45.902, J45.909, J45.990, J45.991, J45.998, J47, J47.0, J47.1, J47.9, J60, J61, J62.0, J62.8, J63.0, J63.1, J63.2, J63.3, J63.4, J63.5, J63.6, J64, J65, J66.0, J66.1, J66.2, J66.8, J67.0, J67.2, J67.4, J67.5, J67.6, J67.7, J67.8, J67.9, J671, J673, J68.0, J68.1, J68.2, J68.3, J68.4, J68.9, J70.1, J70.3, J70.4, J81.8, J82, J84.02, J84.03, J84.10, J84.112, J84.115, J84.17, J84.82, J84.842, J84.89, J84.9, J95.3, J98.2, J98.3, M30.1, M32.13, M34.81, M35.02 | Tuberculosis of lung, Syphilis of lung and bronchus, Chronic pulmonary coccidioidomycosis, Chronic, pulmonary histoplasmosis capsulati, Chronic pulmonary blastomycosis, Sarcoidosis of lung, Sarcoidosis of lung with sarcoidosis of lymph nodes, Cystic fibrosis with pulmonary manifestations, Bronchitis, not specified as acute or chronic , Simple chronic bronchitis, Mucopurulent chronic bronchitis , Mixed simple and mucopurulent chronic bronchitis , Unspecified chronic bronchitis , Unilateral pulmonary emphysema (MacLeod's syndrome), Panlobular emphysema, Centrilobular emphysema, Other emphysema, Emphysema, unspecified, Other chronic obstructive pulmonary disease, Chronic obstructive pulmonary disease with acute lower respiratory infection , Chronic obstructive pulmonary disease with (acute) exacerbation , unspecified, Chronic obstructive pulmonary disease, unspecified , Mild intermittent asthma, uncomplicated , Mild intermittent asthma with (acute) exacerbation , Mild intermittent asthma with status asthmaticus, Mild persistent asthma, uncomplicated, Mild persistent asthma, with (acute) exacerbation, Mild persistent asthma, with status asthmaticus, Moderate persistent asthma, uncomplicated, Moderate persistent asthma, with (acute) exacerbation, Moderate persistent asthma, with status asthmaticus, Severe persistent asthma, uncomplicated, Severe persistent asthma, with (acute) exacerbation, Severe persistent asthma, with status asthmaticus, Unspecified asthma with (acute) exacerbation , Unspecified asthma with status asthmaticus , Unspecified asthma, uncomplicated , Exercise induced bronchospasm , Cough variant asthma , Other asthma , Bronchiectasis, Bronchiectasis with acute lower respiratory infection, Bronchiectasis with (acute) exacerbation , Bronchiectasis, uncomplicated , Coalworker's pneumoconiosis, Pneumoconiosis due to asbestos and other mineral fibers, Pneumoconiosis due to talc dust, Pneumoconiosis due to other dust containing silica , Aluminosis (of lung) , Bauxite fibrosis (of lung) , Berylliosis , Graphite fibrosis (of lung) , Siderosis , Stannosis , Pneumoconiosis due to other specified inorganic dusts , Unspecified Pneumoconiosis, Pneumoconiosis associated with tuberculosis, Byssinosis, Flax-Dressers Disease, Cannabinosis, Airway Diseases due to other specific organic dusts, Farmer's lung , Bird fancier's lung , Maltworker's lung , Mushroom-worker's lung , Maple-bark-stripper's lung , Air conditioner and humidifier lung , Hypersensitivity pneumonitis due to other organic dusts , Hypersensitivity pneumonitis due to unspecified organic dust , Bagassosis , Suberosis, Bronchitis and pneumonitis due to chemicals, gases, fumes and vapors , Pulmonary edema due to chemicals, gases, fumes and vapors , Upper respiratory inflammation due to chemicals, gases, fumes and vapors, not elsewhere classified , Other acute and subacute respiratory conditions due to chemicals, gases, fumes and vapors , Chronic respiratory conditions due to chemicals, gases, fumes and vapors , Unspecified respiratory condition due to chemicals, gases, fumes and vapors , Chronic and other pulmonary manifestations due to radiation, Chronic drug-induced interstitial lung disorders, Drug-induced interstitial lung disorders, unspecified, Chronic pulmonary edema, Pulmonary eosinophilia, not elsewhere classified, Pulmonary alveolar microlithiasis, Idiopathic pulmonary hemosiderosis, Pulmonary fibrosis, unspecified, Idiopathic pulmonary fibrosis, Respiratory bronchiolitis interstitial lung disease, Other interstitial pulmonary diseases with fibrosis in diseases classified elsewhere, Adult pulmonary Langerhans cell histiocytosis, Pulmonary interstitial glycogenosis, Other specified interstitial pulmonary diseases, Interstitial pulmonary disease, unspecified, Chronic pulmonary insufficiency following surgery, Interstitial emphysema, Compensatory emphysema, Polyarteritis with lung involvement (Churg-Strauss), Lung involvement in systemic lupus erythematosus, Systemic sclerosis with lung involvement, Sicca syndrome with lung involvement |

**Supplementary Table VIII. Cohort details for the 4 subpopulations used to analyze ASVCD incidence (rates per 1K PY).** Prev. = Prevalence; HF = Heart failure.

|  | | **Explorys** | | **MGB** | | **UKBB** | |
| --- | --- | --- | --- | --- | --- | --- | --- |
|  |  | **n** | **Incidence** | **n** | **Incidence** | **n** | **Incidence** |
| **Female-White** | **All** | 1763103 | 18.8 | 107998 | 6.2 | 219905 | 1.9 |
|  | **40–49** | 379750 | 7.0 | 30907 | 2.4 | 48065 | 0.6 |
|  | **50–59** | 513988 | 11.3 | 34970 | 4.0 | 74467 | 1.3 |
|  | **60–69** | 485185 | 20.4 | 27531 | 8.6 | 93342 | 3.0 |
|  | **70–79** | 384180 | 42.0 | 14590 | 18.2 | 4031 | 5.2 |
|  | **Prev. HF** | 48670 | 68.5 | 1195 | 37.0 | 397 | 7.6 |
| **Female-Black** | **All** | 281072 | 23.8 | 8450 | 11.2 | 3705 | 1.9 |
|  | **40–49** | 79377 | 12.3 | 3169 | 6.7 | 1565 | 1.2 |
|  | **50–59** | 91094 | 19.5 | 2822 | 9.3 | 1323 | 1.5 |
|  | **60–69** | 67742 | 28.9 | 1615 | 17.2 | 781 | 4.0 |
|  | **70–79** | 42859 | 50.9 | 844 | 27.5 | 36 | 3.4 |
|  | **Prev. HF** | 13889 | 71.4 | 206 | 61.8 | 12 | 10.4 |
| **Male-White** | **All** | 1434369 | 24.2 | 76304 | 11.0 | 181748 | 4.5 |
|  | **40–49** | 312314 | 8.9 | 22926 | 4.3 | 40861 | 1.7 |
|  | **50–59** | 433691 | 16.3 | 23906 | 9.0 | 57926 | 3.6 |
|  | **60–69** | 399729 | 27.9 | 19298 | 15.5 | 79192 | 6.5 |
|  | **70–79** | 288635 | 51.8 | 10174 | 26.8 | 3769 | 9.7 |
|  | **Prev. HF** | 52980 | 72.1 | 1563 | 36.7 | 820 | 15.1 |
| **Male-Black** | **All** | 178136 | 27.9 | 5432 | 12.8 | 2796 | 3.0 |
|  | **40–49** | 50424 | 14.5 | 2201 | 7.0 | 1274 | 1.5 |
|  | **50–59** | 60508 | 24.3 | 1840 | 13.1 | 927 | 2.3 |
|  | **60–69** | 43168 | 34.9 | 1014 | 21.3 | 551 | 7.0 |
|  | **70–79** | 24036 | 57.8 | 377 | 31.9 | 44 | 14.6 |
|  | **Prev. HF** | 11594 | 77.0 | 161 | 43.4 | 9 | 14.6 |

**Supplementary Table IX. Incidence rates per 1K PY and population sizes.** Prev. = Prevalence; HF = Heart failure. Refer to Supplementary Table VIII for additional PCE subpopulations.

|  |  | **Explorys** | | **MGB** | | **UKBB** | |
| --- | --- | --- | --- | --- | --- | --- | --- |
|  |  | **n** | **Incidence** | **n** | **Incidence** | **n** | **Incidence** |
| **CHARGE-AF** | **All** | 4750660 | 12.8 | 174644 | 12.1 | 445329 | 3.4 |
|  | **45–54** | 1314446 | 2.8 | 59480 | 4.3 | 138424 | 1.0 |
|  | **55–64** | 1446419 | 7.1 | 58541 | 8.7 | 204290 | 3.1 |
|  | **65–74** | 1163303 | 16.8 | 38167 | 18.6 | 102615 | 7.2 |
|  | **75–84** | 780025 | 37.4 | 15651 | 39.5 | - | |
|  | **85–90** | 46467 | 59.1 | 2805 | 61.2 |  |  |
|  | **Female-only** | 2695367 | 10.5 | 106279 | 9.4 | 244717 | 2.2 |
|  | **Male-only** | 2055293 | 16.0 | 68365 | 16.3 | 200612 | 4.9 |
|  | **Black-only** | 748819 | 8.5 | 33898 | 9.2 | 23450 | 2.0 |
|  | **White-only** | 4001841 | 13.5 | 140746 | 12.8 | 421879 | 3.5 |
|  | **Prev. Stroke** | 238438 | 28.4 | 3457 | 32.1 | 6819 | 11.4 |
|  | **Prev. HF** | 177769 | 57.1 | 3334 | 60.6 | 1828 | 21.5 |
| **PCE (Female-White)** | **All** | 1763103 | 18.8 | 107998 | 6.2 | 219905 | 1.9 |
|  | **40–49** | 379750 | 7.0 | 30907 | 2.4 | 48065 | 0.6 |
|  | **50–59** | 513988 | 11.3 | 34970 | 4.0 | 74467 | 1.3 |
|  | **60–69** | 485185 | 20.4 | 27531 | 8.6 | 93342 | 3.0 |
|  | **70–79** | 384180 | 42.0 | 14590 | 18.2 | 4031 | 5.2 |
|  | **Prev. HF** | 48670 | 68.5 | 1195 | 37.0 | 397 | 7.6 |

**Supplementary Table X. Acceptable discrimination and calibration indications for subgroups considering CHARGE-AF.** Concordance Index > 0.7 is considered as a good discrimination; calibration slope between 0.7 and 1.3 is considered good; green: both discrimination and calibration perform reasonably well; orange: discrimination or calibration perform reasonably well; red: both discrimination and calibration do not perform well. Prev. = Prevalence; HF = Heart failure.

|  | **Explorys (n = 4,750,660)** | | **MGB (n = 174,644)** | | **UKBB (n = 445,329)** | |
| --- | --- | --- | --- | --- | --- | --- |
|  | **Concordance Index**  **> 0.7?** | **Calibration slope**  **0.7–1.3?** | **Concordance Index > 0.7?** | **Calibration**  **Slope**  **0.7–1.3?** | **Concordance**  **Index**  **> 0.7?** | **Calibration**  **Slope**  **0.7–1.3?** |
| **45–54** | Yes | Yes | No | Yes | Yes | No |
| **55–64** | No | Yes | No | Yes | No | Yes |
| **65–74** | No | Yes | No | Yes | No | Yes |
| **75–84** | No | No | No | No | - | |
| **85–90** | No | No | No | No |  |  |
| **Female-only** | Yes | Yes | Yes | Yes | Yes | Yes |
| **Male-only** | Yes | Yes | Yes | Yes | Yes | Yes |
| **Black-only** | Yes | Yes | Yes | Yes | Yes | Yes |
| **White-only** | Yes | Yes | Yes | Yes | Yes | Yes |
| **Prev. HF** | No | No | No | No | No | No |
| **Prev. Stroke** | No | No | No | No | No | Yes |

**Supplementary Table XI. Population percentages indicating acceptable levels of discrimination and calibration considering CHARGE-AF.** Concordance Index > 0.7 is considered as a good discrimination; calibration slope between 0.7 and 1.3 is considered good; green: both discrimination and calibration perform reasonably well; orange: discrimination or calibration perform reasonably well; red: both discrimination and calibration do not perform well. Prev. = Prevalence; HF = Heart failure.

|  | **Explorys (n = 4,750,660)** | **MGB (n = 174,644)** | **UKBB (n = 445,329)** |
| --- | --- | --- | --- |
| **45–54** | 27.7% | 34.1% | 31.1% |
| **55–64** | 30.4% | 33.5% | 45.9% |
| **65–74** | 24.5% | 21.9% | 23.0% |
| **75–84** | 16.4% | 9.0% | - |
| **85–90** | 1.0% | 1.6% |  |
| **Female-only** | 56.7% | 60.9% | 55.0% |
| **Male-only** | 43.3% | 39.1% | 45.0% |
| **Black-only** | 15.8% | 19.4% | 5.3% |
| **White-only** | 84.2% | 80.6% | 94.7% |
| **Prev. HF** | 3.7% | 1.9% | 0.4% |
| **Prev. Stroke** | 5.0% | 2.0% | 1.5% |

**Supplementary Table XII. Acceptable discrimination and calibration indications for subgroups considering PCE.** CI > 0.7 is considered as a good discrimination; calibration slope between 0.7 and 1.3 is considered good; green: both discrimination and calibration perform reasonably well; orange: discrimination or calibration perform reasonably well; red: both discrimination and calibration do not perform well. Prev. = Prevalence; HF = Heart failure.

|  |  | **Explorys (n = 3,656,680)** | | **MGB (n = 198,184)** | | **UKBB (n = 408,154)** | |
| --- | --- | --- | --- | --- | --- | --- | --- |
|  |  | **Concordance Index > 0.7?** | **Calibration slope 0.7–1.3?** | **Concordance Index > 0.7?** | **Calibration slope 0.7–1.3?** | **Concordance Index > 0.7?** | **Calibration slope 0.7–1.3?** |
| **Female-White** | **40–49** | No | No | No | Yes | Yes | Yes |
|  | **50–59** | No | No | No | Yes | No | Yes |
|  | **60–69** | No | No | No | Yes | No | Yes |
|  | **70–79** | No | No | No | No | No | No |
|  | **Prev. HF** | No | No | No | No | No | No |
| **Female-Black** | **40–49** | No | No | Yes | No | Yes | Yes |
|  | **50–59** | No | No | No | No | Yes | Yes |
|  | **60–69** | No | No | No | No | No | Yes |
|  | **70–79** | No | No | No | No | Yes | No |
|  | **Prev. HF** | No | No | No | No | No | No |
| **Male-White** | **40–49** | No | No | No | Yes | Yes | Yes |
|  | **50–59** | No | No | No | Yes | No | Yes |
|  | **60–69** | No | No | No | Yes | No | Yes |
|  | **70–79** | No | No | No | No | No | No |
|  | **Prev. HF** | No | No | No | No | No | No |
| **Male-Black** | **40–49** | No | Yes | Yes | Yes | Yes | No |
|  | **50–59** | No | No | No | Yes | No | No |
|  | **60–69** | No | No | No | No | No | Yes |
|  | **70–79** | No | No | No | Yes | No | No |
|  | **Prev. HF** | No | No | No | No | Yes | Yes |

**Supplementary Table XIII. Population percentages indicating acceptable levels of discrimination and calibration considering PCE.** Concordance Index > 0.7 is considered as a good discrimination; calibration slope between 0.7 and 1.3 is considered good; green: both discrimination and calibration perform reasonably well; orange: discrimination or calibration perform reasonably well; red: both discrimination and calibration do not perform well. Prev. = Prevalence; HF = Heart failure.

|  |  | **Explorys (n = 3,656,680)** | **MGB (n = 198,184)** | **UKBB (n = 408,154)** |
| --- | --- | --- | --- | --- |
| **Female-White** | **40–49** | 21.5% | 28.6% | 21.9% |
|  | **50–59** | 29.2% | 32.4% | 33.9% |
|  | **60–69** | 27.5% | 25.5% | 42.4% |
|  | **70–79** | 21.8% | 13.5% | 1.8% |
|  | **Prev. HF** | 2.8% | 1.1% | 0.2% |
| **Female-Black** | **40–49** | 28.2% | 37.5% | 42.2% |
|  | **50–59** | 32.4% | 33.4% | 35.7% |
|  | **60–69** | 24.1% | 19.1% | 21.1% |
|  | **70–79** | 15.2% | 10.0% | 1.0% |
|  | **Prev. HF** | 4.9% | 2.4% | 0.3% |
| **Male-White** | **40–49** | 21.8% | 30.0% | 22.5% |
|  | **50–59** | 30.2% | 31.3% | 31.9% |
|  | **60–69** | 27.9% | 25.3% | 43.6% |
|  | **70–79** | 20.1% | 13.3% | 2.1% |
|  | **Prev. HF** | 3.7% | 2.0% | 0.5% |
| **Male-Black** | **40–49** | 28.3% | 40.5% | 45.6% |
|  | **50–59** | 34.0% | 33.9% | 33.2% |
|  | **60–69** | 24.2% | 18.7% | 19.7% |
|  | **70–79** | 13.5% | 6.9% | 1.6% |
|  | **Prev. HF** | 6.5% | 3.0% | 0.3% |

| 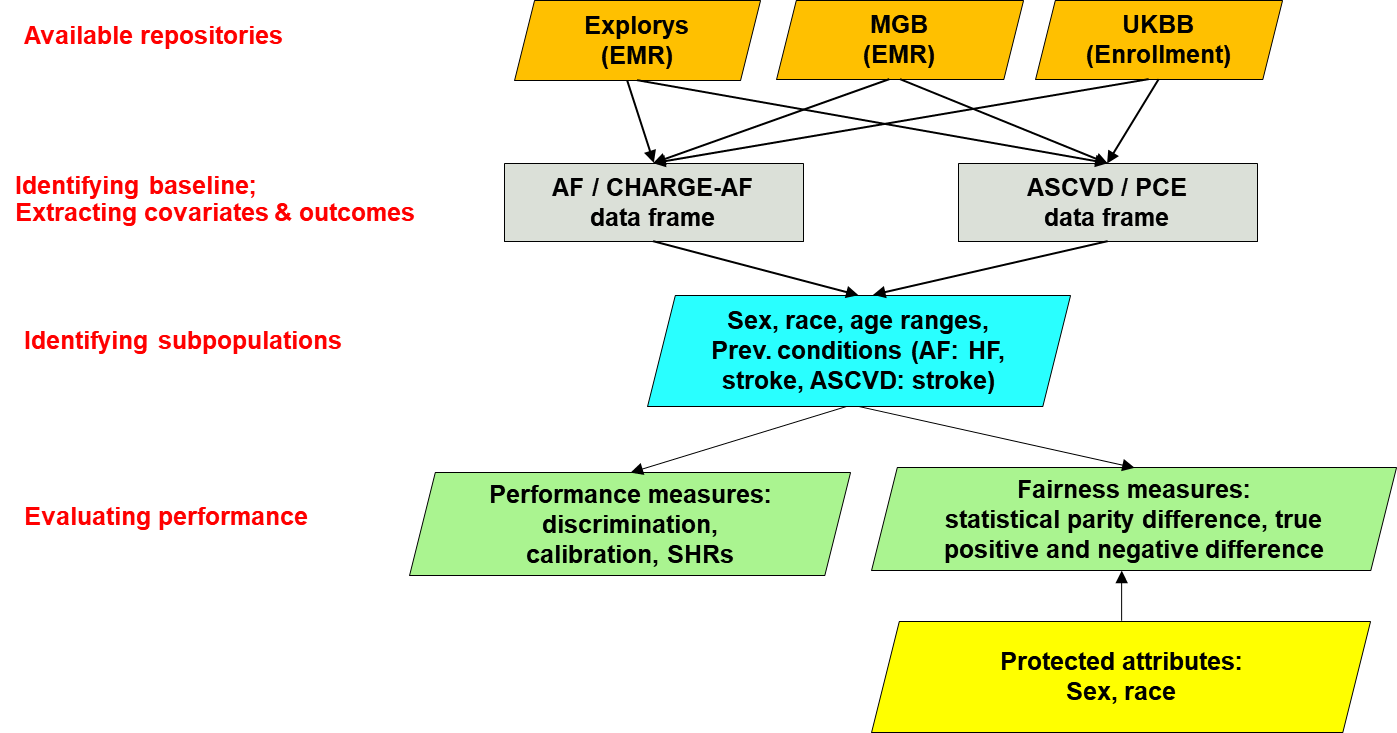 |
| --- |
| **Supplementary Figure 1. A high-level view of our methodology.** |
|  |
| **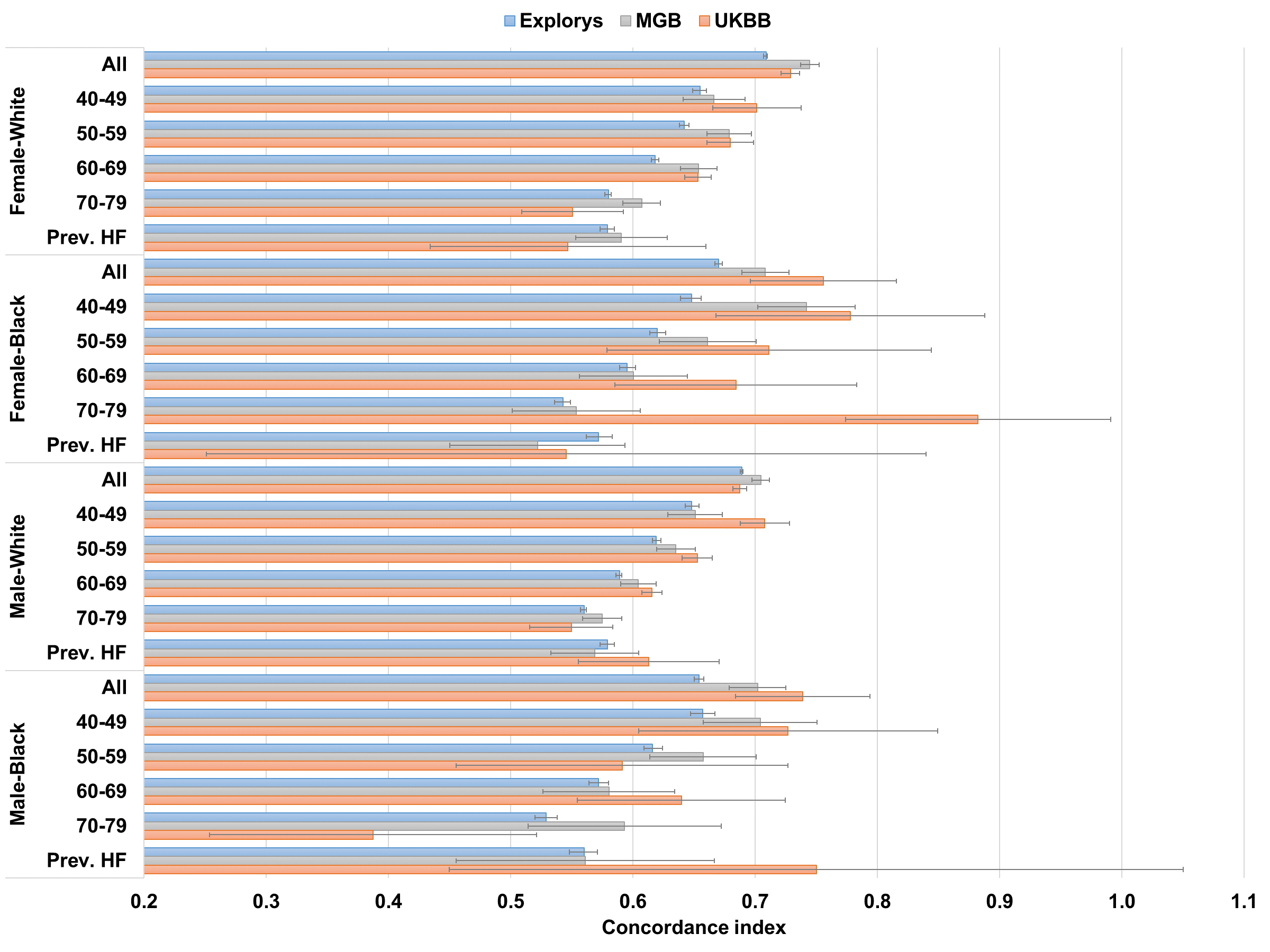** |
| **(a) Concordance index**. Considering the 2 largest populations (female-White and male-White) concordance indexes were decreasing with an increased age. This trend was more notable in the larger databases, Explorys, and MGB with smaller confidence interval values compared to the UKBB. Concordance indexes for the HF-prevalent groups in these two subpopulations had lower values compared to all other age groups. Due to small population sizes of the 2 Black subpopulations in the UKBB this trend was not clearly observed; for example, in the 70–79 female-Black group there were only 36 patients and in the male-Black group there were only 9 patients with prevalent HF. This finding was observed well, however, in Explorys and MGB. |
| **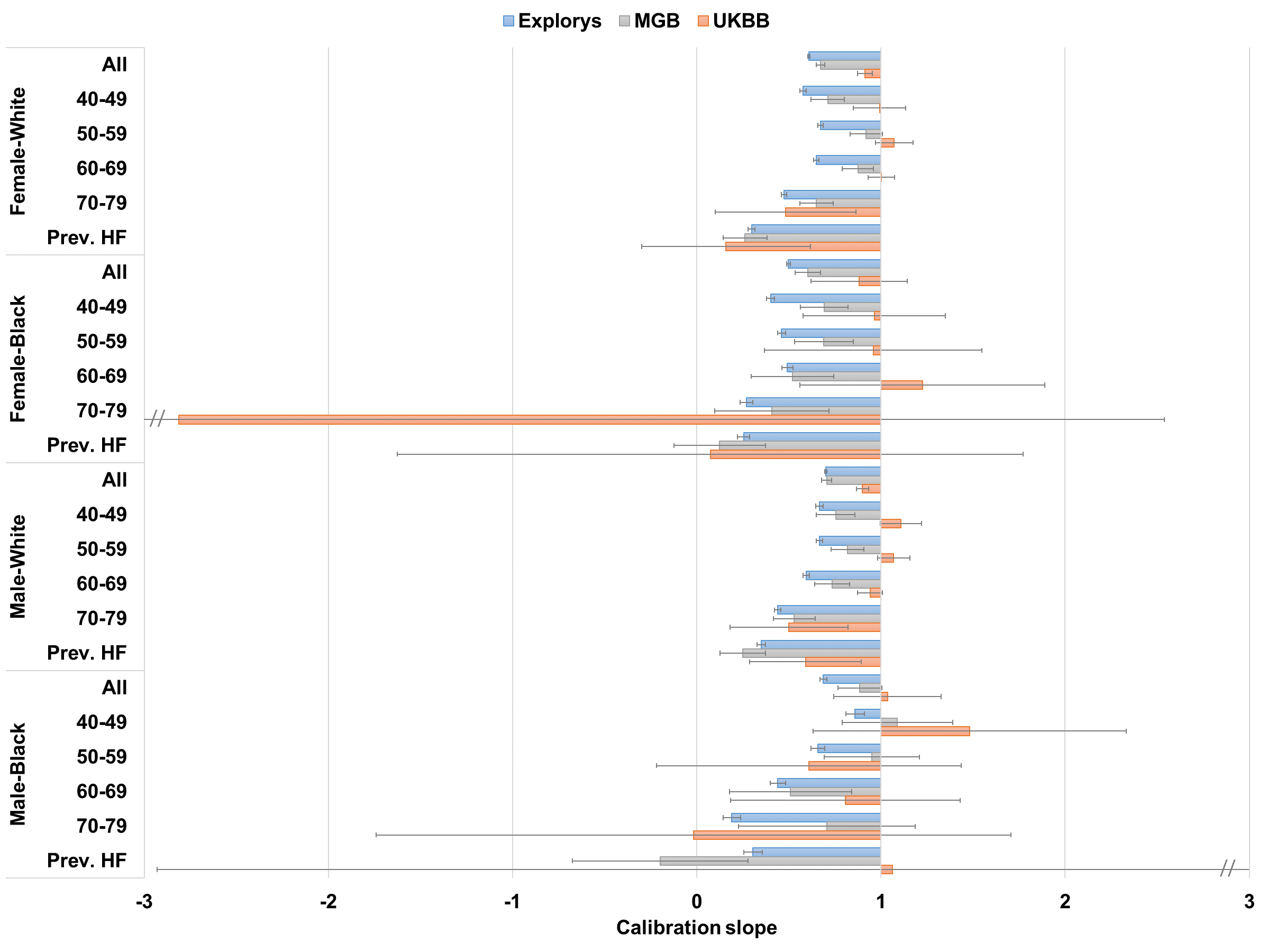** |
| **(b) Calibration slope.** Calibration values in close to all subpopulations of the 4 groups show an overestimation of the risk. In a few of the subpopulations risk seemed to be underestimated, however, this effect was observed primarily in the UKBB (e.g., male-White; 40–49 and 50–59, male-Black 40–49). In only one population MGB underestimated the risk – the male-Black 40–49 group, however, the size of this group was small (2,201 patients). Consistency of the findings in the largest group (female-White, n = 1,763,103) was observed also in the second largest group (male-White, n = 1,434,369 patients: overestimation was higher in the extreme age ranges). |
| **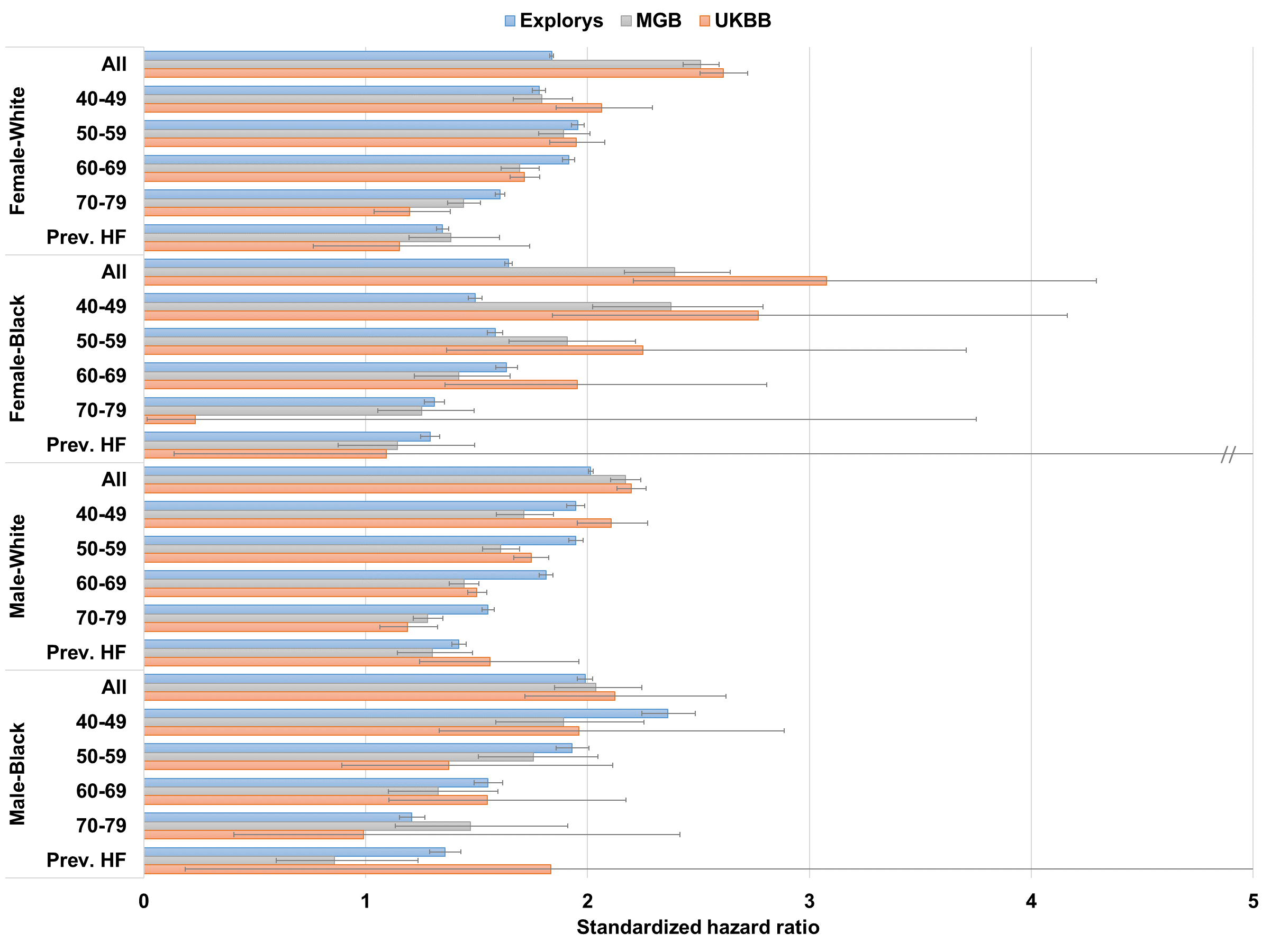** |
| **(c) Standardized hazard ratio.** SHRs were decreasing with age in close to all subpopulations. Notably SHRs for the HF-prevalent subpopulations were among the lowest, but typically close to or above the 70–79 subpopulations. |
| **Supplementary Figure 2. PCE analysis for the 4 models.** **(a)** Concordance index; **(b)** Calibration slope; **(c)** Standardized hazard ratio. All population and subpopulation sizes and exact incidence rates are provided in Supplementary Table VIII. |
